# Prenatal Care Initiation and Exposure to Teratogenic Medications

**DOI:** 10.1001/jamanetworkopen.2023.54298

**Published:** 2024-02-01

**Authors:** Almut G. Winterstein, Yanning Wang, Nicole E. Smolinski, Thuy N. Thai, Celeste Ewig, Sonja A. Rasmussen

**Affiliations:** 1 Pharmaceutical Outcomes & Policy, College of Pharmacy, University of Florida, Gainesville; 2 Center for Drug Evaluation and Safety, University of Florida, Gainesville; 3 Epidemiology, University of Florida, Gainesville; 4 Health Outcomes & Biomedical Informatics, University of Florida, Gainesville; 5 Faculty of Pharmacy, HUTECH University, Ho Chi Minh City, Vietnam; 6 Johns Hopkins University School of Medicine, Baltimore, Maryland

## Abstract

**Importance:**

With new legal abortion restrictions, timing of prenatal care initiation is critical to allow for discussion of reproductive options among pregnancies exposed to teratogenic medications.

**Objective:**

To investigate the prevalence of prenatal exposure to teratogenic medications and prenatal care initiation across gestational weeks.

**Design, Setting, and Participants:**

This descriptive, population-based cross-sectional study used health encounter data from a national sample of individuals with employer-sponsored health insurance. A validated algorithm identified pregnancies among persons identifying as female that ended with a live or nonlive outcome between January 2017 and December 2019. Data were analyzed from December 2022 to December 2023.

**Exposures:**

Prenatal exposure to any of 137 teratogenic medications, measured via pharmacy and medical claims. Measurement of prenatal care initiation was adapted from the Children’s Health Care Quality Measures.

**Main Outcomes and Measures:**

Prevalence of prenatal exposure to teratogens and prenatal care initiation by gestational week. Timing of prenatal teratogenic exposure was compared with timing of prenatal care initiation and legal abortion cutoffs.

**Results:**

Among 639 994 pregnancies, 472 472 (73.8%; 95% CI, 73.7%-73.9%) had a live delivery (mean [SD] age, 30.9 [5.4] years) and 167 522 (26.2%; 95% CI, 26.1%-26.3%) had a nonlive outcome (mean [SD] age, 31.6 [6.4] years). Of pregnancies with live deliveries, 5.8% (95% CI, 5.7%-5.8%) were exposed to teratogenic medications compared with 3.1% (95% CI, 3.0%-3.2%) with nonlive outcomes. Median time to prenatal care was 56 days (IQR, 44-70 days). By 6 weeks’ gestation, 8186 pregnancies had been exposed to teratogenic medications (25.2% [95% CI, 24.7%-25.7%] of pregnancies exposed at any time during gestation; 1.3% [95% CI, 1.3%-1.3%] of all pregnancies); in 6877 (84.0%; 95% CI, 83.2%-84.8%), prenatal care was initiated after 6 weeks or not at all. By 15 weeks, teratogenic exposures had occurred for 48.9% (95% CI, 48.4%-49.5%) of all teratogen-exposed pregnancies (2.5% [2.4-2.5] of all pregnancies); prenatal care initiation occurred after 15 weeks for 1810 (16.8%; 95% CI, 16.1%-17.5%) with live deliveries and 2975 (58.3%; 95% CI, 56.9%-59.6%) with nonlive outcomes. Teratogenic medications most used within the first 15 gestational weeks among live deliveries included antiinfectives (eg, fluconazole), anticonvulsants (eg, valproate), antihypertensives (eg, lisinopril), and immunomodulators (eg, mycophenolate). For nonlive deliveries, most antihypertensives were replaced by vitamin A derivatives.

**Conclusions and Relevance:**

In this cross-sectional study, most exposures to teratogenic medications occurred in early pregnancy and before prenatal care initiation, precluding prenatal risk-benefit assessments. Prenatal care commonly occurred after strict legal abortion cutoffs, prohibiting consideration of pregnancy termination if concerns about teratogenic effects arose.

## Key Points

**Question:**

What are the prevalence and timing of prenatal exposure to teratogenic medications and prenatal care initiation relative to legal abortion cutoffs?

**Findings:**

In this cross-sectional study of 639 994 pregnancies, by 6 weeks’ gestation, 1.3% were exposed to teratogenic medications; of these, prenatal care was initiated in 3.8% before teratogen exposure and in 84.0% after 6 weeks. By 15 weeks, teratogenic exposures had occurred in 2.5% of pregnancies, with prenatal care initiated after 15 weeks in 30.1%.

**Meaning:**

Prenatal care commonly occurred after teratogen exposure and abortion cutoffs, prohibiting medication risk-benefit assessments and discussion of options, including pregnancy termination if concerns about teratogenic effects arose.

## Teaser

This cross-sectional study investigates the prevalence of prenatal exposure to teratogenic medications and of prenatal care initiation across gestational weeks relative to common timing cutoffs for legal abortion.

## Introduction

On June 24, 2022, the US Supreme Court ended the constitutional right to an abortion, giving individual states the authority to regulate abortion access. Since then, abortion laws in individual states have changed rapidly. By May 2023, 14 US states had fully banned abortion, sometimes without exceptions.^1,2^ Other states have instituted bans after certain gestational age (GA) cutoffs—some as early as 6 weeks. Understanding the potential effects of these bans in the context of teratogenic medication exposures and access to pregnancy termination is critical.

Teratogenic medications are frequently prescribed for persons of childbearing potential. About 1 in 16 pregnancies is exposed to a potentially teratogenic medication, with highest exposure levels during the first trimester.^3^ For a few medications, the US Food and Drug Administration requires risk evaluation and mitigation strategies (REMSs)—safety programs that are designed to reinforce safe use behaviors.^4^ For example, iPLEDGE, the REMS for isotretinoin, requires patients to receive education, use effective contraception, and undergo pregnancy testing before receiving isotretinoin.^5^ Despite these strategies, isotretinoin-exposed pregnancies do occur.^6,7^

For pregnancies in which efforts to prevent exposure to teratogenic medications have failed, early initiation of prenatal care is essential to allow discussion of the potential risks, pregnancy monitoring for teratogenic effects, and discussion of options including pregnancy termination.^8,9^ For example, among 2720 pregnancies documented among more than 4 million patients who registered in the iPLEDGE program since its inception, 46% resulted in elective termination, 4.6% resulted in a live delivery, and 33.8% were lost to follow-up^10^; these data contrast with the elective abortion rates reported for the general population in the US (<20%).^11^ However, recognition of pregnancy onset varies, with a mean time to detection between 5 and 7 weeks’ gestation,^12,13^ resulting in delayed prenatal care initiation and a corresponding delay in teratogenic exposure mitigation. In this study, we estimated prenatal exposure rates to known teratogenic medications relative to the timing of prenatal care initiation and new timing windows for legal abortion.

## Methods

### Study Population

In this cross-sectional study, we identified pregnancy episodes among persons identifying as female in the MarketScan Commercial Database, which provides billing data for a national sample of US individuals with employer-sponsored health insurance. The University of Florida institutional review board considered the project exempt from approval and informed consent because it was not considered human participant research due to use of deidentified data. This study followed the Strengthening the Reporting of Observational Studies in Epidemiology (STROBE) reporting guideline.^14^

The MarketScan database allows longitudinal follow-up on health care utilization, including details on health plan enrollment, diagnoses, and procedures associated with outpatient and inpatient encounters and outpatient pharmacy dispensing. We considered pregnancies that ended between January 2017 and December 2019 in which pregnant individuals had continuous health plan enrollment with medication coverage within 90 days before estimated conception and 30 days after pregnancy end. We used a previously developed algorithm to identify pregnancy episodes, including both live and nonlive outcomes (ectopic pregnancies, spontaneous or induced abortions, and stillbirths), as applied in earlier published studies.^15,16,17^ The algorithm uses validated measures of pregnancy end points and estimates GA to determine the date of the last menstrual period.^18,19,20,21,22^ A detailed description of the algorithm along with value sets is available on request.

### Prenatal Exposure to Teratogenic Medications

We evaluated exposure to 137 teratogenic medications that we identified through review of the Teratogen Information System^23^ and relevant drug monographs, excluding sex hormones, infertility treatments, and opioids (eTable in Supplement 1).^3^ We also excluded misoprostol and methotrexate, because of their multiple indications including abortion, and ergotamine derivatives, because of their use for postpartum hemorrhage.

Medication utilization was measured via National Drug Codes on pharmacy claims and medical encounter claims using Healthcare Common Procedure Coding System (HCPCS) codes. Exposure timing was based on pharmacy dispensing or medical encounter dates. To focus on teratogenic risk, we excluded the 2 weeks between the last menstrual period and conception from the analysis. Determination of prenatal exposure to teratogenic medications considered the etiologically relevant trimester (eg, we omitted the first trimester for angiotensin converting enzyme inhibitors).^24,25^ In a sensitivity analysis, we expanded exposure measures to consider the entire window of dispensed days’ supply or the expected duration of action for medications administered during clinic visits (eg, prescription fills before conception when the days’ supply extended into pregnancy were considered prenatal exposures).

### First Prenatal Visits

We identified prenatal care initiation based on encounters with obstetricians, prenatal care practitioners, or primary care practitioners that were coded with specific stand-alone procedure codes (*Current Procedural Terminology* or HCPCS) indicating prenatal care or that included a diagnosis code for pregnancy. This definition was adapted from a measure in the Child Core Set of the Children’s Health Care Quality Measures for Medicaid and the Children’s Health Insurance Program, which assesses prenatal care initiation during the first trimester (eAppendix in Supplement 1).^26^ The assessed gestational time window included conception to the day before pregnancy end.

### Statistical Analysis

We summarized the number of pregnancy episodes exposed to teratogenic medications and the timing of initiation of prenatal care considering 3 cutoffs commonly used in current state abortion laws: 6, 15, and 22 weeks’ gestation. For each gestational period, we categorized pregnancies with teratogen exposure regarding timing of prenatal care initiation as the first prenatal care visit before teratogen exposure, the first prenatal care visit after teratogen exposure but within the evaluated gestational period (ie, the period between conception and the given gestational week), or the first prenatal care visit after the evaluated gestational period or no prenatal care (eFigure 1 in Supplement 1). We further described the timing of the first prenatal care visit compared with the first teratogen exposure by medication class. To account for differential follow-up periods when comparing pregnancies with live and nonlive outcomes, we added a sensitivity analysis in which we only included pregnancies that were viable at a given gestational week. Data management and statistical analyses were conducted from December 2022 to December 2023 using SAS, version 9.4 (SAS Institute Inc).

## Results

A total of 639 994 pregnancies were available for analysis, of which 472 472 (73.8%; 95% CI, 73.7%-73.9%) were live deliveries. The mean (SD) age among persons with live and nonlive pregnancy outcomes was 30.9 (5.4) years and 31.6 (6.4) years, respectively. Among pregnancies with live deliveries, 27 286 (5.8%; 95% CI, 5.7%-5.8%) had prenatal exposure to teratogenic medications during relevant risk windows (Table 1 and eFigures 2 and 3 in Supplement 1). Among the 167 522 pregnancies with nonlive outcomes (26.2%; 95% CI, 26.1%-26.3%), 5214 (3.1%; 95% CI, 3.0%-3.2%) had relevant teratogen exposures. Restricting assessments to pregnancies that were viable at 12 weeks, 8555 of 472 472 live birth pregnancies (1.8%; 95% CI, 1.8%-1.8%) and 1075 of 32 870 nonlive birth pregnancies (3.3%; 95% CI, 3.1%-3.5%) had teratogenic exposures (eFigure 4 in Supplement 1). Teratogenic medications most often used during pregnancy included agents acting on the renin-angiotensin system, anticonvulsants, systemic antimycotics, antineoplastics, isotretinoin, and warfarin.

**Table 1.** Exposures to Teratogenic Medications by Pregnancy Outcome and Exposure Definition^a^

Outcome	Pregnancies, No./total No. (%) [95% CI]	
	Exposed to teratogens	Exposed to teratogens considering residual days’ supply or medication action
Live birth	27 286/472 472 (5.8) [5.7-5.8]	29 053/472 472 (6.1) [6.1-6.2]
Nonlive birth	5214/167 522 (3.1) [3.0-3.2]	6034/167 522 (3.6) [3.5-3.7]
Ectopic pregnancy	291/9311 (3.1) [2.8-3.5]	336/9311 (3.6) [3.2-4.0]
Induced abortion	1506/32 470 (4.6) [4.4-4.9]	1673/32 470 (5.2) [4.9-5.4]
Spontaneous abortion	3236/122 667 (2.6) [2.5-2.7]	3821/122 667 (3.1) [3.0-3.2]
Stillbirth	181/3074 (5.9) [5.1-6.8]	204/3074 (6.6) [5.8-7.6]
Total	32 500/639 994 (5.1) [5.0-5.1]	35 087/639 994 (5.5) [5.4-5.5]

^a^ Based on data from the MarketScan Commercial Database, 2017-2019.

Considering the residual days’ supply or extended medication effects from dispensing or administration before the start of the risk window, the number of exposed pregnancies increased to 29 053 of those with live deliveries (6.1%; 95% CI, 6.1%-6.2%) and 6034 of those with nonlive outcomes (3.6%; 95% CI, 3.5%-3.7%). Teratogenic exposure prevalence was slightly higher for induced than for spontaneous abortions (4.6% [95% CI, 4.4%-4.9%] vs 2.6% [95% CI, 2.6%-2.7%]).

**Figure 1.**
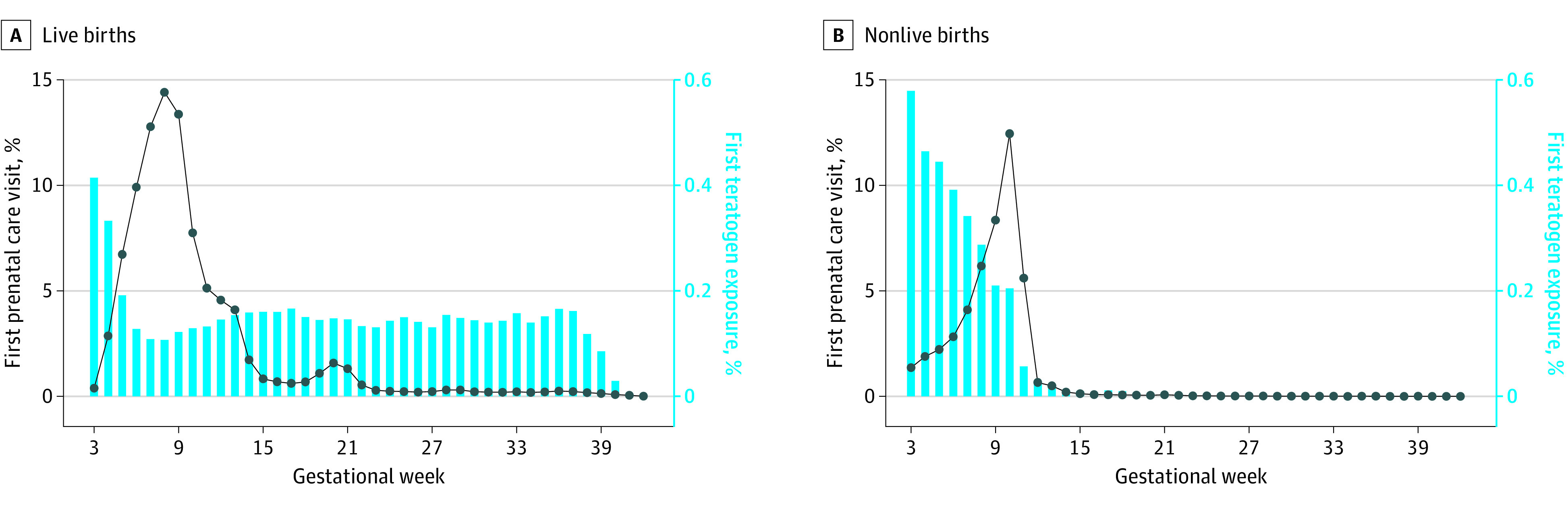
Prenatal Care Initiation and Teratogen Exposures by Gestational Week Based on data from the MarketScan Commercial Database, 2017-2019.

Among pregnancies with a live delivery, 448 581 (94.9%; 95% CI, 94.9%-95.0%) had a prenatal care visit compared with 78 791 pregnancies with nonlive outcomes (47.0%; 95% CI, 46.8%-47.3%) (Figure 1 and eFigure 2 in Supplement 1). Among pregnancies with prenatal care, median time to initiation was 56 days (IQR, 44-70 days). In 81.5% (95% CI, 81.3%-81.6%) of pregnancies with a live birth, a prenatal care visit occurred during the first trimester compared with 46.0% (95% CI, 45.8%-46.3%) of pregnancies with a nonlive birth.

A total of 8186 pregnancies were exposed to teratogenic medications during the first 6 gestational weeks, accounting for 25.2% (95% CI, 24.7%-25.7%) of all 32 500 pregnancies exposed at any time during gestation and 1.3% (95% CI, 1.3%-1.3%) of all pregnancies (Table 2). This number increased to 11 025 (1.7% [95% CI, 1.7%-1.8%] of all pregnancies) when considering days’ supply or medication action extending beyond conception (eFigure 3 in Supplement 1). At 15 weeks’ gestation, 15 898 pregnancies had been exposed to teratogenic medications, which accounted for 48.9% (95% CI, 48.4%-49.5%) of pregnancies exposed at any time during gestation and 2.5% (95% CI, 2.5%-2.5%) of all pregnancies. Considering residual days’ supply and duration of action of medications, this number increased to 18 885 (3.0% [95% CI, 2.9%-3.0%] of all pregnancies). Finally, exposure to teratogenic medications occurred within the first 22 gestational weeks in 20 926 pregnancies with such exposure (64.4%; 95% CI, 63.9%-64.9%), accounting for 3.3% (95% CI, 3.2%-3.3%) of all pregnancies. Considering residual days’ supply or medication action, the number of exposed pregnancies increased to 23 670 (3.7%; 95% CI, 3.7%-3.7% of all pregnancies).

**Table 2.** Pregnancies With Teratogen Exposure Within 6, 15, and 22 Weeks’ Gestation by Timing of Prenatal Care Initiation^a^

Prenatal care initiation	Pregnancies, No./total No. (%) [95% CI]		
	Live birth	Nonlive birth	Total
6 wk			
First prenatal care before first teratogen exposure	249/5037 (4.9) [4.4-5.6]	59/3149 (1.9) [1.5-2.4]	308/8186 (3.8) [3.4-4.2]
First prenatal care after first teratogen exposure but within 6 wk	895/5037 (17.8) [16.7-18.8]	106/3149 (3.4) [2.8-4.1]	1001/8186 (12.2) [11.5-13.0]
First prenatal care after 6 wk or no prenatal care	3893/5037 (77.3) [76.1-78.4]	2984/3149 (94.8) [93.9-95.5]	6877/8186 (84.0) [83.2-84.8]
15 wk			
First prenatal care before first teratogen exposure	4348/10 793 (40.3) [39.4-41.2]	465/5105 (9.1) [8.3-9.9]	4813/15 898 (30.3) [29.6-31.0]
First prenatal care after first teratogen exposure but within 15 wk	4635/10 793 (42.9) [42.0-43.9]	1665/5105 (32.6) [31.3-33.9]	6300/15 898 (39.6) [38.9-40.4]
First prenatal care after 15 wk or no prenatal care	1810/10 793 (16.8) [16.1-17.5]	2975/5105 (58.3) [56.9-59.6]	4785/15 898 (30.1) [29.4-30.8]
22 wk			
First prenatal care before first teratogen exposure	8612/15 749 (54.7) [53.9-55.5]	519/5177 (10.0) [9.2-10.9]	9131/20 926 (43.6) [43.0-44.3]
First prenatal care after first teratogen exposure but within 22 wk	5441/15 749 (34.5) [33.8-35.3]	1710/5177 (33.0) [31.8-34.3]	7151/20 926 (34.2) [33.5-34.8]
First prenatal care after 22 wk or no prenatal care	1696/15 749 (10.8) [10.3-11.3]	2948/5177 (56.9) [55.6-58.3]	4644/20 926 (22.2) [21.6-22.8]

^a^ Based on data from the MarketScan Commercial Database, 2017-2019.

Among pregnancies exposed during the first 6 gestational weeks, prenatal care was initiated before teratogen exposure in only 308 of 8186 (3.8%; 95% CI, 3.4%-4.2%), including 249 of 5037 pregnancies with a live birth (4.9%; 95% CI, 4.4%-5.6%) and 59 of 3149 with a nonlive birth (1.9%; 95% CI, 1.5%-2.4%). For the remainder of pregnancies exposed in the first 6 weeks, prenatal care started after the first 6 weeks or never occurred in 6877 (84.0%; 95% CI, 83.2%-84.8%)—3893 of 5037 with live deliveries (77.3%; 95% CI, 76.1%-78.4%) and 2984 of 3149 with nonlive deliveries (94.8%; 95% CI, 93.9%-95.5%).

Among the 15 898 pregnancies exposed within the first 15 gestational weeks, prenatal care started after 15 gestational weeks or never started for 4785 (30.1%; 95% CI, 29.4%-30.8%)—1810 of 10 793 with live deliveries (16.8%; 95% CI, 16.1%-17.5%) and 2975 of 5105 with nonlive deliveries (58.3%; 95% CI, 56.9%-59.6%). Finally, among pregnancies exposed to teratogenic medications within 22 weeks’ gestation, prenatal care was initiated before teratogen exposure in only 8612 of 15 749 with live deliveries (54.7%; 95% CI, 53.9%-55.5%) and was initiated after 22 weeks or never occurred in 1696 (10.8%; 95% CI, 10.3%-11.3%).

**Figure 2.**
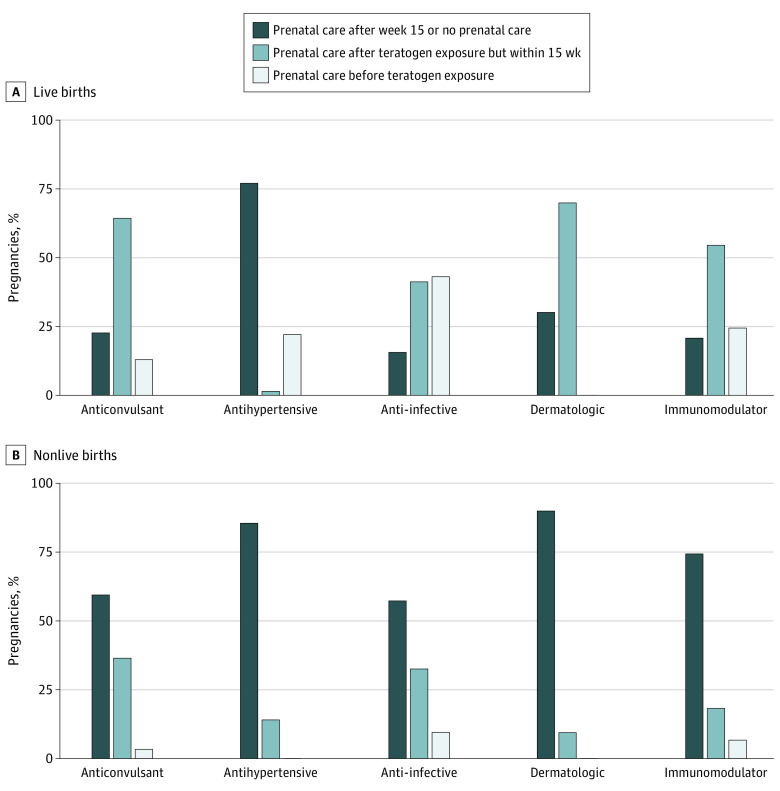
Pregnancies With Teratogen Exposure to the Top 5 Drug Classes Within 15 Weeks’ Gestation by Timing of Prenatal Care Based on data from the MarketScan Commercial Database, 2017-2019.

For live births, the teratogenic medications most used within the first 15 gestational weeks included antiinfectives (eg, fluconazole), anticonvulsants (eg, valproate), antihypertensives (eg, lisinopril), and immunomodulators (eg, mycophenolate). For nonlive deliveries, antihypertensives had limited representation due to their teratogenic risk window after the first trimester and were replaced by vitamin A derivatives (eg, isotretinoin). The sequencing of prenatal exposure and the first prenatal visit differed across these medication classes and by pregnancy outcome: only 103 of 793 pregnancies with live deliveries (13.0%; 95% CI, 10.8%-15.5%) and 19 of 526 with nonlive outcomes (3.6; 95% CI, 2.3%-5.6%) had a prenatal visit before exposure to a teratogenic anticonvulsant compared with 4197 of 9754 (43.0%; 95% CI, 42.0%-44.0%) and 439 of 4395 (10.0%; 95% CI, 9.1%-10.9%), respectively, before exposure to an antiinfective (Figure 2 and eFigure 5 in Supplement 1). We found that none of the 69 pregnancies exposed to vitamin A derivatives had a prenatal visit before the prescription fill.

## Discussion

This study assessed the timing of both prenatal care initiation and prenatal exposure to teratogens relative to current abortion laws. We considered 3 GA cutoffs to provide representative data on the frequency of 2 scenarios: prenatal exposure to a teratogen before prenatal care has been initiated, potentially precluding informed decision-making regarding the medication’s use during pregnancy, and initiation of prenatal care after both the teratogenic exposure and the legal window for abortion, prohibiting pregnancy termination as an option.

Three important findings of this study warrant discussion. First, in this population of privately insured individuals, almost 1 in 5 pregnancies with live outcomes (18.5%) and about half of pregnancies with nonlive outcomes (54.0%) did not initiate prenatal care during the first trimester, leaving close to no opportunity for medication management decisions involving teratogenic risk during the etiologically relevant time window for most teratogenic exposures. This is particularly relevant for chronically used medications that might be continued before pregnancy is recognized or new medication initiations without pregnancy assessments. For example, among isotretinoin-exposed pregnancies, 4.9% to 9.1% were found to be conceived before isotretinoin initiation, while 61.6% to 72.0% were conceived during isotretinoin treatment.^10^ Among patients who filled a new topiramate prescription, about 1 in 1000 was found to be pregnant at treatment initiation.^27^

Second, not surprisingly, a sizable proportion of teratogenic exposures occurred during early gestation. Therefore, most teratogenic exposures occurred before the first prenatal visit in almost all examined scenarios; even for teratogen exposures within 22 weeks, only half of live-birth pregnancies had prenatal care before exposure. Thus, informed discussions regarding treatment with teratogenic medications would not be feasible for most pregnancies. Such discussions might have evaluated the risks and benefits of a teratogenic medication as favorable, although decreases in prenatal exposure in later trimesters were noted in this study and in a previous study,^3^ suggesting that some exposures were unintended. This finding was further supported by the larger observed teratogen exposure frequencies among induced abortions compared with spontaneous abortions. The observed variation in the sequencing of prenatal care relative to teratogen exposure also suggests that exposure to chronically used teratogens more commonly precedes prenatal care than does use of acute treatments (antiinfectives).

Third, prenatal care typically occurred after not only prenatal exposure but also earlier abortion cutoffs. A total of 84.0% of pregnancies with exposure to teratogenic medications during the first 6 weeks of pregnancy did not have prenatal care within that period, and this proportion had only decreased to 30.1% of all pregnancies at 15 weeks’ gestation. These numbers are not surprising given that the mean GA at which pregnant persons become aware of their pregnancy is 6 weeks.^13^ While abortion bans may allow exceptions, such as in cases of rape or incest, most states with abortion bans do not allow exceptions for fetal anomalies identified prenatally.^28^ This implies that if the potential for a teratogenic effect becomes apparent, most pregnancies would be carried to delivery.^29^

To prevent such scenarios, persons who are planning a pregnancy require access to preconception care. Both the Centers for Disease Control and Prevention and the American College of Obstetricians and Gynecologists recommend preconception counseling, which can address exposures to teratogenic medications, care for chronic medical conditions, and other issues that could place a pregnancy at risk.^30,31^ Although not broadly implemented, preconception and pregnancy intention screening tools are available to support counseling.^32^ However, even among persons with a recent live birth who had diabetes or hypertension, placing them at increased risk for complications, less than half reported receiving preconception counseling.^33^ Furthermore, about half of pregnancies in the US are unplanned, and persons not planning a pregnancy would be less likely to get preconception care.^34^ In addition, pregnancy awareness varies, with the greatest delays in confirmation via home or clinic testing among teenagers, underrepresented minority populations, those with food insecurity, and those with unplanned pregnancies.^12,13^

Options to prevent prenatal exposure due to unrecognized pregnancies and to increase awareness that a teratogenic medication may need to be discontinued include REMS programs. Although not foolproof, REMS programs have demonstrated effectiveness in preventing initiation of teratogenic medications during pregnancy and, to a lesser extent, preventing conception during use.^15,27^ Concerns about REMS programs include administrative burden to practitioners and patients, potentially limiting access to important medications. Some might consider universal avoidance of teratogenic medications for persons of childbearing potential in light of the *Dobbs v Jackson Women’s Health Organization* decision; however, this raises serious concerns about health equity, especially for treatment of medical conditions that rely on such medications.^8,9^ Thus, approaches to prevent prenatal exposure to teratogenic medications must be enhanced and supported by research that highlights relevant root causes and evaluates the risks and benefits of REMS and other programs.

If prenatal exposure to teratogenic medications occurs, it is critical that prenatal care be initiated expeditiously to allow physician-patient discussions about pregnancy termination within legal abortion windows. Our data illustrate opportunities to improve appropriate timing of prenatal screening to enhance healthy pregnancies. They also emphasize that to allow pregnancy termination as an option, prenatal care should be initiated and options discussed when a person may suspect pregnancy and prenatal exposure to teratogens has occurred. This study’s data are important for consideration as new abortion laws and relevant exceptions are drafted.

### Strengths and Limitations

Several strengths of our analysis are noteworthy. Our analysis reflects medication utilization and prenatal care patterns of a national sample of pregnant individuals with private insurance. We used previously validated algorithms to determine pregnancy episodes and based measures of medication exposure on pharmacy claims and medical encounters.^22^ While a pharmacy claim does not prove exposure, our focus on prescription fills during the pregnancy risk window (commonly involving co-payments) may alleviate misclassification. To fully capture the potential impact of prenatal exposure, we also presented results incorporating residual days’ supply or medication action from prescription fills or administrations before the risk window.

This study also has limitations. We reported results for pregnancies that ended in both live and nonlive outcomes to allow comprehensive capture of teratogenic medication exposure during pregnancy, which was, as expected, higher among nonlive pregnancies. Although we considered a broad spectrum of pregnancy outcomes, our algorithm might have missed pregnancies that never resulted in reimbursed clinical care (eg, with an elective abortion that was paid by the patient as the only pregnancy-related health care service).

Timing of prenatal exposure further relies on accurate timing of conception. While pharmacoepidemiologic drug safety studies in pregnancy have made great advances in the accurate estimation of GA and, thus, conception,^19,22,35^ error margins, especially for pregnancies with nonlive outcomes, deserve consideration. Of particular concern would be overestimates of GA, which could result in overestimates of prenatal exposure if persons discontinued therapy shortly before conception. We addressed this concern by restricting our study period only to the era of the *International Statistical Classification of Diseases, 10th Revision, Clinical Modification*, which offers enhanced GA coding; by considering timing of prenatal screening procedures as a GA proxy; and in the absence of direct GA codes or proxies, by defaulting to a small GA estimate of 10 weeks for spontaneous or induced abortions, 8 weeks for ectopic pregnancies, and 28 weeks for stillbirths. This approach aligns with a previously validated algorithm with the following respective percentages of agreement of GA estimates within 7 and 28 days when adjudicated against medical records: full-term live births, 86% and 100%; preterm live births, 82% and 99%; and spontaneous abortions, 61% and 95%.^22^ Our research group has successfully used similar GA estimation techniques in medication safety studies^16,17^ and for the assessment of drug-drug interactions regarding risk for hormonal contraceptive failure, which greatly relies on accurate estimation of conception because contraception is terminated as soon as pregnancy is recognized.^36^

To measure prenatal care, we used a modified algorithm that has been developed for quality assessments in Medicaid programs. Using this algorithm, we found prenatal care rates similar to national estimates (81.5% of live births with prenatal care initiation within the first trimester compared with 87.0% for privately insured persons based on birth certificate data).^37^ It is possible that pregnant persons communicated with their practitioner prior to their first prenatal care visits via telephone or messaging, which might be obscured in our database, although such interaction would unlikely comprise discussion of teratogenic risk and pregnancy termination. Continuation of teratogenic medications during pregnancy also might have been intentional after careful consideration of risks and benefits, although most medications that we found to be commonly involved in prenatal exposure have indications for which alternative nonteratogenic treatment options exist.

Because uninsured persons commonly become eligible for Medicaid because of pregnancy, both medication utilization and the initiation of prenatal care in this population are expected to be different. Thus, analyses should be expanded to comprehensively capture the effects of strict abortion laws in this population. Future research is needed to quantify the public health impact of prenatal exposure to teratogenic medications and the effects of abortion laws on both exposure rates and adverse outcomes.

## Conclusions

In this cross-sectional study of a national sample of privately insured individuals in the US, we found that a sizable proportion of prenatal exposure to teratogenic medications occurred during early pregnancy and before initiation of prenatal care, precluding risk-benefit assessments during pregnancy. Initiation of prenatal care after teratogenic exposure often occurred after strict GA cutoffs set in new abortion laws in many states, precluding pregnancy monitoring and discussion of pregnancy termination if concerns about teratogenic effects arose.

## Notes

Supplement 1. **eAppendix.** Diagnoses, Procedures, and Practitioner Types Used to Identify a Prenatal Care Visit **eTable.** List of Definite Teratogenic Medications **eFigure 1.** Assessments of Timing of Prenatal Care Initiation Relative to Prenatal Exposure to Teratogenic Medications **eFigure 2.** Timing of Prenatal Care Initiation and Prenatal Exposure to Teratogenic Medications **eFigure 3.** Timing of Prenatal Care Initiation for Pregnancies Exposed to Teratogenic Medications Considering Any Pregnancy **eFigure 4.** Timing of Prenatal Care Initiation for Pregnancies Exposed to Teratogenic Medications Considering Pregnancies Viable Up to the Assessed Gestational Week **eFigure 5.** Timing of Prenatal Care Initiation for Pregnancies Exposed to Teratogenic Medications by Drug Class

Supplement 2.
Data Sharing Statement
