# Supplementary material for: Prenatal Care Initiation and Exposure to Teratogenic Medications
Source: JAMA Netw Open. 2024 Feb 1;7(2):e2354298. doi: 10.1001/jamanetworkopen.2023.54298 (PMC10835507; doi:10.1001/jamanetworkopen.2023.54298)
Supplement: Supplement 1. — eAppendix. Diagnoses, Procedures, and Practitioner Types Used to Identify a Prenatal Care Visit eTable. List of Definite Teratogenic Medications eFigure 1. Assessments of Timing of Prenatal Care Initiation Relative to Prenatal Exposure to Teratogenic Medications eFigure 2. Timing of Prenatal Care Initiation and Prenatal Exposure to Teratogenic Medications eFigure 3. Timing of Prenatal Care Initiation for Pregnancies Exposed to Teratogenic Medications Considering Any Pregnancy eFigure 4. Timing of Prenatal Care Initiation for Pregnancies Exposed to Teratogenic Medications Considering Pregnancies Viable Up to the Assessed Gestational Week eFigure 5. Timing of Prenatal Care Initiation for Pregnancies Exposed to Teratogenic Medications by Drug Class [file jamanetwopen-e2354298-s001.pdf]

Supplementary Online Content

Winterstein AG, Wang Y, Smolinski NE, Thai TN, Ewig C, Rasmussen SA. Prenatal care initiation and exposure to teratogenic medications. *JAMA Netw Open*. 2024;7(2):e2354298. doi:10.1001/jamanetworkopen.2023.54298

eAppendix. Diagnoses, Procedures, and Practitioner Types Used to Identify a Prenatal Care Visit

eTable. List of Definite Teratogenic Medications

eFigure 1. Assessments of Timing of Prenatal Care Initiation Relative to Prenatal Exposure to Teratogenic Medications

eFigure 2. Timing of Prenatal Care Initiation and Prenatal Exposure to Teratogenic Medications

eFigure 3. Timing of Prenatal Care Initiation for Pregnancies Exposed to Teratogenic Medications Considering Any Pregnancy

eFigure 4. Timing of Prenatal Care Initiation for Pregnancies Exposed to Teratogenic Medications Considering Pregnancies Viable Up to the Assessed Gestational Week

eFigure 5. Timing of Prenatal Care Initiation for Pregnancies Exposed to Teratogenic Medications by Drug Class

This supplementary material has been provided by the authors to give readers additional information about their work.

© 2024 Winterstein AG et al. *JAMA Network Open*.

eAppendix. Diagnoses, procedures, and practitioner types used to identify a prenatal care visit

Pregnancy Diagnosis Codes (International Classification of Diseases, Tenth Revision, Clinical Modification)

[O09.00] Supervision of pregnancy with history of infertility, unspecified trimester
[O09.01] Supervision of pregnancy with history of infertility, first trimester
[O09.02] Supervision of pregnancy with history of infertility, second trimester
[O09.03] Supervision of pregnancy with history of infertility, third trimester
[O09.10] Supervision of pregnancy with history of ectopic pregnancy, unspecified trimester
[O09.11] Supervision of pregnancy with history of ectopic pregnancy, first trimester
[O09.12] Supervision of pregnancy with history of ectopic pregnancy, second trimester
[O09.13] Supervision of pregnancy with history of ectopic pregnancy, third trimester
[O09.211] Supervision of pregnancy with history of pre-term labor, first trimester
[O09.212] Supervision of pregnancy with history of pre-term labor, second trimester
[O09.213] Supervision of pregnancy with history of pre-term labor, third trimester
[O09.219] Supervision of pregnancy with history of pre-term labor, unspecified trimester
[O09.291] Supervision of pregnancy with other poor reproductive or obstetric history, first trimester
[O09.292] Supervision of pregnancy with other poor reproductive or obstetric history, second trimester
[O09.293] Supervision of pregnancy with other poor reproductive or obstetric history, third trimester
[O09.299] Supervision of pregnancy with other poor reproductive or obstetric history, unspecified trimester
[O09.30] Supervision of pregnancy with insufficient antenatal care, unspecified trimester
[O09.31] Supervision of pregnancy with insufficient antenatal care, first trimester
[O09.32] Supervision of pregnancy with insufficient antenatal care, second trimester
[O09.33] Supervision of pregnancy with insufficient antenatal care, third trimester
[O09.40] Supervision of pregnancy with grand multiparity, unspecified trimester
[O09.41] Supervision of pregnancy with grand multiparity, first trimester
[O09.42] Supervision of pregnancy with grand multiparity, second trimester
[O09.43] Supervision of pregnancy with grand multiparity, third trimester
[O09.511] Supervision of elderly primigravida, first trimester
[O09.512] Supervision of elderly primigravida, second trimester
[O09.513] Supervision of elderly primigravida, third trimester
[O09.519] Supervision of elderly primigravida, unspecified trimester
[O09.521] Supervision of elderly multigravida, first trimester
[O09.522] Supervision of elderly multigravida, second trimester
[O09.523] Supervision of elderly multigravida, third trimester
[O09.529] Supervision of elderly multigravida, unspecified trimester
[O09.611] Supervision of young primigravida, first trimester

© 2024 Winterstein AG et al. *JAMA Network Open*.

[O09.612] Supervision of young primigravida, second trimester
[O09.613] Supervision of young primigravida, third trimester
[O09.619] Supervision of young primigravida, unspecified trimester
[O09.621] Supervision of young multigravida, first trimester
[O09.622] Supervision of young multigravida, second trimester
[O09.623] Supervision of young multigravida, third trimester
[O09.629] Supervision of young multigravida, unspecified trimester
[O09.70] Supervision of high risk pregnancy due to social problems, unspecified trimester
[O09.71] Supervision of high risk pregnancy due to social problems, first trimester
[O09.72] Supervision of high risk pregnancy due to social problems, second trimester
[O09.73] Supervision of high risk pregnancy due to social problems, third trimester
[O09.811] Supervision of pregnancy resulting from assisted reproductive technology, first trimester
[O09.812] Supervision of pregnancy resulting from assisted reproductive technology, second trimester
[O09.813] Supervision of pregnancy resulting from assisted reproductive technology, third trimester
[O09.819] Supervision of pregnancy resulting from assisted reproductive technology, unspecified trimester
[O09.821] Supervision of pregnancy with history of in utero procedure during previous pregnancy, first trimester
[O09.822] Supervision of pregnancy with history of in utero procedure during previous pregnancy, second trimester
[O09.823] Supervision of pregnancy with history of in utero procedure during previous pregnancy, third trimester
[O09.829] Supervision of pregnancy with history of in utero procedure during previous pregnancy, unspecified trimester
[O09.891] Supervision of other high risk pregnancies, first trimester
[O09.892] Supervision of other high risk pregnancies, second trimester
[O09.893] Supervision of other high risk pregnancies, third trimester
[O09.899] Supervision of other high risk pregnancies, unspecified trimester
[O09.90] Supervision of high risk pregnancy, unspecified, unspecified trimester
[O09.91] Supervision of high risk pregnancy, unspecified, first trimester
[O09.92] Supervision of high risk pregnancy, unspecified, second trimester
[O09.93] Supervision of high risk pregnancy, unspecified, third trimester
[O09.A0] Supervision of pregnancy with history of molar pregnancy, unspecified trimester
[O09.A1] Supervision of pregnancy with history of molar pregnancy, first trimester
[O09.A2] Supervision of pregnancy with history of molar pregnancy, second trimester
[O09.A3] Supervision of pregnancy with history of molar pregnancy, third trimester
[O10.011] Pre-existing essential hypertension complicating pregnancy, first trimester
[O10.012] Pre-existing essential hypertension complicating pregnancy, second trimester
[O10.013] Pre-existing essential hypertension complicating pregnancy, third trimester
[O10.019] Pre-existing essential hypertension complicating pregnancy, unspecified trimester
[O10.111] Pre-existing hypertensive heart disease complicating pregnancy, first trimester

© 2024 Winterstein AG et al. *JAMA Network Open*.

[O10.112] Pre-existing hypertensive heart disease complicating pregnancy, second trimester
[O10.113] Pre-existing hypertensive heart disease complicating pregnancy, third trimester
[O10.119] Pre-existing hypertensive heart disease complicating pregnancy, unspecified trimester
[O10.211] Pre-existing hypertensive chronic kidney disease complicating pregnancy, first trimester
[O10.212] Pre-existing hypertensive chronic kidney disease complicating pregnancy, second trimester
[O10.213] Pre-existing hypertensive chronic kidney disease complicating pregnancy, third trimester
[O10.219] Pre-existing hypertensive chronic kidney disease complicating pregnancy, unspecified trimester
[O10.311] Pre-existing hypertensive heart and chronic kidney disease complicating pregnancy, first trimester
[O10.312] Pre-existing hypertensive heart and chronic kidney disease complicating pregnancy, second trimester
[O10.313] Pre-existing hypertensive heart and chronic kidney disease complicating pregnancy, third trimester
[O10.319] Pre-existing hypertensive heart and chronic kidney disease complicating pregnancy, unspecified trimester
[O10.411] Pre-existing secondary hypertension complicating pregnancy, first trimester
[O10.412] Pre-existing secondary hypertension complicating pregnancy, second trimester
[O10.413] Pre-existing secondary hypertension complicating pregnancy, third trimester
[O10.419] Pre-existing secondary hypertension complicating pregnancy, unspecified trimester
[O10.911] Unspecified pre-existing hypertension complicating pregnancy, first trimester
[O10.912] Unspecified pre-existing hypertension complicating pregnancy, second trimester
[O10.913] Unspecified pre-existing hypertension complicating pregnancy, third trimester
[O10.919] Unspecified pre-existing hypertension complicating pregnancy, unspecified trimester
[O11.1] Pre-existing hypertension with pre-eclampsia, first trimester
[O11.2] Pre-existing hypertension with pre-eclampsia, second trimester
[O11.3] Pre-existing hypertension with pre-eclampsia, third trimester
[O11.9] Pre-existing hypertension with pre-eclampsia, unspecified trimester
[O12.00] Gestational edema, unspecified trimester
[O12.01] Gestational edema, first trimester
[O12.02] Gestational edema, second trimester
[O12.03] Gestational edema, third trimester
[O12.10] Gestational proteinuria, unspecified trimester
[O12.11] Gestational proteinuria, first trimester
[O12.12] Gestational proteinuria, second trimester
[O12.13] Gestational proteinuria, third trimester
[O12.20] Gestational edema with proteinuria, unspecified trimester
[O12.21] Gestational edema with proteinuria, first trimester
[O12.22] Gestational edema with proteinuria, second trimester
[O12.23] Gestational edema with proteinuria, third trimester
[O13.1] Gestational [pregnancy-induced] hypertension without significant proteinuria, first trimester

© 2024 Winterstein AG et al. *JAMA Network Open*.

[O13.2] Gestational [pregnancy-induced] hypertension without significant proteinuria, second trimester
[O13.3] Gestational [pregnancy-induced] hypertension without significant proteinuria, third trimester
[O13.9] Gestational [pregnancy-induced] hypertension without significant proteinuria, unspecified trimester
[O14.00] Mild to moderate pre-eclampsia, unspecified trimester
[O14.02] Mild to moderate pre-eclampsia, second trimester
[O14.03] Mild to moderate pre-eclampsia, third trimester
[O14.10] Severe pre-eclampsia, unspecified trimester
[O14.12] Severe pre-eclampsia, second trimester
[O14.13] Severe pre-eclampsia, third trimester
[O14.20] HELLP syndrome (HELLP), unspecified trimester
[O14.22] HELLP syndrome (HELLP), second trimester
[O14.23] HELLP syndrome (HELLP), third trimester
[O14.90] Unspecified pre-eclampsia, unspecified trimester
[O14.92] Unspecified pre-eclampsia, second trimester
[O14.93] Unspecified pre-eclampsia, third trimester
[O15.00] Eclampsia complicating pregnancy, unspecified trimester
[O15.02] Eclampsia complicating pregnancy, second trimester
[O15.03] Eclampsia complicating pregnancy, third trimester
[O15.1] Eclampsia complicating labor
[O15.9] Eclampsia, unspecified as to time period
[O16.1] Unspecified maternal hypertension, first trimester
[O16.2] Unspecified maternal hypertension, second trimester
[O16.3] Unspecified maternal hypertension, third trimester
[O16.9] Unspecified maternal hypertension, unspecified trimester
[O20.0] Threatened abortion
[O20.8] Other hemorrhage in early pregnancy
[O20.9] Hemorrhage in early pregnancy, unspecified
[O21.0] Mild hyperemesis gravidarum
[O21.1] Hyperemesis gravidarum with metabolic disturbance
[O21.2] Late vomiting of pregnancy
[O21.8] Other vomiting complicating pregnancy
[O21.9] Vomiting of pregnancy, unspecified
[O22.00] Varicose veins of lower extremity in pregnancy, unspecified trimester
[O22.01] Varicose veins of lower extremity in pregnancy, first trimester
[O22.02] Varicose veins of lower extremity in pregnancy, second trimester
[O22.03] Varicose veins of lower extremity in pregnancy, third trimester

© 2024 Winterstein AG et al. *JAMA Network Open*.

[O22.10] Genital varices in pregnancy, unspecified trimester
[O22.11] Genital varices in pregnancy, first trimester
[O22.12] Genital varices in pregnancy, second trimester
[O22.13] Genital varices in pregnancy, third trimester
[O22.20] Superficial thrombophlebitis in pregnancy, unspecified trimester
[O22.21] Superficial thrombophlebitis in pregnancy, first trimester
[O22.22] Superficial thrombophlebitis in pregnancy, second trimester
[O22.23] Superficial thrombophlebitis in pregnancy, third trimester
[O22.30] Deep phlebothrombosis in pregnancy, unspecified trimester
[O22.31] Deep phlebothrombosis in pregnancy, first trimester
[O22.32] Deep phlebothrombosis in pregnancy, second trimester
[O22.33] Deep phlebothrombosis in pregnancy, third trimester
[O22.40] Hemorrhoids in pregnancy, unspecified trimester
[O22.41] Hemorrhoids in pregnancy, first trimester
[O22.42] Hemorrhoids in pregnancy, second trimester
[O22.43] Hemorrhoids in pregnancy, third trimester
[O22.50] Cerebral venous thrombosis in pregnancy, unspecified trimester
[O22.51] Cerebral venous thrombosis in pregnancy, first trimester
[O22.52] Cerebral venous thrombosis in pregnancy, second trimester
[O22.53] Cerebral venous thrombosis in pregnancy, third trimester
[O22.8X1] Other venous complications in pregnancy, first trimester
[O22.8X2] Other venous complications in pregnancy, second trimester
[O22.8X3] Other venous complications in pregnancy, third trimester
[O22.8X9] Other venous complications in pregnancy, unspecified trimester
[O22.90] Venous complication in pregnancy, unspecified, unspecified trimester
[O22.91] Venous complication in pregnancy, unspecified, first trimester
[O22.92] Venous complication in pregnancy, unspecified, second trimester
[O22.93] Venous complication in pregnancy, unspecified, third trimester
[O23.00] Infections of kidney in pregnancy, unspecified trimester
[O23.01] Infections of kidney in pregnancy, first trimester
[O23.02] Infections of kidney in pregnancy, second trimester
[O23.03] Infections of kidney in pregnancy, third trimester
[O23.10] Infections of bladder in pregnancy, unspecified trimester
[O23.11] Infections of bladder in pregnancy, first trimester
[O23.12] Infections of bladder in pregnancy, second trimester
[O23.13] Infections of bladder in pregnancy, third trimester

© 2024 Winterstein AG et al. *JAMA Network Open*.

[O23.20] Infections of urethra in pregnancy, unspecified trimester
[O23.21] Infections of urethra in pregnancy, first trimester
[O23.22] Infections of urethra in pregnancy, second trimester
[O23.23] Infections of urethra in pregnancy, third trimester
[O23.30] Infections of other parts of urinary tract in pregnancy, unspecified trimester
[O23.31] Infections of other parts of urinary tract in pregnancy, first trimester
[O23.32] Infections of other parts of urinary tract in pregnancy, second trimester
[O23.33] Infections of other parts of urinary tract in pregnancy, third trimester
[O23.40] Unspecified infection of urinary tract in pregnancy, unspecified trimester
[O23.41] Unspecified infection of urinary tract in pregnancy, first trimester
[O23.42] Unspecified infection of urinary tract in pregnancy, second trimester
[O23.43] Unspecified infection of urinary tract in pregnancy, third trimester
[O23.511] Infections of cervix in pregnancy, first trimester
[O23.512] Infections of cervix in pregnancy, second trimester
[O23.513] Infections of cervix in pregnancy, third trimester
[O23.519] Infections of cervix in pregnancy, unspecified trimester
[O23.521] Salpingo-oophoritis in pregnancy, first trimester
[O23.522] Salpingo-oophoritis in pregnancy, second trimester
[O23.523] Salpingo-oophoritis in pregnancy, third trimester
[O23.529] Salpingo-oophoritis in pregnancy, unspecified trimester
[O23.591] Infection of other part of genital tract in pregnancy, first trimester
[O23.592] Infection of other part of genital tract in pregnancy, second trimester
[O23.593] Infection of other part of genital tract in pregnancy, third trimester
[O23.599] Infection of other part of genital tract in pregnancy, unspecified trimester
[O23.90] Unspecified genitourinary tract infection in pregnancy, unspecified trimester
[O23.91] Unspecified genitourinary tract infection in pregnancy, first trimester
[O23.92] Unspecified genitourinary tract infection in pregnancy, second trimester
[O23.93] Unspecified genitourinary tract infection in pregnancy, third trimester
[O24.011] Pre-existing type 1 diabetes mellitus, in pregnancy, first trimester
[O24.012] Pre-existing type 1 diabetes mellitus, in pregnancy, second trimester
[O24.013] Pre-existing type 1 diabetes mellitus, in pregnancy, third trimester
[O24.019] Pre-existing type 1 diabetes mellitus, in pregnancy, unspecified trimester
[O24.111] Pre-existing type 2 diabetes mellitus, in pregnancy, first trimester
[O24.112] Pre-existing type 2 diabetes mellitus, in pregnancy, second trimester
[O24.113] Pre-existing type 2 diabetes mellitus, in pregnancy, third trimester
[O24.119] Pre-existing type 2 diabetes mellitus, in pregnancy, unspecified trimester

© 2024 Winterstein AG et al. *JAMA Network Open*.

[O24.311] Unspecified pre-existing diabetes mellitus in pregnancy, first trimester
[O24.312] Unspecified pre-existing diabetes mellitus in pregnancy, second trimester
[O24.313] Unspecified pre-existing diabetes mellitus in pregnancy, third trimester
[O24.319] Unspecified pre-existing diabetes mellitus in pregnancy, unspecified trimester
[O24.410] Gestational diabetes mellitus in pregnancy, diet controlled
[O24.414] Gestational diabetes mellitus in pregnancy, insulin controlled
[O24.415] Gestational diabetes mellitus in pregnancy, controlled by oral hypoglycemic drugs
[O24.419] Gestational diabetes mellitus in pregnancy, unspecified control
[O24.811] Other pre-existing diabetes mellitus in pregnancy, first trimester
[O24.812] Other pre-existing diabetes mellitus in pregnancy, second trimester
[O24.813] Other pre-existing diabetes mellitus in pregnancy, third trimester
[O24.819] Other pre-existing diabetes mellitus in pregnancy, unspecified trimester
[O24.911] Unspecified diabetes mellitus in pregnancy, first trimester
[O24.912] Unspecified diabetes mellitus in pregnancy, second trimester
[O24.913] Unspecified diabetes mellitus in pregnancy, third trimester
[O24.919] Unspecified diabetes mellitus in pregnancy, unspecified trimester
[O25.10] Malnutrition in pregnancy, unspecified trimester
[O25.11] Malnutrition in pregnancy, first trimester
[O25.12] Malnutrition in pregnancy, second trimester
[O25.13] Malnutrition in pregnancy, third trimester
[O26.00] Excessive weight gain in pregnancy, unspecified trimester
[O26.01] Excessive weight gain in pregnancy, first trimester
[O26.02] Excessive weight gain in pregnancy, second trimester
[O26.03] Excessive weight gain in pregnancy, third trimester
[O26.10] Low weight gain in pregnancy, unspecified trimester
[O26.11] Low weight gain in pregnancy, first trimester
[O26.12] Low weight gain in pregnancy, second trimester
[O26.13] Low weight gain in pregnancy, third trimester
[O26.20] Pregnancy care for patient with recurrent pregnancy loss, unspecified trimester
[O26.21] Pregnancy care for patient with recurrent pregnancy loss, first trimester
[O26.22] Pregnancy care for patient with recurrent pregnancy loss, second trimester
[O26.23] Pregnancy care for patient with recurrent pregnancy loss, third trimester
[O26.30] Retained intrauterine contraceptive device in pregnancy, unspecified trimester
[O26.31] Retained intrauterine contraceptive device in pregnancy, first trimester
[O26.32] Retained intrauterine contraceptive device in pregnancy, second trimester
[O26.33] Retained intrauterine contraceptive device in pregnancy, third trimester

© 2024 Winterstein AG et al. *JAMA Network Open*.

[O26.40] Herpes gestationis, unspecified trimester
[O26.41] Herpes gestationis, first trimester
[O26.42] Herpes gestationis, second trimester
[O26.43] Herpes gestationis, third trimester
[O26.50] Maternal hypotension syndrome, unspecified trimester
[O26.51] Maternal hypotension syndrome, first trimester
[O26.52] Maternal hypotension syndrome, second trimester
[O26.53] Maternal hypotension syndrome, third trimester
[O26.611] Liver and biliary tract disorders in pregnancy, first trimester
[O26.612] Liver and biliary tract disorders in pregnancy, second trimester
[O26.613] Liver and biliary tract disorders in pregnancy, third trimester
[O26.619] Liver and biliary tract disorders in pregnancy, unspecified trimester
[O26.711] Subluxation of symphysis (pubis) in pregnancy, first trimester
[O26.712] Subluxation of symphysis (pubis) in pregnancy, second trimester
[O26.713] Subluxation of symphysis (pubis) in pregnancy, third trimester
[O26.719] Subluxation of symphysis (pubis) in pregnancy, unspecified trimester
[O26.811] Pregnancy related exhaustion and fatigue, first trimester
[O26.812] Pregnancy related exhaustion and fatigue, second trimester
[O26.813] Pregnancy related exhaustion and fatigue, third trimester
[O26.819] Pregnancy related exhaustion and fatigue, unspecified trimester
[O26.821] Pregnancy related peripheral neuritis, first trimester
[O26.822] Pregnancy related peripheral neuritis, second trimester
[O26.823] Pregnancy related peripheral neuritis, third trimester
[O26.829] Pregnancy related peripheral neuritis, unspecified trimester
[O26.831] Pregnancy related renal disease, first trimester
[O26.832] Pregnancy related renal disease, second trimester
[O26.833] Pregnancy related renal disease, third trimester
[O26.839] Pregnancy related renal disease, unspecified trimester
[O26.841] Uterine size-date discrepancy, first trimester
[O26.842] Uterine size-date discrepancy, second trimester
[O26.843] Uterine size-date discrepancy, third trimester
[O26.849] Uterine size-date discrepancy, unspecified trimester
[O26.851] Spotting complicating pregnancy, first trimester
[O26.852] Spotting complicating pregnancy, second trimester
[O26.853] Spotting complicating pregnancy, third trimester
[O26.859] Spotting complicating pregnancy, unspecified trimester

© 2024 Winterstein AG et al. *JAMA Network Open*.

[O26.86] Pruritic urticarial papules and plaques of pregnancy (PUPPP)
[O26.872] Cervical shortening, second trimester
[O26.873] Cervical shortening, third trimester
[O26.879] Cervical shortening, unspecified trimester
[O26.891] Other specified pregnancy related conditions, first trimester
[O26.892] Other specified pregnancy related conditions, second trimester
[O26.893] Other specified pregnancy related conditions, third trimester
[O26.899] Other specified pregnancy related conditions, unspecified trimester
[O26.90] Pregnancy related conditions, unspecified, unspecified trimester
[O26.91] Pregnancy related conditions, unspecified, first trimester
[O26.92] Pregnancy related conditions, unspecified, second trimester
[O26.93] Pregnancy related conditions, unspecified, third trimester
[O28.0] Abnormal hematological finding on antenatal screening of mother
[O28.1] Abnormal biochemical finding on antenatal screening of mother
[O28.2] Abnormal cytological finding on antenatal screening of mother
[O28.3] Abnormal ultrasonic finding on antenatal screening of mother
[O28.4] Abnormal radiological finding on antenatal screening of mother
[O28.5] Abnormal chromosomal and genetic finding on antenatal screening of mother
[O28.8] Other abnormal findings on antenatal screening of mother
[O28.9] Unspecified abnormal findings on antenatal screening of mother
[O29.011] Aspiration pneumonitis due to anesthesia during pregnancy, first trimester
[O29.012] Aspiration pneumonitis due to anesthesia during pregnancy, second trimester
[O29.013] Aspiration pneumonitis due to anesthesia during pregnancy, third trimester
[O29.019] Aspiration pneumonitis due to anesthesia during pregnancy, unspecified trimester
[O29.021] Pressure collapse of lung due to anesthesia during pregnancy, first trimester
[O29.022] Pressure collapse of lung due to anesthesia during pregnancy, second trimester
[O29.023] Pressure collapse of lung due to anesthesia during pregnancy, third trimester
[O29.029] Pressure collapse of lung due to anesthesia during pregnancy, unspecified trimester
[O29.091] Other pulmonary complications of anesthesia during pregnancy, first trimester
[O29.092] Other pulmonary complications of anesthesia during pregnancy, second trimester
[O29.093] Other pulmonary complications of anesthesia during pregnancy, third trimester
[O29.099] Other pulmonary complications of anesthesia during pregnancy, unspecified trimester
[O29.111] Cardiac arrest due to anesthesia during pregnancy, first trimester
[O29.112] Cardiac arrest due to anesthesia during pregnancy, second trimester
[O29.113] Cardiac arrest due to anesthesia during pregnancy, third trimester
[O29.119] Cardiac arrest due to anesthesia during pregnancy, unspecified trimester

© 2024 Winterstein AG et al. *JAMA Network Open*.

[O29.121] Cardiac failure due to anesthesia during pregnancy, first trimester
[O29.122] Cardiac failure due to anesthesia during pregnancy, second trimester
[O29.123] Cardiac failure due to anesthesia during pregnancy, third trimester
[O29.129] Cardiac failure due to anesthesia during pregnancy, unspecified trimester
[O29.191] Other cardiac complications of anesthesia during pregnancy, first trimester
[O29.192] Other cardiac complications of anesthesia during pregnancy, second trimester
[O29.193] Other cardiac complications of anesthesia during pregnancy, third trimester
[O29.199] Other cardiac complications of anesthesia during pregnancy, unspecified trimester
[O29.211] Cerebral anoxia due to anesthesia during pregnancy, first trimester
[O29.212] Cerebral anoxia due to anesthesia during pregnancy, second trimester
[O29.213] Cerebral anoxia due to anesthesia during pregnancy, third trimester
[O29.219] Cerebral anoxia due to anesthesia during pregnancy, unspecified trimester
[O29.291] Other central nervous system complications of anesthesia during pregnancy, first trimester
[O29.292] Other central nervous system complications of anesthesia during pregnancy, second trimester
[O29.293] Other central nervous system complications of anesthesia during pregnancy, third trimester
[O29.299] Other central nervous system complications of anesthesia during pregnancy, unspecified trimester
[O29.3X1] Toxic reaction to local anesthesia during pregnancy, first trimester
[O29.3X2] Toxic reaction to local anesthesia during pregnancy, second trimester
[O29.3X3] Toxic reaction to local anesthesia during pregnancy, third trimester
[O29.3X9] Toxic reaction to local anesthesia during pregnancy, unspecified trimester
[O29.40] Spinal and epidural anesthesia induced headache during pregnancy, unspecified trimester
[O29.41] Spinal and epidural anesthesia induced headache during pregnancy, first trimester
[O29.42] Spinal and epidural anesthesia induced headache during pregnancy, second trimester
[O29.43] Spinal and epidural anesthesia induced headache during pregnancy, third trimester
[O29.5X1] Other complications of spinal and epidural anesthesia during pregnancy, first trimester
[O29.5X2] Other complications of spinal and epidural anesthesia during pregnancy, second trimester
[O29.5X3] Other complications of spinal and epidural anesthesia during pregnancy, third trimester
[O29.5X9] Other complications of spinal and epidural anesthesia during pregnancy, unspecified trimester
[O29.60] Failed or difficult intubation for anesthesia during pregnancy, unspecified trimester
[O29.61] Failed or difficult intubation for anesthesia during pregnancy, first trimester
[O29.62] Failed or difficult intubation for anesthesia during pregnancy, second trimester
[O29.63] Failed or difficult intubation for anesthesia during pregnancy, third trimester
[O29.8X1] Other complications of anesthesia during pregnancy, first trimester
[O29.8X2] Other complications of anesthesia during pregnancy, second trimester
[O29.8X3] Other complications of anesthesia during pregnancy, third trimester
[O29.8X9] Other complications of anesthesia during pregnancy, unspecified trimester

© 2024 Winterstein AG et al. *JAMA Network Open*.

[O29.90] Unspecified complication of anesthesia during pregnancy, unspecified trimester
[O29.91] Unspecified complication of anesthesia during pregnancy, first trimester
[O29.92] Unspecified complication of anesthesia during pregnancy, second trimester
[O29.93] Unspecified complication of anesthesia during pregnancy, third trimester
[O30.001] Twin pregnancy, unspecified number of placenta and unspecified number of amniotic sacs, first trimester
[O30.002] Twin pregnancy, unspecified number of placenta and unspecified number of amniotic sacs, second trimester
[O30.003] Twin pregnancy, unspecified number of placenta and unspecified number of amniotic sacs, third trimester
[O30.009] Twin pregnancy, unspecified number of placenta and unspecified number of amniotic sacs, unspecified trimester
[O30.011] Twin pregnancy, monochorionic/monoamniotic, first trimester
[O30.012] Twin pregnancy, monochorionic/monoamniotic, second trimester
[O30.013] Twin pregnancy, monochorionic/monoamniotic, third trimester
[O30.019] Twin pregnancy, monochorionic/monoamniotic, unspecified trimester
[O30.021] Conjoined twin pregnancy, first trimester
[O30.022] Conjoined twin pregnancy, second trimester
[O30.023] Conjoined twin pregnancy, third trimester
[O30.029] Conjoined twin pregnancy, unspecified trimester
[O30.031] Twin pregnancy, monochorionic/diamniotic, first trimester
[O30.032] Twin pregnancy, monochorionic/diamniotic, second trimester
[O30.033] Twin pregnancy, monochorionic/diamniotic, third trimester
[O30.039] Twin pregnancy, monochorionic/diamniotic, unspecified trimester
[O30.041] Twin pregnancy, dichorionic/diamniotic, first trimester
[O30.042] Twin pregnancy, dichorionic/diamniotic, second trimester
[O30.043] Twin pregnancy, dichorionic/diamniotic, third trimester
[O30.049] Twin pregnancy, dichorionic/diamniotic, unspecified trimester
[O30.091] Twin pregnancy, unable to determine number of placenta and number of amniotic sacs, first trimester
[O30.092] Twin pregnancy, unable to determine number of placenta and number of amniotic sacs, second trimester
[O30.093] Twin pregnancy, unable to determine number of placenta and number of amniotic sacs, third trimester
[O30.099] Twin pregnancy, unable to determine number of placenta and number of amniotic sacs, unspecified trimester
[O30.101] Triplet pregnancy, unspecified number of placenta and unspecified number of amniotic sacs, first trimester
[O30.102] Triplet pregnancy, unspecified number of placenta and unspecified number of amniotic sacs, second trimester
[O30.103] Triplet pregnancy, unspecified number of placenta and unspecified number of amniotic sacs, third trimester
[O30.109] Triplet pregnancy, unspecified number of placenta and unspecified number of amniotic sacs, unspecified trimester
[O30.111] Triplet pregnancy with two or more monochorionic fetuses, first trimester
[O30.112] Triplet pregnancy with two or more monochorionic fetuses, second trimester
[O30.113] Triplet pregnancy with two or more monochorionic fetuses, third trimester
[O30.119] Triplet pregnancy with two or more monochorionic fetuses, unspecified trimester

© 2024 Winterstein AG et al. *JAMA Network Open*.

[O30.121] Triplet pregnancy with two or more monoamniotic fetuses, first trimester
[O30.122] Triplet pregnancy with two or more monoamniotic fetuses, second trimester
[O30.123] Triplet pregnancy with two or more monoamniotic fetuses, third trimester
[O30.129] Triplet pregnancy with two or more monoamniotic fetuses, unspecified trimester
[O30.131] Triplet pregnancy, trichorionic/triamniotic, first trimester
[O30.132] Triplet pregnancy, trichorionic/triamniotic, second trimester
[O30.133] Triplet pregnancy, trichorionic/triamniotic, third trimester
[O30.139] Triplet pregnancy, trichorionic/triamniotic, unspecified trimester
[O30.191] Triplet pregnancy, unable to determine number of placenta and number of amniotic sacs, first trimester
[O30.192] Triplet pregnancy, unable to determine number of placenta and number of amniotic sacs, second trimester
[O30.193] Triplet pregnancy, unable to determine number of placenta and number of amniotic sacs, third trimester
[O30.199] Triplet pregnancy, unable to determine number of placenta and number of amniotic sacs, unspecified trimester
[O30.201] Quadruplet pregnancy, unspecified number of placenta and unspecified number of amniotic sacs, first trimester
[O30.202] Quadruplet pregnancy, unspecified number of placenta and unspecified number of amniotic sacs, second trimester
[O30.203] Quadruplet pregnancy, unspecified number of placenta and unspecified number of amniotic sacs, third trimester
[O30.209] Quadruplet pregnancy, unspecified number of placenta and unspecified number of amniotic sacs, unspecified trimester
[O30.211] Quadruplet pregnancy with two or more monochorionic fetuses, first trimester
[O30.212] Quadruplet pregnancy with two or more monochorionic fetuses, second trimester
[O30.213] Quadruplet pregnancy with two or more monochorionic fetuses, third trimester
[O30.219] Quadruplet pregnancy with two or more monochorionic fetuses, unspecified trimester
[O30.221] Quadruplet pregnancy with two or more monoamniotic fetuses, first trimester
[O30.222] Quadruplet pregnancy with two or more monoamniotic fetuses, second trimester
[O30.223] Quadruplet pregnancy with two or more monoamniotic fetuses, third trimester
[O30.229] Quadruplet pregnancy with two or more monoamniotic fetuses, unspecified trimester
[O30.231] Quadruplet pregnancy, quadrachorionic/quadra-amniotic, first trimester
[O30.232] Quadruplet pregnancy, quadrachorionic/quadra-amniotic, second trimester
[O30.233] Quadruplet pregnancy, quadrachorionic/quadra-amniotic, third trimester
[O30.239] Quadruplet pregnancy, quadrachorionic/quadra-amniotic, unspecified trimester
[O30.291] Quadruplet pregnancy, unable to determine number of placenta and number of amniotic sacs, first trimester
[O30.292] Quadruplet pregnancy, unable to determine number of placenta and number of amniotic sacs, second trimester
[O30.293] Quadruplet pregnancy, unable to determine number of placenta and number of amniotic sacs, third trimester
[O30.299] Quadruplet pregnancy, unable to determine number of placenta and number of amniotic sacs, unspecified trimester
[O30.801] Other specified multiple gestation, unspecified number of placenta and unspecified number of amniotic sacs, first trimester
[O30.802] Other specified multiple gestation, unspecified number of placenta and unspecified number of amniotic sacs, second trimester

© 2024 Winterstein AG et al. *JAMA Network Open*.

[O30.803] Other specified multiple gestation, unspecified number of placenta and unspecified number of amniotic sacs, third trimester
[O30.809] Other specified multiple gestation, unspecified number of placenta and unspecified number of amniotic sacs, unspecified trimester
[O30.811] Other specified multiple gestation with two or more monochorionic fetuses, first trimester
[O30.812] Other specified multiple gestation with two or more monochorionic fetuses, second trimester
[O30.813] Other specified multiple gestation with two or more monochorionic fetuses, third trimester
[O30.819] Other specified multiple gestation with two or more monochorionic fetuses, unspecified trimester
[O30.821] Other specified multiple gestation with two or more monoamniotic fetuses, first trimester
[O30.822] Other specified multiple gestation with two or more monoamniotic fetuses, second trimester
[O30.823] Other specified multiple gestation with two or more monoamniotic fetuses, third trimester
[O30.829] Other specified multiple gestation with two or more monoamniotic fetuses, unspecified trimester
[O30.831] Other specified multiple gestation, number of chorions and amnions are both equal to the number of fetuses, first trimester
[O30.832] Other specified multiple gestation, number of chorions and amnions are both equal to the number of fetuses, second trimester
[O30.833] Other specified multiple gestation, number of chorions and amnions are both equal to the number of fetuses, third trimester
[O30.839] Other specified multiple gestation, number of chorions and amnions are both equal to the number of fetuses, unspecified trimester
[O30.891] Other specified multiple gestation, unable to determine number of placenta and number of amniotic sacs, first trimester
[O30.892] Other specified multiple gestation, unable to determine number of placenta and number of amniotic sacs, second trimester
[O30.893] Other specified multiple gestation, unable to determine number of placenta and number of amniotic sacs, third trimester
[O30.899] Other specified multiple gestation, unable to determine number of placenta and number of amniotic sacs, unspecified trimester
[O30.90] Multiple gestation, unspecified, unspecified trimester
[O30.91] Multiple gestation, unspecified, first trimester
[O30.92] Multiple gestation, unspecified, second trimester
[O30.93] Multiple gestation, unspecified, third trimester
[O31.00X0] Papyraceous fetus, unspecified trimester, not applicable or unspecified
[O31.00X1] Papyraceous fetus, unspecified trimester, fetus 1
[O31.00X2] Papyraceous fetus, unspecified trimester, fetus 2
[O31.00X3] Papyraceous fetus, unspecified trimester, fetus 3
[O31.00X4] Papyraceous fetus, unspecified trimester, fetus 4
[O31.00X5] Papyraceous fetus, unspecified trimester, fetus 5
[O31.00X9] Papyraceous fetus, unspecified trimester, other fetus
[O31.01X0] Papyraceous fetus, first trimester, not applicable or unspecified

© 2024 Winterstein AG et al. *JAMA Network Open*.

[O31.01X1] Papyraceous fetus, first trimester, fetus 1
[O31.01X2] Papyraceous fetus, first trimester, fetus 2
[O31.01X3] Papyraceous fetus, first trimester, fetus 3
[O31.01X4] Papyraceous fetus, first trimester, fetus 4
[O31.01X5] Papyraceous fetus, first trimester, fetus 5
[O31.01X9] Papyraceous fetus, first trimester, other fetus
[O31.02X0] Papyraceous fetus, second trimester, not applicable or unspecified
[O31.02X1] Papyraceous fetus, second trimester, fetus 1
[O31.02X2] Papyraceous fetus, second trimester, fetus 2
[O31.02X3] Papyraceous fetus, second trimester, fetus 3
[O31.02X4] Papyraceous fetus, second trimester, fetus 4
[O31.02X5] Papyraceous fetus, second trimester, fetus 5
[O31.02X9] Papyraceous fetus, second trimester, other fetus
[O31.03X0] Papyraceous fetus, third trimester, not applicable or unspecified
[O31.03X1] Papyraceous fetus, third trimester, fetus 1
[O31.03X2] Papyraceous fetus, third trimester, fetus 2
[O31.03X3] Papyraceous fetus, third trimester, fetus 3
[O31.03X4] Papyraceous fetus, third trimester, fetus 4
[O31.03X5] Papyraceous fetus, third trimester, fetus 5
[O31.03X9] Papyraceous fetus, third trimester, other fetus
[O31.10X0] Continuing pregnancy after spontaneous abortion of one fetus or more, unspecified trimester, not applicable or unspecified
[O31.10X1] Continuing pregnancy after spontaneous abortion of one fetus or more, unspecified trimester, fetus 1
[O31.10X2] Continuing pregnancy after spontaneous abortion of one fetus or more, unspecified trimester, fetus 2
[O31.10X3] Continuing pregnancy after spontaneous abortion of one fetus or more, unspecified trimester, fetus 3
[O31.10X4] Continuing pregnancy after spontaneous abortion of one fetus or more, unspecified trimester, fetus 4
[O31.10X5] Continuing pregnancy after spontaneous abortion of one fetus or more, unspecified trimester, fetus 5
[O31.10X9] Continuing pregnancy after spontaneous abortion of one fetus or more, unspecified trimester, other fetus
[O31.11X0] Continuing pregnancy after spontaneous abortion of one fetus or more, first trimester, not applicable or unspecified
[O31.11X1] Continuing pregnancy after spontaneous abortion of one fetus or more, first trimester, fetus 1
[O31.11X2] Continuing pregnancy after spontaneous abortion of one fetus or more, first trimester, fetus 2
[O31.11X3] Continuing pregnancy after spontaneous abortion of one fetus or more, first trimester, fetus 3
[O31.11X4] Continuing pregnancy after spontaneous abortion of one fetus or more, first trimester, fetus 4
[O31.11X5] Continuing pregnancy after spontaneous abortion of one fetus or more, first trimester, fetus 5
[O31.11X9] Continuing pregnancy after spontaneous abortion of one fetus or more, first trimester, other fetus
[O31.12X0] Continuing pregnancy after spontaneous abortion of one fetus or more, second trimester, not applicable or unspecified

© 2024 Winterstein AG et al. *JAMA Network Open*.

[O31.12X1] Continuing pregnancy after spontaneous abortion of one fetus or more, second trimester, fetus 1
[O31.12X2] Continuing pregnancy after spontaneous abortion of one fetus or more, second trimester, fetus 2
[O31.12X3] Continuing pregnancy after spontaneous abortion of one fetus or more, second trimester, fetus 3
[O31.12X4] Continuing pregnancy after spontaneous abortion of one fetus or more, second trimester, fetus 4
[O31.12X5] Continuing pregnancy after spontaneous abortion of one fetus or more, second trimester, fetus 5
[O31.12X9] Continuing pregnancy after spontaneous abortion of one fetus or more, second trimester, other fetus
[O31.13X0] Continuing pregnancy after spontaneous abortion of one fetus or more, third trimester, not applicable or unspecified
[O31.13X1] Continuing pregnancy after spontaneous abortion of one fetus or more, third trimester, fetus 1
[O31.13X2] Continuing pregnancy after spontaneous abortion of one fetus or more, third trimester, fetus 2
[O31.13X3] Continuing pregnancy after spontaneous abortion of one fetus or more, third trimester, fetus 3
[O31.13X4] Continuing pregnancy after spontaneous abortion of one fetus or more, third trimester, fetus 4
[O31.13X5] Continuing pregnancy after spontaneous abortion of one fetus or more, third trimester, fetus 5
[O31.13X9] Continuing pregnancy after spontaneous abortion of one fetus or more, third trimester, other fetus
[O31.20X0] Continuing pregnancy after intrauterine death of one fetus or more, unspecified trimester, not applicable or unspecified
[O31.20X1] Continuing pregnancy after intrauterine death of one fetus or more, unspecified trimester, fetus 1
[O31.20X2] Continuing pregnancy after intrauterine death of one fetus or more, unspecified trimester, fetus 2
[O31.20X3] Continuing pregnancy after intrauterine death of one fetus or more, unspecified trimester, fetus 3
[O31.20X4] Continuing pregnancy after intrauterine death of one fetus or more, unspecified trimester, fetus 4
[O31.20X5] Continuing pregnancy after intrauterine death of one fetus or more, unspecified trimester, fetus 5
[O31.20X9] Continuing pregnancy after intrauterine death of one fetus or more, unspecified trimester, other fetus
[O31.21X0] Continuing pregnancy after intrauterine death of one fetus or more, first trimester, not applicable or unspecified
[O31.21X1] Continuing pregnancy after intrauterine death of one fetus or more, first trimester, fetus 1
[O31.21X2] Continuing pregnancy after intrauterine death of one fetus or more, first trimester, fetus 2
[O31.21X3] Continuing pregnancy after intrauterine death of one fetus or more, first trimester, fetus 3
[O31.21X4] Continuing pregnancy after intrauterine death of one fetus or more, first trimester, fetus 4
[O31.21X5] Continuing pregnancy after intrauterine death of one fetus or more, first trimester, fetus 5
[O31.21X9] Continuing pregnancy after intrauterine death of one fetus or more, first trimester, other fetus
[O31.22X0] Continuing pregnancy after intrauterine death of one fetus or more, second trimester, not applicable or unspecified
[O31.22X1] Continuing pregnancy after intrauterine death of one fetus or more, second trimester, fetus 1
[O31.22X2] Continuing pregnancy after intrauterine death of one fetus or more, second trimester, fetus 2
[O31.22X3] Continuing pregnancy after intrauterine death of one fetus or more, second trimester, fetus 3
[O31.22X4] Continuing pregnancy after intrauterine death of one fetus or more, second trimester, fetus 4
[O31.22X5] Continuing pregnancy after intrauterine death of one fetus or more, second trimester, fetus 5
[O31.22X9] Continuing pregnancy after intrauterine death of one fetus or more, second trimester, other fetus
[O31.23X0] Continuing pregnancy after intrauterine death of one fetus or more, third trimester, not applicable or unspecified
[O31.23X1] Continuing pregnancy after intrauterine death of one fetus or more, third trimester, fetus 1

© 2024 Winterstein AG et al. *JAMA Network Open*.

[O31.23X2] Continuing pregnancy after intrauterine death of one fetus or more, third trimester, fetus 2
[O31.23X3] Continuing pregnancy after intrauterine death of one fetus or more, third trimester, fetus 3
[O31.23X4] Continuing pregnancy after intrauterine death of one fetus or more, third trimester, fetus 4
[O31.23X5] Continuing pregnancy after intrauterine death of one fetus or more, third trimester, fetus 5
[O31.23X9] Continuing pregnancy after intrauterine death of one fetus or more, third trimester, other fetus
[O31.30X0] Continuing pregnancy after elective fetal reduction of one fetus or more, unspecified trimester, not applicable or unspecified
[O31.30X1] Continuing pregnancy after elective fetal reduction of one fetus or more, unspecified trimester, fetus 1
[O31.30X2] Continuing pregnancy after elective fetal reduction of one fetus or more, unspecified trimester, fetus 2
[O31.30X3] Continuing pregnancy after elective fetal reduction of one fetus or more, unspecified trimester, fetus 3
[O31.30X4] Continuing pregnancy after elective fetal reduction of one fetus or more, unspecified trimester, fetus 4
[O31.30X5] Continuing pregnancy after elective fetal reduction of one fetus or more, unspecified trimester, fetus 5
[O31.30X9] Continuing pregnancy after elective fetal reduction of one fetus or more, unspecified trimester, other fetus
[O31.31X0] Continuing pregnancy after elective fetal reduction of one fetus or more, first trimester, not applicable or unspecified
[O31.31X1] Continuing pregnancy after elective fetal reduction of one fetus or more, first trimester, fetus 1
[O31.31X2] Continuing pregnancy after elective fetal reduction of one fetus or more, first trimester, fetus 2
[O31.31X3] Continuing pregnancy after elective fetal reduction of one fetus or more, first trimester, fetus 3
[O31.31X4] Continuing pregnancy after elective fetal reduction of one fetus or more, first trimester, fetus 4
[O31.31X5] Continuing pregnancy after elective fetal reduction of one fetus or more, first trimester, fetus 5
[O31.31X9] Continuing pregnancy after elective fetal reduction of one fetus or more, first trimester, other fetus
[O31.32X0] Continuing pregnancy after elective fetal reduction of one fetus or more, second trimester, not applicable or unspecified
[O31.32X1] Continuing pregnancy after elective fetal reduction of one fetus or more, second trimester, fetus 1
[O31.32X2] Continuing pregnancy after elective fetal reduction of one fetus or more, second trimester, fetus 2
[O31.32X3] Continuing pregnancy after elective fetal reduction of one fetus or more, second trimester, fetus 3
[O31.32X4] Continuing pregnancy after elective fetal reduction of one fetus or more, second trimester, fetus 4
[O31.32X5] Continuing pregnancy after elective fetal reduction of one fetus or more, second trimester, fetus 5
[O31.32X9] Continuing pregnancy after elective fetal reduction of one fetus or more, second trimester, other fetus
[O31.33X0] Continuing pregnancy after elective fetal reduction of one fetus or more, third trimester, not applicable or unspecified
[O31.33X1] Continuing pregnancy after elective fetal reduction of one fetus or more, third trimester, fetus 1
[O31.33X2] Continuing pregnancy after elective fetal reduction of one fetus or more, third trimester, fetus 2
[O31.33X3] Continuing pregnancy after elective fetal reduction of one fetus or more, third trimester, fetus 3
[O31.33X4] Continuing pregnancy after elective fetal reduction of one fetus or more, third trimester, fetus 4
[O31.33X5] Continuing pregnancy after elective fetal reduction of one fetus or more, third trimester, fetus 5
[O31.33X9] Continuing pregnancy after elective fetal reduction of one fetus or more, third trimester, other fetus
[O31.8X10] Other complications specific to multiple gestation, first trimester, not applicable or unspecified
[O31.8X11] Other complications specific to multiple gestation, first trimester, fetus 1

© 2024 Winterstein AG et al. *JAMA Network Open*.

[O31.8X12] Other complications specific to multiple gestation, first trimester, fetus 2
[O31.8X13] Other complications specific to multiple gestation, first trimester, fetus 3
[O31.8X14] Other complications specific to multiple gestation, first trimester, fetus 4
[O31.8X15] Other complications specific to multiple gestation, first trimester, fetus 5
[O31.8X19] Other complications specific to multiple gestation, first trimester, other fetus
[O31.8X20] Other complications specific to multiple gestation, second trimester, not applicable or unspecified
[O31.8X21] Other complications specific to multiple gestation, second trimester, fetus 1
[O31.8X22] Other complications specific to multiple gestation, second trimester, fetus 2
[O31.8X23] Other complications specific to multiple gestation, second trimester, fetus 3
[O31.8X24] Other complications specific to multiple gestation, second trimester, fetus 4
[O31.8X25] Other complications specific to multiple gestation, second trimester, fetus 5
[O31.8X29] Other complications specific to multiple gestation, second trimester, other fetus
[O31.8X30] Other complications specific to multiple gestation, third trimester, not applicable or unspecified
[O31.8X31] Other complications specific to multiple gestation, third trimester, fetus 1
[O31.8X32] Other complications specific to multiple gestation, third trimester, fetus 2
[O31.8X33] Other complications specific to multiple gestation, third trimester, fetus 3
[O31.8X34] Other complications specific to multiple gestation, third trimester, fetus 4
[O31.8X35] Other complications specific to multiple gestation, third trimester, fetus 5
[O31.8X39] Other complications specific to multiple gestation, third trimester, other fetus
[O31.8X90] Other complications specific to multiple gestation, unspecified trimester, not applicable or unspecified
[O31.8X91] Other complications specific to multiple gestation, unspecified trimester, fetus 1
[O31.8X92] Other complications specific to multiple gestation, unspecified trimester, fetus 2
[O31.8X93] Other complications specific to multiple gestation, unspecified trimester, fetus 3
[O31.8X94] Other complications specific to multiple gestation, unspecified trimester, fetus 4
[O31.8X95] Other complications specific to multiple gestation, unspecified trimester, fetus 5
[O31.8X99] Other complications specific to multiple gestation, unspecified trimester, other fetus
[O32.0XX0] Maternal care for unstable lie, not applicable or unspecified
[O32.0XX1] Maternal care for unstable lie, fetus 1
[O32.0XX2] Maternal care for unstable lie, fetus 2
[O32.0XX3] Maternal care for unstable lie, fetus 3
[O32.0XX4] Maternal care for unstable lie, fetus 4
[O32.0XX5] Maternal care for unstable lie, fetus 5
[O32.0XX9] Maternal care for unstable lie, other fetus
[O32.1XX0] Maternal care for breech presentation, not applicable or unspecified
[O32.1XX1] Maternal care for breech presentation, fetus 1
[O32.1XX2] Maternal care for breech presentation, fetus 2

© 2024 Winterstein AG et al. *JAMA Network Open*.

[O32.1XX3] Maternal care for breech presentation, fetus 3
[O32.1XX4] Maternal care for breech presentation, fetus 4
[O32.1XX5] Maternal care for breech presentation, fetus 5
[O32.1XX9] Maternal care for breech presentation, other fetus
[O32.2XX0] Maternal care for transverse and oblique lie, not applicable or unspecified
[O32.2XX1] Maternal care for transverse and oblique lie, fetus 1
[O32.2XX2] Maternal care for transverse and oblique lie, fetus 2
[O32.2XX3] Maternal care for transverse and oblique lie, fetus 3
[O32.2XX4] Maternal care for transverse and oblique lie, fetus 4
[O32.2XX5] Maternal care for transverse and oblique lie, fetus 5
[O32.2XX9] Maternal care for transverse and oblique lie, other fetus
[O32.3XX0] Maternal care for face, brow and chin presentation, not applicable or unspecified
[O32.3XX1] Maternal care for face, brow and chin presentation, fetus 1
[O32.3XX2] Maternal care for face, brow and chin presentation, fetus 2
[O32.3XX3] Maternal care for face, brow and chin presentation, fetus 3
[O32.3XX4] Maternal care for face, brow and chin presentation, fetus 4
[O32.3XX5] Maternal care for face, brow and chin presentation, fetus 5
[O32.3XX9] Maternal care for face, brow and chin presentation, other fetus
[O32.4XX0] Maternal care for high head at term, not applicable or unspecified
[O32.4XX1] Maternal care for high head at term, fetus 1
[O32.4XX2] Maternal care for high head at term, fetus 2
[O32.4XX3] Maternal care for high head at term, fetus 3
[O32.4XX4] Maternal care for high head at term, fetus 4
[O32.4XX5] Maternal care for high head at term, fetus 5
[O32.4XX9] Maternal care for high head at term, other fetus
[O32.6XX0] Maternal care for compound presentation, not applicable or unspecified
[O32.6XX1] Maternal care for compound presentation, fetus 1
[O32.6XX2] Maternal care for compound presentation, fetus 2
[O32.6XX3] Maternal care for compound presentation, fetus 3
[O32.6XX4] Maternal care for compound presentation, fetus 4
[O32.6XX5] Maternal care for compound presentation, fetus 5
[O32.6XX9] Maternal care for compound presentation, other fetus
[O32.8XX0] Maternal care for other malpresentation of fetus, not applicable or unspecified
[O32.8XX1] Maternal care for other malpresentation of fetus, fetus 1
[O32.8XX2] Maternal care for other malpresentation of fetus, fetus 2
[O32.8XX3] Maternal care for other malpresentation of fetus, fetus 3

© 2024 Winterstein AG et al. *JAMA Network Open*.

[O32.8XX4] Maternal care for other malpresentation of fetus, fetus 4
[O32.8XX5] Maternal care for other malpresentation of fetus, fetus 5
[O32.8XX9] Maternal care for other malpresentation of fetus, other fetus
[O32.9XX0] Maternal care for malpresentation of fetus, unspecified, not applicable or unspecified
[O32.9XX1] Maternal care for malpresentation of fetus, unspecified, fetus 1
[O32.9XX2] Maternal care for malpresentation of fetus, unspecified, fetus 2
[O32.9XX3] Maternal care for malpresentation of fetus, unspecified, fetus 3
[O32.9XX4] Maternal care for malpresentation of fetus, unspecified, fetus 4
[O32.9XX5] Maternal care for malpresentation of fetus, unspecified, fetus 5
[O32.9XX9] Maternal care for malpresentation of fetus, unspecified, other fetus
[O33.0] Maternal care for disproportion due to deformity of maternal pelvic bones
[O33.1] Maternal care for disproportion due to generally contracted pelvis
[O33.2] Maternal care for disproportion due to inlet contraction of pelvis
[O33.3XX0] Maternal care for disproportion due to outlet contraction of pelvis, not applicable or unspecified
[O33.3XX1] Maternal care for disproportion due to outlet contraction of pelvis, fetus 1
[O33.3XX2] Maternal care for disproportion due to outlet contraction of pelvis, fetus 2
[O33.3XX3] Maternal care for disproportion due to outlet contraction of pelvis, fetus 3
[O33.3XX4] Maternal care for disproportion due to outlet contraction of pelvis, fetus 4
[O33.3XX5] Maternal care for disproportion due to outlet contraction of pelvis, fetus 5
[O33.3XX9] Maternal care for disproportion due to outlet contraction of pelvis, other fetus
[O33.4XX0] Maternal care for disproportion of mixed maternal and fetal origin, not applicable or unspecified
[O33.4XX1] Maternal care for disproportion of mixed maternal and fetal origin, fetus 1
[O33.4XX2] Maternal care for disproportion of mixed maternal and fetal origin, fetus 2
[O33.4XX3] Maternal care for disproportion of mixed maternal and fetal origin, fetus 3
[O33.4XX4] Maternal care for disproportion of mixed maternal and fetal origin, fetus 4
[O33.4XX5] Maternal care for disproportion of mixed maternal and fetal origin, fetus 5
[O33.4XX9] Maternal care for disproportion of mixed maternal and fetal origin, other fetus
[O33.5XX0] Maternal care for disproportion due to unusually large fetus, not applicable or unspecified
[O33.5XX1] Maternal care for disproportion due to unusually large fetus, fetus 1
[O33.5XX2] Maternal care for disproportion due to unusually large fetus, fetus 2
[O33.5XX3] Maternal care for disproportion due to unusually large fetus, fetus 3
[O33.5XX4] Maternal care for disproportion due to unusually large fetus, fetus 4
[O33.5XX5] Maternal care for disproportion due to unusually large fetus, fetus 5
[O33.5XX9] Maternal care for disproportion due to unusually large fetus, other fetus
[O33.6XX0] Maternal care for disproportion due to hydrocephalic fetus, not applicable or unspecified
[O33.6XX1] Maternal care for disproportion due to hydrocephalic fetus, fetus 1

© 2024 Winterstein AG et al. *JAMA Network Open*.

[O33.6XX2] Maternal care for disproportion due to hydrocephalic fetus, fetus 2
[O33.6XX3] Maternal care for disproportion due to hydrocephalic fetus, fetus 3
[O33.6XX4] Maternal care for disproportion due to hydrocephalic fetus, fetus 4
[O33.6XX5] Maternal care for disproportion due to hydrocephalic fetus, fetus 5
[O33.6XX9] Maternal care for disproportion due to hydrocephalic fetus, other fetus
[O33.7] Maternal care for disproportion due to other fetal deformities
[O33.7XX0] Maternal care for disproportion due to other fetal deformities, not applicable or unspecified
[O33.7XX1] Maternal care for disproportion due to other fetal deformities, fetus 1
[O33.7XX2] Maternal care for disproportion due to other fetal deformities, fetus 2
[O33.7XX3] Maternal care for disproportion due to other fetal deformities, fetus 3
[O33.7XX4] Maternal care for disproportion due to other fetal deformities, fetus 4
[O33.7XX5] Maternal care for disproportion due to other fetal deformities, fetus 5
[O33.7XX9] Maternal care for disproportion due to other fetal deformities, other fetus
[O33.8] Maternal care for disproportion of other origin
[O33.9] Maternal care for disproportion, unspecified
[O34.00] Maternal care for unspecified congenital malformation of uterus, unspecified trimester
[O34.01] Maternal care for unspecified congenital malformation of uterus, first trimester
[O34.02] Maternal care for unspecified congenital malformation of uterus, second trimester
[O34.03] Maternal care for unspecified congenital malformation of uterus, third trimester
[O34.10] Maternal care for benign tumor of corpus uteri, unspecified trimester
[O34.11] Maternal care for benign tumor of corpus uteri, first trimester
[O34.12] Maternal care for benign tumor of corpus uteri, second trimester
[O34.13] Maternal care for benign tumor of corpus uteri, third trimester
[O34.21] Maternal care for scar from previous cesarean delivery
[O34.211] Maternal care for low transverse scar from previous cesarean delivery
[O34.212] Maternal care for vertical scar from previous cesarean delivery
[O34.218] Maternal care for other type scar from previous cesarean delivery
[O34.219] Maternal care for unspecified type scar from previous cesarean delivery
[O34.22] Maternal care for cesarean scar defect (isthmocele)
[O34.29] Maternal care due to uterine scar from other previous surgery
[O34.30] Maternal care for cervical incompetence, unspecified trimester
[O34.31] Maternal care for cervical incompetence, first trimester
[O34.32] Maternal care for cervical incompetence, second trimester
[O34.33] Maternal care for cervical incompetence, third trimester
[O34.40] Maternal care for other abnormalities of cervix, unspecified trimester
[O34.41] Maternal care for other abnormalities of cervix, first trimester

© 2024 Winterstein AG et al. *JAMA Network Open*.

[O34.42] Maternal care for other abnormalities of cervix, second trimester
[O34.43] Maternal care for other abnormalities of cervix, third trimester
[O34.511] Maternal care for incarceration of gravid uterus, first trimester
[O34.512] Maternal care for incarceration of gravid uterus, second trimester
[O34.513] Maternal care for incarceration of gravid uterus, third trimester
[O34.519] Maternal care for incarceration of gravid uterus, unspecified trimester
[O34.521] Maternal care for prolapse of gravid uterus, first trimester
[O34.522] Maternal care for prolapse of gravid uterus, second trimester
[O34.523] Maternal care for prolapse of gravid uterus, third trimester
[O34.529] Maternal care for prolapse of gravid uterus, unspecified trimester
[O34.531] Maternal care for retroversion of gravid uterus, first trimester
[O34.532] Maternal care for retroversion of gravid uterus, second trimester
[O34.533] Maternal care for retroversion of gravid uterus, third trimester
[O34.539] Maternal care for retroversion of gravid uterus, unspecified trimester
[O34.591] Maternal care for other abnormalities of gravid uterus, first trimester
[O34.592] Maternal care for other abnormalities of gravid uterus, second trimester
[O34.593] Maternal care for other abnormalities of gravid uterus, third trimester
[O34.599] Maternal care for other abnormalities of gravid uterus, unspecified trimester
[O34.60] Maternal care for abnormality of vagina, unspecified trimester
[O34.61] Maternal care for abnormality of vagina, first trimester
[O34.62] Maternal care for abnormality of vagina, second trimester
[O34.63] Maternal care for abnormality of vagina, third trimester
[O34.70] Maternal care for abnormality of vulva and perineum, unspecified trimester
[O34.71] Maternal care for abnormality of vulva and perineum, first trimester
[O34.72] Maternal care for abnormality of vulva and perineum, second trimester
[O34.73] Maternal care for abnormality of vulva and perineum, third trimester
[O34.80] Maternal care for other abnormalities of pelvic organs, unspecified trimester
[O34.81] Maternal care for other abnormalities of pelvic organs, first trimester
[O34.82] Maternal care for other abnormalities of pelvic organs, second trimester
[O34.83] Maternal care for other abnormalities of pelvic organs, third trimester
[O34.90] Maternal care for abnormality of pelvic organ, unspecified, unspecified trimester
[O34.91] Maternal care for abnormality of pelvic organ, unspecified, first trimester
[O34.92] Maternal care for abnormality of pelvic organ, unspecified, second trimester
[O34.93] Maternal care for abnormality of pelvic organ, unspecified, third trimester
[O35.0XX0] Maternal care for (suspected) central nervous system malformation in fetus, not applicable or unspecified
[O35.0XX1] Maternal care for (suspected) central nervous system malformation in fetus, fetus 1

© 2024 Winterstein AG et al. *JAMA Network Open*.

[O35.0XX2] Maternal care for (suspected) central nervous system malformation in fetus, fetus 2
[O35.0XX3] Maternal care for (suspected) central nervous system malformation in fetus, fetus 3
[O35.0XX4] Maternal care for (suspected) central nervous system malformation in fetus, fetus 4
[O35.0XX5] Maternal care for (suspected) central nervous system malformation in fetus, fetus 5
[O35.0XX9] Maternal care for (suspected) central nervous system malformation in fetus, other fetus
[O35.1XX0] Maternal care for (suspected) chromosomal abnormality in fetus, not applicable or unspecified
[O35.1XX1] Maternal care for (suspected) chromosomal abnormality in fetus, fetus 1
[O35.1XX2] Maternal care for (suspected) chromosomal abnormality in fetus, fetus 2
[O35.1XX3] Maternal care for (suspected) chromosomal abnormality in fetus, fetus 3
[O35.1XX4] Maternal care for (suspected) chromosomal abnormality in fetus, fetus 4
[O35.1XX5] Maternal care for (suspected) chromosomal abnormality in fetus, fetus 5
[O35.1XX9] Maternal care for (suspected) chromosomal abnormality in fetus, other fetus
[O35.2XX0] Maternal care for (suspected) hereditary disease in fetus, not applicable or unspecified
[O35.2XX1] Maternal care for (suspected) hereditary disease in fetus, fetus 1
[O35.2XX2] Maternal care for (suspected) hereditary disease in fetus, fetus 2
[O35.2XX3] Maternal care for (suspected) hereditary disease in fetus, fetus 3
[O35.2XX4] Maternal care for (suspected) hereditary disease in fetus, fetus 4
[O35.2XX5] Maternal care for (suspected) hereditary disease in fetus, fetus 5
[O35.2XX9] Maternal care for (suspected) hereditary disease in fetus, other fetus
[O35.3XX0] Maternal care for (suspected) damage to fetus from viral disease in mother, not applicable or unspecified
[O35.3XX1] Maternal care for (suspected) damage to fetus from viral disease in mother, fetus 1
[O35.3XX2] Maternal care for (suspected) damage to fetus from viral disease in mother, fetus 2
[O35.3XX3] Maternal care for (suspected) damage to fetus from viral disease in mother, fetus 3
[O35.3XX4] Maternal care for (suspected) damage to fetus from viral disease in mother, fetus 4
[O35.3XX5] Maternal care for (suspected) damage to fetus from viral disease in mother, fetus 5
[O35.3XX9] Maternal care for (suspected) damage to fetus from viral disease in mother, other fetus
[O35.4XX0] Maternal care for (suspected) damage to fetus from alcohol, not applicable or unspecified
[O35.4XX1] Maternal care for (suspected) damage to fetus from alcohol, fetus 1
[O35.4XX2] Maternal care for (suspected) damage to fetus from alcohol, fetus 2
[O35.4XX3] Maternal care for (suspected) damage to fetus from alcohol, fetus 3
[O35.4XX4] Maternal care for (suspected) damage to fetus from alcohol, fetus 4
[O35.4XX5] Maternal care for (suspected) damage to fetus from alcohol, fetus 5
[O35.4XX9] Maternal care for (suspected) damage to fetus from alcohol, other fetus
[O35.5XX0] Maternal care for (suspected) damage to fetus by drugs, not applicable or unspecified
[O35.5XX1] Maternal care for (suspected) damage to fetus by drugs, fetus 1
[O35.5XX2] Maternal care for (suspected) damage to fetus by drugs, fetus 2

© 2024 Winterstein AG et al. *JAMA Network Open*.

[O35.5XX3] Maternal care for (suspected) damage to fetus by drugs, fetus 3
[O35.5XX4] Maternal care for (suspected) damage to fetus by drugs, fetus 4
[O35.5XX5] Maternal care for (suspected) damage to fetus by drugs, fetus 5
[O35.5XX9] Maternal care for (suspected) damage to fetus by drugs, other fetus
[O35.6XX0] Maternal care for (suspected) damage to fetus by radiation, not applicable or unspecified
[O35.6XX1] Maternal care for (suspected) damage to fetus by radiation, fetus 1
[O35.6XX2] Maternal care for (suspected) damage to fetus by radiation, fetus 2
[O35.6XX3] Maternal care for (suspected) damage to fetus by radiation, fetus 3
[O35.6XX4] Maternal care for (suspected) damage to fetus by radiation, fetus 4
[O35.6XX5] Maternal care for (suspected) damage to fetus by radiation, fetus 5
[O35.6XX9] Maternal care for (suspected) damage to fetus by radiation, other fetus
[O35.7XX0] Maternal care for (suspected) damage to fetus by other medical procedures, not applicable or unspecified
[O35.7XX1] Maternal care for (suspected) damage to fetus by other medical procedures, fetus 1
[O35.7XX2] Maternal care for (suspected) damage to fetus by other medical procedures, fetus 2
[O35.7XX3] Maternal care for (suspected) damage to fetus by other medical procedures, fetus 3
[O35.7XX4] Maternal care for (suspected) damage to fetus by other medical procedures, fetus 4
[O35.7XX5] Maternal care for (suspected) damage to fetus by other medical procedures, fetus 5
[O35.7XX9] Maternal care for (suspected) damage to fetus by other medical procedures, other fetus
[O35.8XX0] Maternal care for other (suspected) fetal abnormality and damage, not applicable or unspecified
[O35.8XX1] Maternal care for other (suspected) fetal abnormality and damage, fetus 1
[O35.8XX2] Maternal care for other (suspected) fetal abnormality and damage, fetus 2
[O35.8XX3] Maternal care for other (suspected) fetal abnormality and damage, fetus 3
[O35.8XX4] Maternal care for other (suspected) fetal abnormality and damage, fetus 4
[O35.8XX5] Maternal care for other (suspected) fetal abnormality and damage, fetus 5
[O35.8XX9] Maternal care for other (suspected) fetal abnormality and damage, other fetus
[O35.9XX0] Maternal care for (suspected) fetal abnormality and damage, unspecified, not applicable or unspecified
[O35.9XX1] Maternal care for (suspected) fetal abnormality and damage, unspecified, fetus 1
[O35.9XX2] Maternal care for (suspected) fetal abnormality and damage, unspecified, fetus 2
[O35.9XX3] Maternal care for (suspected) fetal abnormality and damage, unspecified, fetus 3
[O35.9XX4] Maternal care for (suspected) fetal abnormality and damage, unspecified, fetus 4
[O35.9XX5] Maternal care for (suspected) fetal abnormality and damage, unspecified, fetus 5
[O35.9XX9] Maternal care for (suspected) fetal abnormality and damage, unspecified, other fetus
[O36.0110] Maternal care for anti-D [Rh] antibodies, first trimester, not applicable or unspecified
[O36.0111] Maternal care for anti-D [Rh] antibodies, first trimester, fetus 1
[O36.0112] Maternal care for anti-D [Rh] antibodies, first trimester, fetus 2
[O36.0113] Maternal care for anti-D [Rh] antibodies, first trimester, fetus 3

© 2024 Winterstein AG et al. *JAMA Network Open*.

[O36.0114] Maternal care for anti-D [Rh] antibodies, first trimester, fetus 4
[O36.0115] Maternal care for anti-D [Rh] antibodies, first trimester, fetus 5
[O36.0119] Maternal care for anti-D [Rh] antibodies, first trimester, other fetus
[O36.0120] Maternal care for anti-D [Rh] antibodies, second trimester, not applicable or unspecified
[O36.0121] Maternal care for anti-D [Rh] antibodies, second trimester, fetus 1
[O36.0122] Maternal care for anti-D [Rh] antibodies, second trimester, fetus 2
[O36.0123] Maternal care for anti-D [Rh] antibodies, second trimester, fetus 3
[O36.0124] Maternal care for anti-D [Rh] antibodies, second trimester, fetus 4
[O36.0125] Maternal care for anti-D [Rh] antibodies, second trimester, fetus 5
[O36.0129] Maternal care for anti-D [Rh] antibodies, second trimester, other fetus
[O36.0130] Maternal care for anti-D [Rh] antibodies, third trimester, not applicable or unspecified
[O36.0131] Maternal care for anti-D [Rh] antibodies, third trimester, fetus 1
[O36.0132] Maternal care for anti-D [Rh] antibodies, third trimester, fetus 2
[O36.0133] Maternal care for anti-D [Rh] antibodies, third trimester, fetus 3
[O36.0134] Maternal care for anti-D [Rh] antibodies, third trimester, fetus 4
[O36.0135] Maternal care for anti-D [Rh] antibodies, third trimester, fetus 5
[O36.0139] Maternal care for anti-D [Rh] antibodies, third trimester, other fetus
[O36.0190] Maternal care for anti-D [Rh] antibodies, unspecified trimester, not applicable or unspecified
[O36.0191] Maternal care for anti-D [Rh] antibodies, unspecified trimester, fetus 1
[O36.0192] Maternal care for anti-D [Rh] antibodies, unspecified trimester, fetus 2
[O36.0193] Maternal care for anti-D [Rh] antibodies, unspecified trimester, fetus 3
[O36.0194] Maternal care for anti-D [Rh] antibodies, unspecified trimester, fetus 4
[O36.0195] Maternal care for anti-D [Rh] antibodies, unspecified trimester, fetus 5
[O36.0199] Maternal care for anti-D [Rh] antibodies, unspecified trimester, other fetus
[O36.0910] Maternal care for other rhesus isoimmunization, first trimester, not applicable or unspecified
[O36.0911] Maternal care for other rhesus isoimmunization, first trimester, fetus 1
[O36.0912] Maternal care for other rhesus isoimmunization, first trimester, fetus 2
[O36.0913] Maternal care for other rhesus isoimmunization, first trimester, fetus 3
[O36.0914] Maternal care for other rhesus isoimmunization, first trimester, fetus 4
[O36.0915] Maternal care for other rhesus isoimmunization, first trimester, fetus 5
[O36.0919] Maternal care for other rhesus isoimmunization, first trimester, other fetus
[O36.0920] Maternal care for other rhesus isoimmunization, second trimester, not applicable or unspecified
[O36.0921] Maternal care for other rhesus isoimmunization, second trimester, fetus 1
[O36.0922] Maternal care for other rhesus isoimmunization, second trimester, fetus 2
[O36.0923] Maternal care for other rhesus isoimmunization, second trimester, fetus 3
[O36.0924] Maternal care for other rhesus isoimmunization, second trimester, fetus 4

© 2024 Winterstein AG et al. *JAMA Network Open*.

[O36.0925] Maternal care for other rhesus isoimmunization, second trimester, fetus 5
[O36.0929] Maternal care for other rhesus isoimmunization, second trimester, other fetus
[O36.0930] Maternal care for other rhesus isoimmunization, third trimester, not applicable or unspecified
[O36.0931] Maternal care for other rhesus isoimmunization, third trimester, fetus 1
[O36.0932] Maternal care for other rhesus isoimmunization, third trimester, fetus 2
[O36.0933] Maternal care for other rhesus isoimmunization, third trimester, fetus 3
[O36.0934] Maternal care for other rhesus isoimmunization, third trimester, fetus 4
[O36.0935] Maternal care for other rhesus isoimmunization, third trimester, fetus 5
[O36.0939] Maternal care for other rhesus isoimmunization, third trimester, other fetus
[O36.0990] Maternal care for other rhesus isoimmunization, unspecified trimester, not applicable or unspecified
[O36.0991] Maternal care for other rhesus isoimmunization, unspecified trimester, fetus 1
[O36.0992] Maternal care for other rhesus isoimmunization, unspecified trimester, fetus 2
[O36.0993] Maternal care for other rhesus isoimmunization, unspecified trimester, fetus 3
[O36.0994] Maternal care for other rhesus isoimmunization, unspecified trimester, fetus 4
[O36.0995] Maternal care for other rhesus isoimmunization, unspecified trimester, fetus 5
[O36.0999] Maternal care for other rhesus isoimmunization, unspecified trimester, other fetus
[O36.1110] Maternal care for Anti-A sensitization, first trimester, not applicable or unspecified
[O36.1111] Maternal care for Anti-A sensitization, first trimester, fetus 1
[O36.1112] Maternal care for Anti-A sensitization, first trimester, fetus 2
[O36.1113] Maternal care for Anti-A sensitization, first trimester, fetus 3
[O36.1114] Maternal care for Anti-A sensitization, first trimester, fetus 4
[O36.1115] Maternal care for Anti-A sensitization, first trimester, fetus 5
[O36.1119] Maternal care for Anti-A sensitization, first trimester, other fetus
[O36.1120] Maternal care for Anti-A sensitization, second trimester, not applicable or unspecified
[O36.1121] Maternal care for Anti-A sensitization, second trimester, fetus 1
[O36.1122] Maternal care for Anti-A sensitization, second trimester, fetus 2
[O36.1123] Maternal care for Anti-A sensitization, second trimester, fetus 3
[O36.1124] Maternal care for Anti-A sensitization, second trimester, fetus 4
[O36.1125] Maternal care for Anti-A sensitization, second trimester, fetus 5
[O36.1129] Maternal care for Anti-A sensitization, second trimester, other fetus
[O36.1130] Maternal care for Anti-A sensitization, third trimester, not applicable or unspecified
[O36.1131] Maternal care for Anti-A sensitization, third trimester, fetus 1
[O36.1132] Maternal care for Anti-A sensitization, third trimester, fetus 2
[O36.1133] Maternal care for Anti-A sensitization, third trimester, fetus 3
[O36.1134] Maternal care for Anti-A sensitization, third trimester, fetus 4
[O36.1135] Maternal care for Anti-A sensitization, third trimester, fetus 5

© 2024 Winterstein AG et al. *JAMA Network Open*.

[O36.1139] Maternal care for Anti-A sensitization, third trimester, other fetus
[O36.1190] Maternal care for Anti-A sensitization, unspecified trimester, not applicable or unspecified
[O36.1191] Maternal care for Anti-A sensitization, unspecified trimester, fetus 1
[O36.1192] Maternal care for Anti-A sensitization, unspecified trimester, fetus 2
[O36.1193] Maternal care for Anti-A sensitization, unspecified trimester, fetus 3
[O36.1194] Maternal care for Anti-A sensitization, unspecified trimester, fetus 4
[O36.1195] Maternal care for Anti-A sensitization, unspecified trimester, fetus 5
[O36.1199] Maternal care for Anti-A sensitization, unspecified trimester, other fetus
[O36.1910] Maternal care for other isoimmunization, first trimester, not applicable or unspecified
[O36.1911] Maternal care for other isoimmunization, first trimester, fetus 1
[O36.1912] Maternal care for other isoimmunization, first trimester, fetus 2
[O36.1913] Maternal care for other isoimmunization, first trimester, fetus 3
[O36.1914] Maternal care for other isoimmunization, first trimester, fetus 4
[O36.1915] Maternal care for other isoimmunization, first trimester, fetus 5
[O36.1919] Maternal care for other isoimmunization, first trimester, other fetus
[O36.1920] Maternal care for other isoimmunization, second trimester, not applicable or unspecified
[O36.1921] Maternal care for other isoimmunization, second trimester, fetus 1
[O36.1922] Maternal care for other isoimmunization, second trimester, fetus 2
[O36.1923] Maternal care for other isoimmunization, second trimester, fetus 3
[O36.1924] Maternal care for other isoimmunization, second trimester, fetus 4
[O36.1925] Maternal care for other isoimmunization, second trimester, fetus 5
[O36.1929] Maternal care for other isoimmunization, second trimester, other fetus
[O36.1930] Maternal care for other isoimmunization, third trimester, not applicable or unspecified
[O36.1931] Maternal care for other isoimmunization, third trimester, fetus 1
[O36.1932] Maternal care for other isoimmunization, third trimester, fetus 2
[O36.1933] Maternal care for other isoimmunization, third trimester, fetus 3
[O36.1934] Maternal care for other isoimmunization, third trimester, fetus 4
[O36.1935] Maternal care for other isoimmunization, third trimester, fetus 5
[O36.1939] Maternal care for other isoimmunization, third trimester, other fetus
[O36.1990] Maternal care for other isoimmunization, unspecified trimester, not applicable or unspecified
[O36.1991] Maternal care for other isoimmunization, unspecified trimester, fetus 1
[O36.1992] Maternal care for other isoimmunization, unspecified trimester, fetus 2
[O36.1993] Maternal care for other isoimmunization, unspecified trimester, fetus 3
[O36.1994] Maternal care for other isoimmunization, unspecified trimester, fetus 4
[O36.1995] Maternal care for other isoimmunization, unspecified trimester, fetus 5
[O36.1999] Maternal care for other isoimmunization, unspecified trimester, other fetus

© 2024 Winterstein AG et al. *JAMA Network Open*.

[O36.20X0] Maternal care for hydrops fetalis, unspecified trimester, not applicable or unspecified
[O36.20X1] Maternal care for hydrops fetalis, unspecified trimester, fetus 1
[O36.20X2] Maternal care for hydrops fetalis, unspecified trimester, fetus 2
[O36.20X3] Maternal care for hydrops fetalis, unspecified trimester, fetus 3
[O36.20X4] Maternal care for hydrops fetalis, unspecified trimester, fetus 4
[O36.20X5] Maternal care for hydrops fetalis, unspecified trimester, fetus 5
[O36.20X9] Maternal care for hydrops fetalis, unspecified trimester, other fetus
[O36.21X0] Maternal care for hydrops fetalis, first trimester, not applicable or unspecified
[O36.21X1] Maternal care for hydrops fetalis, first trimester, fetus 1
[O36.21X2] Maternal care for hydrops fetalis, first trimester, fetus 2
[O36.21X3] Maternal care for hydrops fetalis, first trimester, fetus 3
[O36.21X4] Maternal care for hydrops fetalis, first trimester, fetus 4
[O36.21X5] Maternal care for hydrops fetalis, first trimester, fetus 5
[O36.21X9] Maternal care for hydrops fetalis, first trimester, other fetus
[O36.22X0] Maternal care for hydrops fetalis, second trimester, not applicable or unspecified
[O36.22X1] Maternal care for hydrops fetalis, second trimester, fetus 1
[O36.22X2] Maternal care for hydrops fetalis, second trimester, fetus 2
[O36.22X3] Maternal care for hydrops fetalis, second trimester, fetus 3
[O36.22X4] Maternal care for hydrops fetalis, second trimester, fetus 4
[O36.22X5] Maternal care for hydrops fetalis, second trimester, fetus 5
[O36.22X9] Maternal care for hydrops fetalis, second trimester, other fetus
[O36.23X0] Maternal care for hydrops fetalis, third trimester, not applicable or unspecified
[O36.23X1] Maternal care for hydrops fetalis, third trimester, fetus 1
[O36.23X2] Maternal care for hydrops fetalis, third trimester, fetus 2
[O36.23X3] Maternal care for hydrops fetalis, third trimester, fetus 3
[O36.23X4] Maternal care for hydrops fetalis, third trimester, fetus 4
[O36.23X5] Maternal care for hydrops fetalis, third trimester, fetus 5
[O36.23X9] Maternal care for hydrops fetalis, third trimester, other fetus
[O36.4XX0] Maternal care for intrauterine death, not applicable or unspecified
[O36.4XX1] Maternal care for intrauterine death, fetus 1
[O36.4XX2] Maternal care for intrauterine death, fetus 2
[O36.4XX3] Maternal care for intrauterine death, fetus 3
[O36.4XX4] Maternal care for intrauterine death, fetus 4
[O36.4XX5] Maternal care for intrauterine death, fetus 5
[O36.4XX9] Maternal care for intrauterine death, other fetus
[O36.5110] Maternal care for known or suspected placental insufficiency, first trimester, not applicable or unspecified

© 2024 Winterstein AG et al. *JAMA Network Open*.

[O36.5111] Maternal care for known or suspected placental insufficiency, first trimester, fetus 1
[O36.5112] Maternal care for known or suspected placental insufficiency, first trimester, fetus 2
[O36.5113] Maternal care for known or suspected placental insufficiency, first trimester, fetus 3
[O36.5114] Maternal care for known or suspected placental insufficiency, first trimester, fetus 4
[O36.5115] Maternal care for known or suspected placental insufficiency, first trimester, fetus 5
[O36.5119] Maternal care for known or suspected placental insufficiency, first trimester, other fetus
[O36.5120] Maternal care for known or suspected placental insufficiency, second trimester, not applicable or unspecified
[O36.5121] Maternal care for known or suspected placental insufficiency, second trimester, fetus 1
[O36.5122] Maternal care for known or suspected placental insufficiency, second trimester, fetus 2
[O36.5123] Maternal care for known or suspected placental insufficiency, second trimester, fetus 3
[O36.5124] Maternal care for known or suspected placental insufficiency, second trimester, fetus 4
[O36.5125] Maternal care for known or suspected placental insufficiency, second trimester, fetus 5
[O36.5129] Maternal care for known or suspected placental insufficiency, second trimester, other fetus
[O36.5130] Maternal care for known or suspected placental insufficiency, third trimester, not applicable or unspecified
[O36.5131] Maternal care for known or suspected placental insufficiency, third trimester, fetus 1
[O36.5132] Maternal care for known or suspected placental insufficiency, third trimester, fetus 2
[O36.5133] Maternal care for known or suspected placental insufficiency, third trimester, fetus 3
[O36.5134] Maternal care for known or suspected placental insufficiency, third trimester, fetus 4
[O36.5135] Maternal care for known or suspected placental insufficiency, third trimester, fetus 5
[O36.5139] Maternal care for known or suspected placental insufficiency, third trimester, other fetus
[O36.5190] Maternal care for known or suspected placental insufficiency, unspecified trimester, not applicable or unspecified
[O36.5191] Maternal care for known or suspected placental insufficiency, unspecified trimester, fetus 1
[O36.5192] Maternal care for known or suspected placental insufficiency, unspecified trimester, fetus 2
[O36.5193] Maternal care for known or suspected placental insufficiency, unspecified trimester, fetus 3
[O36.5194] Maternal care for known or suspected placental insufficiency, unspecified trimester, fetus 4
[O36.5195] Maternal care for known or suspected placental insufficiency, unspecified trimester, fetus 5
[O36.5199] Maternal care for known or suspected placental insufficiency, unspecified trimester, other fetus
[O36.5910] Maternal care for other known or suspected poor fetal growth, first trimester, not applicable or unspecified
[O36.5911] Maternal care for other known or suspected poor fetal growth, first trimester, fetus 1
[O36.5912] Maternal care for other known or suspected poor fetal growth, first trimester, fetus 2
[O36.5913] Maternal care for other known or suspected poor fetal growth, first trimester, fetus 3
[O36.5914] Maternal care for other known or suspected poor fetal growth, first trimester, fetus 4
[O36.5915] Maternal care for other known or suspected poor fetal growth, first trimester, fetus 5
[O36.5919] Maternal care for other known or suspected poor fetal growth, first trimester, other fetus
[O36.5920] Maternal care for other known or suspected poor fetal growth, second trimester, not applicable or unspecified
[O36.5921] Maternal care for other known or suspected poor fetal growth, second trimester, fetus 1

© 2024 Winterstein AG et al. *JAMA Network Open*.

[O36.5922] Maternal care for other known or suspected poor fetal growth, second trimester, fetus 2
[O36.5923] Maternal care for other known or suspected poor fetal growth, second trimester, fetus 3
[O36.5924] Maternal care for other known or suspected poor fetal growth, second trimester, fetus 4
[O36.5925] Maternal care for other known or suspected poor fetal growth, second trimester, fetus 5
[O36.5929] Maternal care for other known or suspected poor fetal growth, second trimester, other fetus
[O36.5930] Maternal care for other known or suspected poor fetal growth, third trimester, not applicable or unspecified
[O36.5931] Maternal care for other known or suspected poor fetal growth, third trimester, fetus 1
[O36.5932] Maternal care for other known or suspected poor fetal growth, third trimester, fetus 2
[O36.5933] Maternal care for other known or suspected poor fetal growth, third trimester, fetus 3
[O36.5934] Maternal care for other known or suspected poor fetal growth, third trimester, fetus 4
[O36.5935] Maternal care for other known or suspected poor fetal growth, third trimester, fetus 5
[O36.5939] Maternal care for other known or suspected poor fetal growth, third trimester, other fetus
[O36.5990] Maternal care for other known or suspected poor fetal growth, unspecified trimester, not applicable or unspecified
[O36.5991] Maternal care for other known or suspected poor fetal growth, unspecified trimester, fetus 1
[O36.5992] Maternal care for other known or suspected poor fetal growth, unspecified trimester, fetus 2
[O36.5993] Maternal care for other known or suspected poor fetal growth, unspecified trimester, fetus 3
[O36.5994] Maternal care for other known or suspected poor fetal growth, unspecified trimester, fetus 4
[O36.5995] Maternal care for other known or suspected poor fetal growth, unspecified trimester, fetus 5
[O36.5999] Maternal care for other known or suspected poor fetal growth, unspecified trimester, other fetus
[O36.60X0] Maternal care for excessive fetal growth, unspecified trimester, not applicable or unspecified
[O36.60X1] Maternal care for excessive fetal growth, unspecified trimester, fetus 1
[O36.60X2] Maternal care for excessive fetal growth, unspecified trimester, fetus 2
[O36.60X3] Maternal care for excessive fetal growth, unspecified trimester, fetus 3
[O36.60X4] Maternal care for excessive fetal growth, unspecified trimester, fetus 4
[O36.60X5] Maternal care for excessive fetal growth, unspecified trimester, fetus 5
[O36.60X9] Maternal care for excessive fetal growth, unspecified trimester, other fetus
[O36.61X0] Maternal care for excessive fetal growth, first trimester, not applicable or unspecified
[O36.61X1] Maternal care for excessive fetal growth, first trimester, fetus 1
[O36.61X2] Maternal care for excessive fetal growth, first trimester, fetus 2
[O36.61X3] Maternal care for excessive fetal growth, first trimester, fetus 3
[O36.61X4] Maternal care for excessive fetal growth, first trimester, fetus 4
[O36.61X5] Maternal care for excessive fetal growth, first trimester, fetus 5
[O36.61X9] Maternal care for excessive fetal growth, first trimester, other fetus
[O36.62X0] Maternal care for excessive fetal growth, second trimester, not applicable or unspecified
[O36.62X1] Maternal care for excessive fetal growth, second trimester, fetus 1
[O36.62X2] Maternal care for excessive fetal growth, second trimester, fetus 2

© 2024 Winterstein AG et al. *JAMA Network Open*.

[O36.62X3] Maternal care for excessive fetal growth, second trimester, fetus 3
[O36.62X4] Maternal care for excessive fetal growth, second trimester, fetus 4
[O36.62X5] Maternal care for excessive fetal growth, second trimester, fetus 5
[O36.62X9] Maternal care for excessive fetal growth, second trimester, other fetus
[O36.63X0] Maternal care for excessive fetal growth, third trimester, not applicable or unspecified
[O36.63X1] Maternal care for excessive fetal growth, third trimester, fetus 1
[O36.63X2] Maternal care for excessive fetal growth, third trimester, fetus 2
[O36.63X3] Maternal care for excessive fetal growth, third trimester, fetus 3
[O36.63X4] Maternal care for excessive fetal growth, third trimester, fetus 4
[O36.63X5] Maternal care for excessive fetal growth, third trimester, fetus 5
[O36.63X9] Maternal care for excessive fetal growth, third trimester, other fetus
[O36.70X0] Maternal care for viable fetus in abdominal pregnancy, unspecified trimester, not applicable or unspecified
[O36.70X1] Maternal care for viable fetus in abdominal pregnancy, unspecified trimester, fetus 1
[O36.70X2] Maternal care for viable fetus in abdominal pregnancy, unspecified trimester, fetus 2
[O36.70X3] Maternal care for viable fetus in abdominal pregnancy, unspecified trimester, fetus 3
[O36.70X4] Maternal care for viable fetus in abdominal pregnancy, unspecified trimester, fetus 4
[O36.70X5] Maternal care for viable fetus in abdominal pregnancy, unspecified trimester, fetus 5
[O36.70X9] Maternal care for viable fetus in abdominal pregnancy, unspecified trimester, other fetus
[O36.71X0] Maternal care for viable fetus in abdominal pregnancy, first trimester, not applicable or unspecified
[O36.71X1] Maternal care for viable fetus in abdominal pregnancy, first trimester, fetus 1
[O36.71X2] Maternal care for viable fetus in abdominal pregnancy, first trimester, fetus 2
[O36.71X3] Maternal care for viable fetus in abdominal pregnancy, first trimester, fetus 3
[O36.71X4] Maternal care for viable fetus in abdominal pregnancy, first trimester, fetus 4
[O36.71X5] Maternal care for viable fetus in abdominal pregnancy, first trimester, fetus 5
[O36.71X9] Maternal care for viable fetus in abdominal pregnancy, first trimester, other fetus
[O36.72X0] Maternal care for viable fetus in abdominal pregnancy, second trimester, not applicable or unspecified
[O36.72X1] Maternal care for viable fetus in abdominal pregnancy, second trimester, fetus 1
[O36.72X2] Maternal care for viable fetus in abdominal pregnancy, second trimester, fetus 2
[O36.72X3] Maternal care for viable fetus in abdominal pregnancy, second trimester, fetus 3
[O36.72X4] Maternal care for viable fetus in abdominal pregnancy, second trimester, fetus 4
[O36.72X5] Maternal care for viable fetus in abdominal pregnancy, second trimester, fetus 5
[O36.72X9] Maternal care for viable fetus in abdominal pregnancy, second trimester, other fetus
[O36.73X0] Maternal care for viable fetus in abdominal pregnancy, third trimester, not applicable or unspecified
[O36.73X1] Maternal care for viable fetus in abdominal pregnancy, third trimester, fetus 1
[O36.73X2] Maternal care for viable fetus in abdominal pregnancy, third trimester, fetus 2
[O36.73X3] Maternal care for viable fetus in abdominal pregnancy, third trimester, fetus 3

© 2024 Winterstein AG et al. *JAMA Network Open*.

[O36.73X4] Maternal care for viable fetus in abdominal pregnancy, third trimester, fetus 4
[O36.73X5] Maternal care for viable fetus in abdominal pregnancy, third trimester, fetus 5
[O36.73X9] Maternal care for viable fetus in abdominal pregnancy, third trimester, other fetus
[O36.80X0] Pregnancy with inconclusive fetal viability, not applicable or unspecified
[O36.80X1] Pregnancy with inconclusive fetal viability, fetus 1
[O36.80X2] Pregnancy with inconclusive fetal viability, fetus 2
[O36.80X3] Pregnancy with inconclusive fetal viability, fetus 3
[O36.80X4] Pregnancy with inconclusive fetal viability, fetus 4
[O36.80X5] Pregnancy with inconclusive fetal viability, fetus 5
[O36.80X9] Pregnancy with inconclusive fetal viability, other fetus
[O36.8120] Decreased fetal movements, second trimester, not applicable or unspecified
[O36.8121] Decreased fetal movements, second trimester, fetus 1
[O36.8122] Decreased fetal movements, second trimester, fetus 2
[O36.8123] Decreased fetal movements, second trimester, fetus 3
[O36.8124] Decreased fetal movements, second trimester, fetus 4
[O36.8125] Decreased fetal movements, second trimester, fetus 5
[O36.8129] Decreased fetal movements, second trimester, other fetus
[O36.8130] Decreased fetal movements, third trimester, not applicable or unspecified
[O36.8131] Decreased fetal movements, third trimester, fetus 1
[O36.8132] Decreased fetal movements, third trimester, fetus 2
[O36.8133] Decreased fetal movements, third trimester, fetus 3
[O36.8134] Decreased fetal movements, third trimester, fetus 4
[O36.8135] Decreased fetal movements, third trimester, fetus 5
[O36.8139] Decreased fetal movements, third trimester, other fetus
[O36.8190] Decreased fetal movements, unspecified trimester, not applicable or unspecified
[O36.8191] Decreased fetal movements, unspecified trimester, fetus 1
[O36.8192] Decreased fetal movements, unspecified trimester, fetus 2
[O36.8193] Decreased fetal movements, unspecified trimester, fetus 3
[O36.8194] Decreased fetal movements, unspecified trimester, fetus 4
[O36.8195] Decreased fetal movements, unspecified trimester, fetus 5
[O36.8199] Decreased fetal movements, unspecified trimester, other fetus
[O36.8210] Fetal anemia and thrombocytopenia, first trimester, not applicable or unspecified
[O36.8211] Fetal anemia and thrombocytopenia, first trimester, fetus 1
[O36.8212] Fetal anemia and thrombocytopenia, first trimester, fetus 2
[O36.8213] Fetal anemia and thrombocytopenia, first trimester, fetus 3
[O36.8214] Fetal anemia and thrombocytopenia, first trimester, fetus 4

© 2024 Winterstein AG et al. *JAMA Network Open*.

[O36.8215] Fetal anemia and thrombocytopenia, first trimester, fetus 5
[O36.8219] Fetal anemia and thrombocytopenia, first trimester, other fetus
[O36.8220] Fetal anemia and thrombocytopenia, second trimester, not applicable or unspecified
[O36.8221] Fetal anemia and thrombocytopenia, second trimester, fetus 1
[O36.8222] Fetal anemia and thrombocytopenia, second trimester, fetus 2
[O36.8223] Fetal anemia and thrombocytopenia, second trimester, fetus 3
[O36.8224] Fetal anemia and thrombocytopenia, second trimester, fetus 4
[O36.8225] Fetal anemia and thrombocytopenia, second trimester, fetus 5
[O36.8229] Fetal anemia and thrombocytopenia, second trimester, other fetus
[O36.8230] Fetal anemia and thrombocytopenia, third trimester, not applicable or unspecified
[O36.8231] Fetal anemia and thrombocytopenia, third trimester, fetus 1
[O36.8232] Fetal anemia and thrombocytopenia, third trimester, fetus 2
[O36.8233] Fetal anemia and thrombocytopenia, third trimester, fetus 3
[O36.8234] Fetal anemia and thrombocytopenia, third trimester, fetus 4
[O36.8235] Fetal anemia and thrombocytopenia, third trimester, fetus 5
[O36.8239] Fetal anemia and thrombocytopenia, third trimester, other fetus
[O36.8290] Fetal anemia and thrombocytopenia, unspecified trimester, not applicable or unspecified
[O36.8291] Fetal anemia and thrombocytopenia, unspecified trimester, fetus 1
[O36.8292] Fetal anemia and thrombocytopenia, unspecified trimester, fetus 2
[O36.8293] Fetal anemia and thrombocytopenia, unspecified trimester, fetus 3
[O36.8294] Fetal anemia and thrombocytopenia, unspecified trimester, fetus 4
[O36.8295] Fetal anemia and thrombocytopenia, unspecified trimester, fetus 5
[O36.8299] Fetal anemia and thrombocytopenia, unspecified trimester, other fetus
[O36.8310] Maternal care for abnormalities of the fetal heart rate or rhythm, first trimester, not applicable or unspecified
[O36.8311] Maternal care for abnormalities of the fetal heart rate or rhythm, first trimester, fetus 1
[O36.8312] Maternal care for abnormalities of the fetal heart rate or rhythm, first trimester, fetus 2
[O36.8313] Maternal care for abnormalities of the fetal heart rate or rhythm, first trimester, fetus 3
[O36.8314] Maternal care for abnormalities of the fetal heart rate or rhythm, first trimester, fetus 4
[O36.8315] Maternal care for abnormalities of the fetal heart rate or rhythm, first trimester, fetus 5
[O36.8319] Maternal care for abnormalities of the fetal heart rate or rhythm, first trimester, other fetus
[O36.8320] Maternal care for abnormalities of the fetal heart rate or rhythm, second trimester, not applicable or unspecified
[O36.8321] Maternal care for abnormalities of the fetal heart rate or rhythm, second trimester, fetus 1
[O36.8322] Maternal care for abnormalities of the fetal heart rate or rhythm, second trimester, fetus 2
[O36.8323] Maternal care for abnormalities of the fetal heart rate or rhythm, second trimester, fetus 3
[O36.8324] Maternal care for abnormalities of the fetal heart rate or rhythm, second trimester, fetus 4
[O36.8325] Maternal care for abnormalities of the fetal heart rate or rhythm, second trimester, fetus 5

© 2024 Winterstein AG et al. *JAMA Network Open*.

[O36.8329] Maternal care for abnormalities of the fetal heart rate or rhythm, second trimester, other fetus
[O36.8330] Maternal care for abnormalities of the fetal heart rate or rhythm, third trimester, not applicable or unspecified
[O36.8331] Maternal care for abnormalities of the fetal heart rate or rhythm, third trimester, fetus 1
[O36.8332] Maternal care for abnormalities of the fetal heart rate or rhythm, third trimester, fetus 2
[O36.8333] Maternal care for abnormalities of the fetal heart rate or rhythm, third trimester, fetus 3
[O36.8334] Maternal care for abnormalities of the fetal heart rate or rhythm, third trimester, fetus 4
[O36.8335] Maternal care for abnormalities of the fetal heart rate or rhythm, third trimester, fetus 5
[O36.8339] Maternal care for abnormalities of the fetal heart rate or rhythm, third trimester, other fetus
[O36.8390] Maternal care for abnormalities of the fetal heart rate or rhythm, unspecified trimester, not applicable or unspecified
[O36.8391] Maternal care for abnormalities of the fetal heart rate or rhythm, unspecified trimester, fetus 1
[O36.8392] Maternal care for abnormalities of the fetal heart rate or rhythm, unspecified trimester, fetus 2
[O36.8393] Maternal care for abnormalities of the fetal heart rate or rhythm, unspecified trimester, fetus 3
[O36.8394] Maternal care for abnormalities of the fetal heart rate or rhythm, unspecified trimester, fetus 4
[O36.8395] Maternal care for abnormalities of the fetal heart rate or rhythm, unspecified trimester, fetus 5
[O36.8399] Maternal care for abnormalities of the fetal heart rate or rhythm, unspecified trimester, other fetus
[O36.8910] Maternal care for other specified fetal problems, first trimester, not applicable or unspecified
[O36.8911] Maternal care for other specified fetal problems, first trimester, fetus 1
[O36.8912] Maternal care for other specified fetal problems, first trimester, fetus 2
[O36.8913] Maternal care for other specified fetal problems, first trimester, fetus 3
[O36.8914] Maternal care for other specified fetal problems, first trimester, fetus 4
[O36.8915] Maternal care for other specified fetal problems, first trimester, fetus 5
[O36.8919] Maternal care for other specified fetal problems, first trimester, other fetus
[O36.8920] Maternal care for other specified fetal problems, second trimester, not applicable or unspecified
[O36.8921] Maternal care for other specified fetal problems, second trimester, fetus 1
[O36.8922] Maternal care for other specified fetal problems, second trimester, fetus 2
[O36.8923] Maternal care for other specified fetal problems, second trimester, fetus 3
[O36.8924] Maternal care for other specified fetal problems, second trimester, fetus 4
[O36.8925] Maternal care for other specified fetal problems, second trimester, fetus 5
[O36.8929] Maternal care for other specified fetal problems, second trimester, other fetus
[O36.8930] Maternal care for other specified fetal problems, third trimester, not applicable or unspecified
[O36.8931] Maternal care for other specified fetal problems, third trimester, fetus 1
[O36.8932] Maternal care for other specified fetal problems, third trimester, fetus 2
[O36.8933] Maternal care for other specified fetal problems, third trimester, fetus 3
[O36.8934] Maternal care for other specified fetal problems, third trimester, fetus 4
[O36.8935] Maternal care for other specified fetal problems, third trimester, fetus 5
[O36.8939] Maternal care for other specified fetal problems, third trimester, other fetus

© 2024 Winterstein AG et al. *JAMA Network Open*.

[O36.8990] Maternal care for other specified fetal problems, unspecified trimester, not applicable or unspecified
[O36.8991] Maternal care for other specified fetal problems, unspecified trimester, fetus 1
[O36.8992] Maternal care for other specified fetal problems, unspecified trimester, fetus 2
[O36.8993] Maternal care for other specified fetal problems, unspecified trimester, fetus 3
[O36.8994] Maternal care for other specified fetal problems, unspecified trimester, fetus 4
[O36.8995] Maternal care for other specified fetal problems, unspecified trimester, fetus 5
[O36.8999] Maternal care for other specified fetal problems, unspecified trimester, other fetus
[O36.90X0] Maternal care for fetal problem, unspecified, unspecified trimester, not applicable or unspecified
[O36.90X1] Maternal care for fetal problem, unspecified, unspecified trimester, fetus 1
[O36.90X2] Maternal care for fetal problem, unspecified, unspecified trimester, fetus 2
[O36.90X3] Maternal care for fetal problem, unspecified, unspecified trimester, fetus 3
[O36.90X4] Maternal care for fetal problem, unspecified, unspecified trimester, fetus 4
[O36.90X5] Maternal care for fetal problem, unspecified, unspecified trimester, fetus 5
[O36.90X9] Maternal care for fetal problem, unspecified, unspecified trimester, other fetus
[O36.91X0] Maternal care for fetal problem, unspecified, first trimester, not applicable or unspecified
[O36.91X1] Maternal care for fetal problem, unspecified, first trimester, fetus 1
[O36.91X2] Maternal care for fetal problem, unspecified, first trimester, fetus 2
[O36.91X3] Maternal care for fetal problem, unspecified, first trimester, fetus 3
[O36.91X4] Maternal care for fetal problem, unspecified, first trimester, fetus 4
[O36.91X5] Maternal care for fetal problem, unspecified, first trimester, fetus 5
[O36.91X9] Maternal care for fetal problem, unspecified, first trimester, other fetus
[O36.92X0] Maternal care for fetal problem, unspecified, second trimester, not applicable or unspecified
[O36.92X1] Maternal care for fetal problem, unspecified, second trimester, fetus 1
[O36.92X2] Maternal care for fetal problem, unspecified, second trimester, fetus 2
[O36.92X3] Maternal care for fetal problem, unspecified, second trimester, fetus 3
[O36.92X4] Maternal care for fetal problem, unspecified, second trimester, fetus 4
[O36.92X5] Maternal care for fetal problem, unspecified, second trimester, fetus 5
[O36.92X9] Maternal care for fetal problem, unspecified, second trimester, other fetus
[O36.93X0] Maternal care for fetal problem, unspecified, third trimester, not applicable or unspecified
[O36.93X1] Maternal care for fetal problem, unspecified, third trimester, fetus 1
[O36.93X2] Maternal care for fetal problem, unspecified, third trimester, fetus 2
[O36.93X3] Maternal care for fetal problem, unspecified, third trimester, fetus 3
[O36.93X4] Maternal care for fetal problem, unspecified, third trimester, fetus 4
[O36.93X5] Maternal care for fetal problem, unspecified, third trimester, fetus 5
[O36.93X9] Maternal care for fetal problem, unspecified, third trimester, other fetus
[O40.1XX0] Polyhydramnios, first trimester, not applicable or unspecified

© 2024 Winterstein AG et al. *JAMA Network Open*.

[O40.1XX1] Polyhydramnios, first trimester, fetus 1
[O40.1XX2] Polyhydramnios, first trimester, fetus 2
[O40.1XX3] Polyhydramnios, first trimester, fetus 3
[O40.1XX4] Polyhydramnios, first trimester, fetus 4
[O40.1XX5] Polyhydramnios, first trimester, fetus 5
[O40.1XX9] Polyhydramnios, first trimester, other fetus
[O40.2XX0] Polyhydramnios, second trimester, not applicable or unspecified
[O40.2XX1] Polyhydramnios, second trimester, fetus 1
[O40.2XX2] Polyhydramnios, second trimester, fetus 2
[O40.2XX3] Polyhydramnios, second trimester, fetus 3
[O40.2XX4] Polyhydramnios, second trimester, fetus 4
[O40.2XX5] Polyhydramnios, second trimester, fetus 5
[O40.2XX9] Polyhydramnios, second trimester, other fetus
[O40.3XX0] Polyhydramnios, third trimester, not applicable or unspecified
[O40.3XX1] Polyhydramnios, third trimester, fetus 1
[O40.3XX2] Polyhydramnios, third trimester, fetus 2
[O40.3XX3] Polyhydramnios, third trimester, fetus 3
[O40.3XX4] Polyhydramnios, third trimester, fetus 4
[O40.3XX5] Polyhydramnios, third trimester, fetus 5
[O40.3XX9] Polyhydramnios, third trimester, other fetus
[O40.9XX0] Polyhydramnios, unspecified trimester, not applicable or unspecified
[O40.9XX1] Polyhydramnios, unspecified trimester, fetus 1
[O40.9XX2] Polyhydramnios, unspecified trimester, fetus 2
[O40.9XX3] Polyhydramnios, unspecified trimester, fetus 3
[O40.9XX4] Polyhydramnios, unspecified trimester, fetus 4
[O40.9XX5] Polyhydramnios, unspecified trimester, fetus 5
[O40.9XX9] Polyhydramnios, unspecified trimester, other fetus
[O41.00X0] Oligohydramnios, unspecified trimester, not applicable or unspecified
[O41.00X1] Oligohydramnios, unspecified trimester, fetus 1
[O41.00X2] Oligohydramnios, unspecified trimester, fetus 2
[O41.00X3] Oligohydramnios, unspecified trimester, fetus 3
[O41.00X4] Oligohydramnios, unspecified trimester, fetus 4
[O41.00X5] Oligohydramnios, unspecified trimester, fetus 5
[O41.00X9] Oligohydramnios, unspecified trimester, other fetus
[O41.01X0] Oligohydramnios, first trimester, not applicable or unspecified
[O41.01X1] Oligohydramnios, first trimester, fetus 1

© 2024 Winterstein AG et al. *JAMA Network Open*.

[O41.01X2] Oligohydramnios, first trimester, fetus 2
[O41.01X3] Oligohydramnios, first trimester, fetus 3
[O41.01X4] Oligohydramnios, first trimester, fetus 4
[O41.01X5] Oligohydramnios, first trimester, fetus 5
[O41.01X9] Oligohydramnios, first trimester, other fetus
[O41.02X0] Oligohydramnios, second trimester, not applicable or unspecified
[O41.02X1] Oligohydramnios, second trimester, fetus 1
[O41.02X2] Oligohydramnios, second trimester, fetus 2
[O41.02X3] Oligohydramnios, second trimester, fetus 3
[O41.02X4] Oligohydramnios, second trimester, fetus 4
[O41.02X5] Oligohydramnios, second trimester, fetus 5
[O41.02X9] Oligohydramnios, second trimester, other fetus
[O41.03X0] Oligohydramnios, third trimester, not applicable or unspecified
[O41.03X1] Oligohydramnios, third trimester, fetus 1
[O41.03X2] Oligohydramnios, third trimester, fetus 2
[O41.03X3] Oligohydramnios, third trimester, fetus 3
[O41.03X4] Oligohydramnios, third trimester, fetus 4
[O41.03X5] Oligohydramnios, third trimester, fetus 5
[O41.03X9] Oligohydramnios, third trimester, other fetus
[O41.1010] Infection of amniotic sac and membranes, unspecified, first trimester, not applicable or unspecified
[O41.1011] Infection of amniotic sac and membranes, unspecified, first trimester, fetus 1
[O41.1012] Infection of amniotic sac and membranes, unspecified, first trimester, fetus 2
[O41.1013] Infection of amniotic sac and membranes, unspecified, first trimester, fetus 3
[O41.1014] Infection of amniotic sac and membranes, unspecified, first trimester, fetus 4
[O41.1015] Infection of amniotic sac and membranes, unspecified, first trimester, fetus 5
[O41.1019] Infection of amniotic sac and membranes, unspecified, first trimester, other fetus
[O41.1020] Infection of amniotic sac and membranes, unspecified, second trimester, not applicable or unspecified
[O41.1021] Infection of amniotic sac and membranes, unspecified, second trimester, fetus 1
[O41.1022] Infection of amniotic sac and membranes, unspecified, second trimester, fetus 2
[O41.1023] Infection of amniotic sac and membranes, unspecified, second trimester, fetus 3
[O41.1024] Infection of amniotic sac and membranes, unspecified, second trimester, fetus 4
[O41.1025] Infection of amniotic sac and membranes, unspecified, second trimester, fetus 5
[O41.1029] Infection of amniotic sac and membranes, unspecified, second trimester, other fetus
[O41.1030] Infection of amniotic sac and membranes, unspecified, third trimester, not applicable or unspecified
[O41.1031] Infection of amniotic sac and membranes, unspecified, third trimester, fetus 1
[O41.1032] Infection of amniotic sac and membranes, unspecified, third trimester, fetus 2

© 2024 Winterstein AG et al. *JAMA Network Open*.

[O41.1033] Infection of amniotic sac and membranes, unspecified, third trimester, fetus 3
[O41.1034] Infection of amniotic sac and membranes, unspecified, third trimester, fetus 4
[O41.1035] Infection of amniotic sac and membranes, unspecified, third trimester, fetus 5
[O41.1039] Infection of amniotic sac and membranes, unspecified, third trimester, other fetus
[O41.1090] Infection of amniotic sac and membranes, unspecified, unspecified trimester, not applicable or unspecified
[O41.1091] Infection of amniotic sac and membranes, unspecified, unspecified trimester, fetus 1
[O41.1092] Infection of amniotic sac and membranes, unspecified, unspecified trimester, fetus 2
[O41.1093] Infection of amniotic sac and membranes, unspecified, unspecified trimester, fetus 3
[O41.1094] Infection of amniotic sac and membranes, unspecified, unspecified trimester, fetus 4
[O41.1095] Infection of amniotic sac and membranes, unspecified, unspecified trimester, fetus 5
[O41.1099] Infection of amniotic sac and membranes, unspecified, unspecified trimester, other fetus
[O41.1210] Chorioamnionitis, first trimester, not applicable or unspecified
[O41.1211] Chorioamnionitis, first trimester, fetus 1
[O41.1212] Chorioamnionitis, first trimester, fetus 2
[O41.1213] Chorioamnionitis, first trimester, fetus 3
[O41.1214] Chorioamnionitis, first trimester, fetus 4
[O41.1215] Chorioamnionitis, first trimester, fetus 5
[O41.1219] Chorioamnionitis, first trimester, other fetus
[O41.1220] Chorioamnionitis, second trimester, not applicable or unspecified
[O41.1221] Chorioamnionitis, second trimester, fetus 1
[O41.1222] Chorioamnionitis, second trimester, fetus 2
[O41.1223] Chorioamnionitis, second trimester, fetus 3
[O41.1224] Chorioamnionitis, second trimester, fetus 4
[O41.1225] Chorioamnionitis, second trimester, fetus 5
[O41.1229] Chorioamnionitis, second trimester, other fetus
[O41.1230] Chorioamnionitis, third trimester, not applicable or unspecified
[O41.1231] Chorioamnionitis, third trimester, fetus 1
[O41.1232] Chorioamnionitis, third trimester, fetus 2
[O41.1233] Chorioamnionitis, third trimester, fetus 3
[O41.1234] Chorioamnionitis, third trimester, fetus 4
[O41.1235] Chorioamnionitis, third trimester, fetus 5
[O41.1239] Chorioamnionitis, third trimester, other fetus
[O41.1290] Chorioamnionitis, unspecified trimester, not applicable or unspecified
[O41.1291] Chorioamnionitis, unspecified trimester, fetus 1
[O41.1292] Chorioamnionitis, unspecified trimester, fetus 2
[O41.1293] Chorioamnionitis, unspecified trimester, fetus 3

© 2024 Winterstein AG et al. *JAMA Network Open*.

[O41.1294] Chorioamnionitis, unspecified trimester, fetus 4
[O41.1295] Chorioamnionitis, unspecified trimester, fetus 5
[O41.1299] Chorioamnionitis, unspecified trimester, other fetus
[O41.1410] Placentitis, first trimester, not applicable or unspecified
[O41.1411] Placentitis, first trimester, fetus 1
[O41.1412] Placentitis, first trimester, fetus 2
[O41.1413] Placentitis, first trimester, fetus 3
[O41.1414] Placentitis, first trimester, fetus 4
[O41.1415] Placentitis, first trimester, fetus 5
[O41.1419] Placentitis, first trimester, other fetus
[O41.1420] Placentitis, second trimester, not applicable or unspecified
[O41.1421] Placentitis, second trimester, fetus 1
[O41.1422] Placentitis, second trimester, fetus 2
[O41.1423] Placentitis, second trimester, fetus 3
[O41.1424] Placentitis, second trimester, fetus 4
[O41.1425] Placentitis, second trimester, fetus 5
[O41.1429] Placentitis, second trimester, other fetus
[O41.1430] Placentitis, third trimester, not applicable or unspecified
[O41.1431] Placentitis, third trimester, fetus 1
[O41.1432] Placentitis, third trimester, fetus 2
[O41.1433] Placentitis, third trimester, fetus 3
[O41.1434] Placentitis, third trimester, fetus 4
[O41.1435] Placentitis, third trimester, fetus 5
[O41.1439] Placentitis, third trimester, other fetus
[O41.1490] Placentitis, unspecified trimester, not applicable or unspecified
[O41.1491] Placentitis, unspecified trimester, fetus 1
[O41.1492] Placentitis, unspecified trimester, fetus 2
[O41.1493] Placentitis, unspecified trimester, fetus 3
[O41.1494] Placentitis, unspecified trimester, fetus 4
[O41.1495] Placentitis, unspecified trimester, fetus 5
[O41.1499] Placentitis, unspecified trimester, other fetus
[O41.8X10] Other specified disorders of amniotic fluid and membranes, first trimester, not applicable or unspecified
[O41.8X11] Other specified disorders of amniotic fluid and membranes, first trimester, fetus 1
[O41.8X12] Other specified disorders of amniotic fluid and membranes, first trimester, fetus 2
[O41.8X13] Other specified disorders of amniotic fluid and membranes, first trimester, fetus 3
[O41.8X14] Other specified disorders of amniotic fluid and membranes, first trimester, fetus 4

© 2024 Winterstein AG et al. *JAMA Network Open*.

[O41.8X15] Other specified disorders of amniotic fluid and membranes, first trimester, fetus 5
[O41.8X19] Other specified disorders of amniotic fluid and membranes, first trimester, other fetus
[O41.8X20] Other specified disorders of amniotic fluid and membranes, second trimester, not applicable or unspecified
[O41.8X21] Other specified disorders of amniotic fluid and membranes, second trimester, fetus 1
[O41.8X22] Other specified disorders of amniotic fluid and membranes, second trimester, fetus 2
[O41.8X23] Other specified disorders of amniotic fluid and membranes, second trimester, fetus 3
[O41.8X24] Other specified disorders of amniotic fluid and membranes, second trimester, fetus 4
[O41.8X25] Other specified disorders of amniotic fluid and membranes, second trimester, fetus 5
[O41.8X29] Other specified disorders of amniotic fluid and membranes, second trimester, other fetus
[O41.8X30] Other specified disorders of amniotic fluid and membranes, third trimester, not applicable or unspecified
[O41.8X31] Other specified disorders of amniotic fluid and membranes, third trimester, fetus 1
[O41.8X32] Other specified disorders of amniotic fluid and membranes, third trimester, fetus 2
[O41.8X33] Other specified disorders of amniotic fluid and membranes, third trimester, fetus 3
[O41.8X34] Other specified disorders of amniotic fluid and membranes, third trimester, fetus 4
[O41.8X35] Other specified disorders of amniotic fluid and membranes, third trimester, fetus 5
[O41.8X39] Other specified disorders of amniotic fluid and membranes, third trimester, other fetus
[O41.8X90] Other specified disorders of amniotic fluid and membranes, unspecified trimester, not applicable or unspecified
[O41.8X91] Other specified disorders of amniotic fluid and membranes, unspecified trimester, fetus 1
[O41.8X92] Other specified disorders of amniotic fluid and membranes, unspecified trimester, fetus 2
[O41.8X93] Other specified disorders of amniotic fluid and membranes, unspecified trimester, fetus 3
[O41.8X94] Other specified disorders of amniotic fluid and membranes, unspecified trimester, fetus 4
[O41.8X95] Other specified disorders of amniotic fluid and membranes, unspecified trimester, fetus 5
[O41.8X99] Other specified disorders of amniotic fluid and membranes, unspecified trimester, other fetus
[O41.90X0] Disorder of amniotic fluid and membranes, unspecified, unspecified trimester, not applicable or unspecified
[O41.90X1] Disorder of amniotic fluid and membranes, unspecified, unspecified trimester, fetus 1
[O41.90X2] Disorder of amniotic fluid and membranes, unspecified, unspecified trimester, fetus 2
[O41.90X3] Disorder of amniotic fluid and membranes, unspecified, unspecified trimester, fetus 3
[O41.90X4] Disorder of amniotic fluid and membranes, unspecified, unspecified trimester, fetus 4
[O41.90X5] Disorder of amniotic fluid and membranes, unspecified, unspecified trimester, fetus 5
[O41.90X9] Disorder of amniotic fluid and membranes, unspecified, unspecified trimester, other fetus
[O41.91X0] Disorder of amniotic fluid and membranes, unspecified, first trimester, not applicable or unspecified
[O41.91X1] Disorder of amniotic fluid and membranes, unspecified, first trimester, fetus 1
[O41.91X2] Disorder of amniotic fluid and membranes, unspecified, first trimester, fetus 2
[O41.91X3] Disorder of amniotic fluid and membranes, unspecified, first trimester, fetus 3
[O41.91X4] Disorder of amniotic fluid and membranes, unspecified, first trimester, fetus 4
[O41.91X5] Disorder of amniotic fluid and membranes, unspecified, first trimester, fetus 5

© 2024 Winterstein AG et al. *JAMA Network Open*.

[O41.91X9] Disorder of amniotic fluid and membranes, unspecified, first trimester, other fetus
[O41.92X0] Disorder of amniotic fluid and membranes, unspecified, second trimester, not applicable or unspecified
[O41.92X1] Disorder of amniotic fluid and membranes, unspecified, second trimester, fetus 1
[O41.92X2] Disorder of amniotic fluid and membranes, unspecified, second trimester, fetus 2
[O41.92X3] Disorder of amniotic fluid and membranes, unspecified, second trimester, fetus 3
[O41.92X4] Disorder of amniotic fluid and membranes, unspecified, second trimester, fetus 4
[O41.92X5] Disorder of amniotic fluid and membranes, unspecified, second trimester, fetus 5
[O41.92X9] Disorder of amniotic fluid and membranes, unspecified, second trimester, other fetus
[O41.93X0] Disorder of amniotic fluid and membranes, unspecified, third trimester, not applicable or unspecified
[O41.93X1] Disorder of amniotic fluid and membranes, unspecified, third trimester, fetus 1
[O41.93X2] Disorder of amniotic fluid and membranes, unspecified, third trimester, fetus 2
[O41.93X3] Disorder of amniotic fluid and membranes, unspecified, third trimester, fetus 3
[O41.93X4] Disorder of amniotic fluid and membranes, unspecified, third trimester, fetus 4
[O41.93X5] Disorder of amniotic fluid and membranes, unspecified, third trimester, fetus 5
[O41.93X9] Disorder of amniotic fluid and membranes, unspecified, third trimester, other fetus
[O42.00] Premature rupture of membranes, onset of labor within 24 hours of rupture, unspecified weeks of gestation
[O42.011] Preterm premature rupture of membranes, onset of labor within 24 hours of rupture, first trimester
[O42.012] Preterm premature rupture of membranes, onset of labor within 24 hours of rupture, second trimester
[O42.013] Preterm premature rupture of membranes, onset of labor within 24 hours of rupture, third trimester
[O42.019] Preterm premature rupture of membranes, onset of labor within 24 hours of rupture, unspecified trimester
[O42.02] Full-term premature rupture of membranes, onset of labor within 24 hours of rupture
[O42.10] Premature rupture of membranes, onset of labor more than 24 hours following rupture, unspecified weeks of gestation
[O42.111] Preterm premature rupture of membranes, onset of labor more than 24 hours following rupture, first trimester
[O42.112] Preterm premature rupture of membranes, onset of labor more than 24 hours following rupture, second trimester
[O42.113] Preterm premature rupture of membranes, onset of labor more than 24 hours following rupture, third trimester
[O42.119] Preterm premature rupture of membranes, onset of labor more than 24 hours following rupture, unspecified trimester
[O42.12] Full-term premature rupture of membranes, onset of labor more than 24 hours following rupture
[O42.90] Premature rupture of membranes, unspecified as to length of time between rupture and onset of labor, unspecified weeks of gestation
[O42.911] Preterm premature rupture of membranes, unspecified as to length of time between rupture and onset of labor, first trimester
[O42.912] Preterm premature rupture of membranes, unspecified as to length of time between rupture and onset of labor, second trimester
[O42.913] Preterm premature rupture of membranes, unspecified as to length of time between rupture and onset of labor, third trimester

© 2024 Winterstein AG et al. *JAMA Network Open*.

[O42.919] Preterm premature rupture of membranes, unspecified as to length of time between rupture and onset of labor, unspecified trimester
[O42.92] Full-term premature rupture of membranes, unspecified as to length of time between rupture and onset of labor
[O43.011] Fetomaternal placental transfusion syndrome, first trimester
[O43.012] Fetomaternal placental transfusion syndrome, second trimester
[O43.013] Fetomaternal placental transfusion syndrome, third trimester
[O43.019] Fetomaternal placental transfusion syndrome, unspecified trimester
[O43.021] Fetus-to-fetus placental transfusion syndrome, first trimester
[O43.022] Fetus-to-fetus placental transfusion syndrome, second trimester
[O43.023] Fetus-to-fetus placental transfusion syndrome, third trimester
[O43.029] Fetus-to-fetus placental transfusion syndrome, unspecified trimester
[O43.101] Malformation of placenta, unspecified, first trimester
[O43.102] Malformation of placenta, unspecified, second trimester
[O43.103] Malformation of placenta, unspecified, third trimester
[O43.109] Malformation of placenta, unspecified, unspecified trimester
[O43.111] Circumvallate placenta, first trimester
[O43.112] Circumvallate placenta, second trimester
[O43.113] Circumvallate placenta, third trimester
[O43.119] Circumvallate placenta, unspecified trimester
[O43.121] Velamentous insertion of umbilical cord, first trimester
[O43.122] Velamentous insertion of umbilical cord, second trimester
[O43.123] Velamentous insertion of umbilical cord, third trimester
[O43.129] Velamentous insertion of umbilical cord, unspecified trimester
[O43.191] Other malformation of placenta, first trimester
[O43.192] Other malformation of placenta, second trimester
[O43.193] Other malformation of placenta, third trimester
[O43.199] Other malformation of placenta, unspecified trimester
[O43.211] Placenta accreta, first trimester
[O43.212] Placenta accreta, second trimester
[O43.213] Placenta accreta, third trimester
[O43.219] Placenta accreta, unspecified trimester
[O43.221] Placenta increta, first trimester
[O43.222] Placenta increta, second trimester
[O43.223] Placenta increta, third trimester
[O43.229] Placenta increta, unspecified trimester
[O43.231] Placenta percreta, first trimester

© 2024 Winterstein AG et al. *JAMA Network Open*.

[O43.232] Placenta percreta, second trimester
[O43.233] Placenta percreta, third trimester
[O43.239] Placenta percreta, unspecified trimester
[O43.811] Placental infarction, first trimester
[O43.812] Placental infarction, second trimester
[O43.813] Placental infarction, third trimester
[O43.819] Placental infarction, unspecified trimester
[O43.891] Other placental disorders, first trimester
[O43.892] Other placental disorders, second trimester
[O43.893] Other placental disorders, third trimester
[O43.899] Other placental disorders, unspecified trimester
[O43.90] Unspecified placental disorder, unspecified trimester
[O43.91] Unspecified placental disorder, first trimester
[O43.92] Unspecified placental disorder, second trimester
[O43.93] Unspecified placental disorder, third trimester
[O44.00] Complete placenta previa NOS or without hemorrhage, unspecified trimester
[O44.01] Complete placenta previa NOS or without hemorrhage, first trimester
[O44.02] Complete placenta previa NOS or without hemorrhage, second trimester
[O44.03] Complete placenta previa NOS or without hemorrhage, third trimester
[O44.10] Complete placenta previa with hemorrhage, unspecified trimester
[O44.11] Complete placenta previa with hemorrhage, first trimester
[O44.12] Complete placenta previa with hemorrhage, second trimester
[O44.13] Complete placenta previa with hemorrhage, third trimester
[O44.20] Partial placenta previa NOS or without hemorrhage, unspecified trimester
[O44.21] Partial placenta previa NOS or without hemorrhage, first trimester
[O44.22] Partial placenta previa NOS or without hemorrhage, second trimester
[O44.23] Partial placenta previa NOS or without hemorrhage, third trimester
[O44.30] Partial placenta previa with hemorrhage, unspecified trimester
[O44.31] Partial placenta previa with hemorrhage, first trimester
[O44.32] Partial placenta previa with hemorrhage, second trimester
[O44.33] Partial placenta previa with hemorrhage, third trimester
[O44.40] Low lying placenta NOS or without hemorrhage, unspecified trimester
[O44.41] Low lying placenta NOS or without hemorrhage, first trimester
[O44.42] Low lying placenta NOS or without hemorrhage, second trimester
[O44.43] Low lying placenta NOS or without hemorrhage, third trimester
[O44.50] Low lying placenta with hemorrhage, unspecified trimester

© 2024 Winterstein AG et al. *JAMA Network Open*.

[O44.51] Low lying placenta with hemorrhage, first trimester
[O44.52] Low lying placenta with hemorrhage, second trimester
[O44.53] Low lying placenta with hemorrhage, third trimester
[O45.001] Premature separation of placenta with coagulation defect, unspecified, first trimester
[O45.002] Premature separation of placenta with coagulation defect, unspecified, second trimester
[O45.003] Premature separation of placenta with coagulation defect, unspecified, third trimester
[O45.009] Premature separation of placenta with coagulation defect, unspecified, unspecified trimester
[O45.011] Premature separation of placenta with afibrinogenemia, first trimester
[O45.012] Premature separation of placenta with afibrinogenemia, second trimester
[O45.013] Premature separation of placenta with afibrinogenemia, third trimester
[O45.019] Premature separation of placenta with afibrinogenemia, unspecified trimester
[O45.021] Premature separation of placenta with disseminated intravascular coagulation, first trimester
[O45.022] Premature separation of placenta with disseminated intravascular coagulation, second trimester
[O45.023] Premature separation of placenta with disseminated intravascular coagulation, third trimester
[O45.029] Premature separation of placenta with disseminated intravascular coagulation, unspecified trimester
[O45.091] Premature separation of placenta with other coagulation defect, first trimester
[O45.092] Premature separation of placenta with other coagulation defect, second trimester
[O45.093] Premature separation of placenta with other coagulation defect, third trimester
[O45.099] Premature separation of placenta with other coagulation defect, unspecified trimester
[O45.8X1] Other premature separation of placenta, first trimester
[O45.8X2] Other premature separation of placenta, second trimester
[O45.8X3] Other premature separation of placenta, third trimester
[O45.8X9] Other premature separation of placenta, unspecified trimester
[O45.90] Premature separation of placenta, unspecified, unspecified trimester
[O45.91] Premature separation of placenta, unspecified, first trimester
[O45.92] Premature separation of placenta, unspecified, second trimester
[O45.93] Premature separation of placenta, unspecified, third trimester
[O46.001] Antepartum hemorrhage with coagulation defect, unspecified, first trimester
[O46.002] Antepartum hemorrhage with coagulation defect, unspecified, second trimester
[O46.003] Antepartum hemorrhage with coagulation defect, unspecified, third trimester
[O46.009] Antepartum hemorrhage with coagulation defect, unspecified, unspecified trimester
[O46.011] Antepartum hemorrhage with afibrinogenemia, first trimester
[O46.012] Antepartum hemorrhage with afibrinogenemia, second trimester
[O46.013] Antepartum hemorrhage with afibrinogenemia, third trimester
[O46.019] Antepartum hemorrhage with afibrinogenemia, unspecified trimester
[O46.021] Antepartum hemorrhage with disseminated intravascular coagulation, first trimester

© 2024 Winterstein AG et al. *JAMA Network Open*.

[O46.022] Antepartum hemorrhage with disseminated intravascular coagulation, second trimester
[O46.023] Antepartum hemorrhage with disseminated intravascular coagulation, third trimester
[O46.029] Antepartum hemorrhage with disseminated intravascular coagulation, unspecified trimester
[O46.091] Antepartum hemorrhage with other coagulation defect, first trimester
[O46.092] Antepartum hemorrhage with other coagulation defect, second trimester
[O46.093] Antepartum hemorrhage with other coagulation defect, third trimester
[O46.099] Antepartum hemorrhage with other coagulation defect, unspecified trimester
[O46.8X1] Other antepartum hemorrhage, first trimester
[O46.8X2] Other antepartum hemorrhage, second trimester
[O46.8X3] Other antepartum hemorrhage, third trimester
[O46.8X9] Other antepartum hemorrhage, unspecified trimester
[O46.90] Antepartum hemorrhage, unspecified, unspecified trimester
[O46.91] Antepartum hemorrhage, unspecified, first trimester
[O46.92] Antepartum hemorrhage, unspecified, second trimester
[O46.93] Antepartum hemorrhage, unspecified, third trimester
[O47.00] False labor before 37 completed weeks of gestation, unspecified trimester
[O47.02] False labor before 37 completed weeks of gestation, second trimester
[O47.03] False labor before 37 completed weeks of gestation, third trimester
[O47.1] False labor at or after 37 completed weeks of gestation
[O47.9] False labor, unspecified
[O48.0] Post-term pregnancy
[O48.1] Prolonged pregnancy
[O60.00] Preterm labor without delivery, unspecified trimester
[O60.02] Preterm labor without delivery, second trimester
[O60.03] Preterm labor without delivery, third trimester
[O71.00] Rupture of uterus before onset of labor, unspecified trimester
[O71.02] Rupture of uterus before onset of labor, second trimester
[O71.03] Rupture of uterus before onset of labor, third trimester
[O71.1] Rupture of uterus during labor
[O71.2] Postpartum inversion of uterus
[O71.3] Obstetric laceration of cervix
[O71.4] Obstetric high vaginal laceration alone
[O71.5] Other obstetric injury to pelvic organs
[O71.6] Obstetric damage to pelvic joints and ligaments
[O71.7] Obstetric hematoma of pelvis
[O71.81] Laceration of uterus, not elsewhere classified

© 2024 Winterstein AG et al. *JAMA Network Open*.

[O71.82] Other specified trauma to perineum and vulva
[O71.89] Other specified obstetric trauma
[O71.9] Obstetric trauma, unspecified
[O88.011] Air embolism in pregnancy, first trimester
[O88.012] Air embolism in pregnancy, second trimester
[O88.013] Air embolism in pregnancy, third trimester
[O88.019] Air embolism in pregnancy, unspecified trimester
[O88.111] Amniotic fluid embolism in pregnancy, first trimester
[O88.112] Amniotic fluid embolism in pregnancy, second trimester
[O88.113] Amniotic fluid embolism in pregnancy, third trimester
[O88.119] Amniotic fluid embolism in pregnancy, unspecified trimester
[O88.211] Thromboembolism in pregnancy, first trimester
[O88.212] Thromboembolism in pregnancy, second trimester
[O88.213] Thromboembolism in pregnancy, third trimester
[O88.219] Thromboembolism in pregnancy, unspecified trimester
[O88.311] Pyemic and septic embolism in pregnancy, first trimester
[O88.312] Pyemic and septic embolism in pregnancy, second trimester
[O88.313] Pyemic and septic embolism in pregnancy, third trimester
[O88.319] Pyemic and septic embolism in pregnancy, unspecified trimester
[O88.811] Other embolism in pregnancy, first trimester
[O88.812] Other embolism in pregnancy, second trimester
[O88.813] Other embolism in pregnancy, third trimester
[O88.819] Other embolism in pregnancy, unspecified trimester
[O91.011] Infection of nipple associated with pregnancy, first trimester
[O91.012] Infection of nipple associated with pregnancy, second trimester
[O91.013] Infection of nipple associated with pregnancy, third trimester
[O91.019] Infection of nipple associated with pregnancy, unspecified trimester
[O91.03] Infection of nipple associated with lactation
[O91.111] Abscess of breast associated with pregnancy, first trimester
[O91.112] Abscess of breast associated with pregnancy, second trimester
[O91.113] Abscess of breast associated with pregnancy, third trimester
[O91.119] Abscess of breast associated with pregnancy, unspecified trimester
[O91.13] Abscess of breast associated with lactation
[O91.211] Nonpurulent mastitis associated with pregnancy, first trimester
[O91.212] Nonpurulent mastitis associated with pregnancy, second trimester
[O91.213] Nonpurulent mastitis associated with pregnancy, third trimester

© 2024 Winterstein AG et al. *JAMA Network Open*.

[O91.219] Nonpurulent mastitis associated with pregnancy, unspecified trimester
[O91.23] Nonpurulent mastitis associated with lactation
[O92.011] Retracted nipple associated with pregnancy, first trimester
[O92.012] Retracted nipple associated with pregnancy, second trimester
[O92.013] Retracted nipple associated with pregnancy, third trimester
[O92.019] Retracted nipple associated with pregnancy, unspecified trimester
[O92.03] Retracted nipple associated with lactation
[O92.111] Cracked nipple associated with pregnancy, first trimester
[O92.112] Cracked nipple associated with pregnancy, second trimester
[O92.113] Cracked nipple associated with pregnancy, third trimester
[O92.119] Cracked nipple associated with pregnancy, unspecified trimester
[O92.13] Cracked nipple associated with lactation
[O92.3] Agalactia
[O92.4] Hypogalactia
[O92.5] Suppressed lactation
[O92.6] Galactorrhea
[O92.70] Unspecified disorders of lactation
[O92.79] Other disorders of lactation
[O98.011] Tuberculosis complicating pregnancy, first trimester
[O98.012] Tuberculosis complicating pregnancy, second trimester
[O98.013] Tuberculosis complicating pregnancy, third trimester
[O98.019] Tuberculosis complicating pregnancy, unspecified trimester
[O98.111] Syphilis complicating pregnancy, first trimester
[O98.112] Syphilis complicating pregnancy, second trimester
[O98.113] Syphilis complicating pregnancy, third trimester
[O98.119] Syphilis complicating pregnancy, unspecified trimester
[O98.211] Gonorrhea complicating pregnancy, first trimester
[O98.212] Gonorrhea complicating pregnancy, second trimester
[O98.213] Gonorrhea complicating pregnancy, third trimester
[O98.219] Gonorrhea complicating pregnancy, unspecified trimester
[O98.311] Other infections with a predominantly sexual mode of transmission complicating pregnancy, first trimester
[O98.312] Other infections with a predominantly sexual mode of transmission complicating pregnancy, second trimester
[O98.313] Other infections with a predominantly sexual mode of transmission complicating pregnancy, third trimester
[O98.319] Other infections with a predominantly sexual mode of transmission complicating pregnancy, unspecified trimester
[O98.411] Viral hepatitis complicating pregnancy, first trimester
[O98.412] Viral hepatitis complicating pregnancy, second trimester

© 2024 Winterstein AG et al. *JAMA Network Open*.

[O98.413] Viral hepatitis complicating pregnancy, third trimester
[O98.419] Viral hepatitis complicating pregnancy, unspecified trimester
[O98.511] Other viral diseases complicating pregnancy, first trimester
[O98.512] Other viral diseases complicating pregnancy, second trimester
[O98.513] Other viral diseases complicating pregnancy, third trimester
[O98.519] Other viral diseases complicating pregnancy, unspecified trimester
[O98.611] Protozoal diseases complicating pregnancy, first trimester
[O98.612] Protozoal diseases complicating pregnancy, second trimester
[O98.613] Protozoal diseases complicating pregnancy, third trimester
[O98.619] Protozoal diseases complicating pregnancy, unspecified trimester
[O98.711] Human immunodeficiency virus [HIV] disease complicating pregnancy, first trimester
[O98.712] Human immunodeficiency virus [HIV] disease complicating pregnancy, second trimester
[O98.713] Human immunodeficiency virus [HIV] disease complicating pregnancy, third trimester
[O98.719] Human immunodeficiency virus [HIV] disease complicating pregnancy, unspecified trimester
[O98.811] Other maternal infectious and parasitic diseases complicating pregnancy, first trimester
[O98.812] Other maternal infectious and parasitic diseases complicating pregnancy, second trimester
[O98.813] Other maternal infectious and parasitic diseases complicating pregnancy, third trimester
[O98.819] Other maternal infectious and parasitic diseases complicating pregnancy, unspecified trimester
[O98.911] Unspecified maternal infectious and parasitic disease complicating pregnancy, first trimester
[O98.912] Unspecified maternal infectious and parasitic disease complicating pregnancy, second trimester
[O98.913] Unspecified maternal infectious and parasitic disease complicating pregnancy, third trimester
[O98.919] Unspecified maternal infectious and parasitic disease complicating pregnancy, unspecified trimester
[O99.011] Anemia complicating pregnancy, first trimester
[O99.012] Anemia complicating pregnancy, second trimester
[O99.013] Anemia complicating pregnancy, third trimester
[O99.019] Anemia complicating pregnancy, unspecified trimester
[O99.111] Other diseases of the blood and blood-forming organs and certain disorders involving the immune mechanism complicating pregnancy, first trimester
[O99.112] Other diseases of the blood and blood-forming organs and certain disorders involving the immune mechanism complicating pregnancy, second trimester
[O99.113] Other diseases of the blood and blood-forming organs and certain disorders involving the immune mechanism complicating pregnancy, third trimester
[O99.119] Other diseases of the blood and blood-forming organs and certain disorders involving the immune mechanism complicating pregnancy, unspecified trimester
[O99.210] Obesity complicating pregnancy, unspecified trimester
[O99.211] Obesity complicating pregnancy, first trimester

© 2024 Winterstein AG et al. *JAMA Network Open*.

[O99.212] Obesity complicating pregnancy, second trimester
[O99.213] Obesity complicating pregnancy, third trimester
[O99.280] Endocrine, nutritional and metabolic diseases complicating pregnancy, unspecified trimester
[O99.281] Endocrine, nutritional and metabolic diseases complicating pregnancy, first trimester
[O99.282] Endocrine, nutritional and metabolic diseases complicating pregnancy, second trimester
[O99.283] Endocrine, nutritional and metabolic diseases complicating pregnancy, third trimester
[O99.310] Alcohol use complicating pregnancy, unspecified trimester
[O99.311] Alcohol use complicating pregnancy, first trimester
[O99.312] Alcohol use complicating pregnancy, second trimester
[O99.313] Alcohol use complicating pregnancy, third trimester
[O99.320] Drug use complicating pregnancy, unspecified trimester
[O99.321] Drug use complicating pregnancy, first trimester
[O99.322] Drug use complicating pregnancy, second trimester
[O99.323] Drug use complicating pregnancy, third trimester
[O99.330] Smoking (tobacco) complicating pregnancy, unspecified trimester
[O99.331] Smoking (tobacco) complicating pregnancy, first trimester
[O99.332] Smoking (tobacco) complicating pregnancy, second trimester
[O99.333] Smoking (tobacco) complicating pregnancy, third trimester
[O99.340] Other mental disorders complicating pregnancy, unspecified trimester
[O99.341] Other mental disorders complicating pregnancy, first trimester
[O99.342] Other mental disorders complicating pregnancy, second trimester
[O99.343] Other mental disorders complicating pregnancy, third trimester
[O99.350] Diseases of the nervous system complicating pregnancy, unspecified trimester
[O99.351] Diseases of the nervous system complicating pregnancy, first trimester
[O99.352] Diseases of the nervous system complicating pregnancy, second trimester
[O99.353] Diseases of the nervous system complicating pregnancy, third trimester
[O99.411] Diseases of the circulatory system complicating pregnancy, first trimester
[O99.412] Diseases of the circulatory system complicating pregnancy, second trimester
[O99.413] Diseases of the circulatory system complicating pregnancy, third trimester
[O99.419] Diseases of the circulatory system complicating pregnancy, unspecified trimester
[O99.511] Diseases of the respiratory system complicating pregnancy, first trimester
[O99.512] Diseases of the respiratory system complicating pregnancy, second trimester
[O99.513] Diseases of the respiratory system complicating pregnancy, third trimester
[O99.519] Diseases of the respiratory system complicating pregnancy, unspecified trimester
[O99.611] Diseases of the digestive system complicating pregnancy, first trimester
[O99.612] Diseases of the digestive system complicating pregnancy, second trimester

© 2024 Winterstein AG et al. *JAMA Network Open*.

[O99.613] Diseases of the digestive system complicating pregnancy, third trimester
[O99.619] Diseases of the digestive system complicating pregnancy, unspecified trimester
[O99.711] Diseases of the skin and subcutaneous tissue complicating pregnancy, first trimester
[O99.712] Diseases of the skin and subcutaneous tissue complicating pregnancy, second trimester
[O99.713] Diseases of the skin and subcutaneous tissue complicating pregnancy, third trimester
[O99.719] Diseases of the skin and subcutaneous tissue complicating pregnancy, unspecified trimester
[O99.810] Abnormal glucose complicating pregnancy
[O99.820] Streptococcus B carrier state complicating pregnancy
[O99.830] Other infection carrier state complicating pregnancy
[O99.840] Bariatric surgery status complicating pregnancy, unspecified trimester
[O99.841] Bariatric surgery status complicating pregnancy, first trimester
[O99.842] Bariatric surgery status complicating pregnancy, second trimester
[O99.843] Bariatric surgery status complicating pregnancy, third trimester
[O99.891] Other specified diseases and conditions complicating pregnancy
[O9A.111] Malignant neoplasm complicating pregnancy, first trimester
[O9A.112] Malignant neoplasm complicating pregnancy, second trimester
[O9A.113] Malignant neoplasm complicating pregnancy, third trimester
[O9A.119] Malignant neoplasm complicating pregnancy, unspecified trimester
[O9A.211] Injury, poisoning and certain other consequences of external causes complicating pregnancy, first trimester
[O9A.212] Injury, poisoning and certain other consequences of external causes complicating pregnancy, second trimester
[O9A.213] Injury, poisoning and certain other consequences of external causes complicating pregnancy, third trimester
[O9A.219] Injury, poisoning and certain other consequences of external causes complicating pregnancy, unspecified trimester
[O9A.311] Physical abuse complicating pregnancy, first trimester
[O9A.312] Physical abuse complicating pregnancy, second trimester
[O9A.313] Physical abuse complicating pregnancy, third trimester
[O9A.319] Physical abuse complicating pregnancy, unspecified trimester
[O9A.411] Sexual abuse complicating pregnancy, first trimester
[O9A.412] Sexual abuse complicating pregnancy, second trimester
[O9A.413] Sexual abuse complicating pregnancy, third trimester
[O9A.419] Sexual abuse complicating pregnancy, unspecified trimester
[O9A.511] Psychological abuse complicating pregnancy, first trimester
[O9A.512] Psychological abuse complicating pregnancy, second trimester
[O9A.513] Psychological abuse complicating pregnancy, third trimester
[O9A.519] Psychological abuse complicating pregnancy, unspecified trimester
[Z03.71] Encounter for suspected problem with amniotic cavity and membrane ruled out
[Z03.72] Encounter for suspected placental problem ruled out

© 2024 Winterstein AG et al. *JAMA Network Open*.

[Z03.73] Encounter for suspected fetal anomaly ruled out
[Z03.74] Encounter for suspected problem with fetal growth ruled out
[Z03.75] Encounter for suspected cervical shortening ruled out
[Z03.79] Encounter for other suspected maternal and fetal conditions ruled out
[Z32.01] Encounter for pregnancy test, result positive
[Z34.00] Encounter for supervision of normal first pregnancy, unspecified trimester
[Z34.01] Encounter for supervision of normal first pregnancy, first trimester
[Z34.02] Encounter for supervision of normal first pregnancy, second trimester
[Z34.03] Encounter for supervision of normal first pregnancy, third trimester
[Z34.80] Encounter for supervision of other normal pregnancy, unspecified trimester
[Z34.81] Encounter for supervision of other normal pregnancy, first trimester
[Z34.82] Encounter for supervision of other normal pregnancy, second trimester
[Z34.83] Encounter for supervision of other normal pregnancy, third trimester
[Z34.90] Encounter for supervision of normal pregnancy, unspecified, unspecified trimester
[Z34.91] Encounter for supervision of normal pregnancy, unspecified, first trimester
[Z34.92] Encounter for supervision of normal pregnancy, unspecified, second trimester
[Z34.93] Encounter for supervision of normal pregnancy, unspecified, third trimester
[Z36] Encounter for antenatal screening of mother
[Z36.0] Encounter for antenatal screening for chromosomal anomalies
[Z36.1] Encounter for antenatal screening for raised alphafetoprotein level
[Z36.2] Encounter for other antenatal screening follow-up
[Z36.3] Encounter for antenatal screening for malformations
[Z36.4] Encounter for antenatal screening for fetal growth retardation
[Z36.5] Encounter for antenatal screening for isoimmunization
[Z36.81] Encounter for antenatal screening for hydrops fetalis
[Z36.82] Encounter for antenatal screening for nuchal translucency
[Z36.83] Encounter for fetal screening for congenital cardiac abnormalities
[Z36.84] Encounter for antenatal screening for fetal lung maturity
[Z36.85] Encounter for antenatal screening for Streptococcus B
[Z36.86] Encounter for antenatal screening for cervical length
[Z36.87] Encounter for antenatal screening for uncertain dates
[Z36.88] Encounter for antenatal screening for fetal macrosomia
[Z36.89] Encounter for other specified antenatal screening
[Z36.8A] Encounter for antenatal screening for other genetic defects
[Z36.9] Encounter for antenatal screening, unspecified

© 2024 Winterstein AG et al. *JAMA Network Open*.

Standalone Prenatal Visits Procedure Codes (Current Procedural Terminology or Healthcare Common Procedure Coding System)

[99500] [0500F] [0501F] [0502F] [H1000] [H1001] [H1002] [H1003] [H1004]

Practitioner Type

Medical Doctor, Osteopathic Medicine, Internal Medicine, Family Practice, Obstetrics & Gynecology, Midwife, Nurse Practitioner, Physician Assistant

© 2024 Winterstein AG et al. *JAMA Network Open*.

eTable. List of definite teratogenic medications (N = 137)

Definite Teratogenic Medications	Drug Class	1st Trimester Risk	2nd/3rd Trimester Risk
Amiodarone	Antiarrythmic	no	yes
Dronedarone	Antiarrythmic	no	yes
Dicumarol	Anticoagulant	yes	yes
Phenprocoumon	Anticoagulant	yes	yes
Warfarin	Anticoagulant	yes	yes
Carbamazepine	Anticonvulsant	yes	yes
Ethotoin	Anticonvulsant	yes	yes
Methsuximide	Anticonvulsant	yes	yes
Phenytoin / Fosphenytoin	Anticonvulsant	yes	yes
Primidone	Anticonvulsant	yes	yes
Topiramate	Anticonvulsant	yes	yes
Trimethadione / Paramethadione	Anticonvulsant	yes	yes
Valproate	Anticonvulsant	yes	yes
Azilsartan	Antihypertensive	no	yes
Benazepril	Antihypertensive	no	yes
Candesartan	Antihypertensive	no	yes
Captopril	Antihypertensive	no	yes
Enalapril	Antihypertensive	no	yes
Eprosartan	Antihypertensive	no	yes
Fosinopril	Antihypertensive	no	yes
Irbesartan	Antihypertensive	no	yes
Lisinopril	Antihypertensive	no	yes
Losartan	Antihypertensive	no	yes
Moexipril	Antihypertensive	no	yes
Olmesartan	Antihypertensive	no	yes
Perindopril	Antihypertensive	no	yes
Quinapril	Antihypertensive	no	yes

© 2024 Winterstein AG et al. *JAMA Network Open*.

Definite Teratogenic Medications	Drug Class	1st Trimester Risk	2nd/3rd Trimester Risk
Ramipril	Antihypertensive	no	yes
Telmisartan	Antihypertensive	no	yes
Trandolapril	Antihypertensive	no	yes
Valsartan	Antihypertensive	no	yes
Fluconazole	Antiinfective	yes	yes
Posaconazole	Antiinfective	yes	yes
Sulfamethoxazole & Trimethoprim	Antiinfective	yes	yes
Voriconazole	Antiinfective	yes	yes
Afatinib	Antineoplastic	yes	yes
Aflibercept	Antineoplastic	yes	yes
Altretamine	Antineoplastic	yes	yes
Asparaginase	Antineoplastic	yes	yes
Axitinib	Antineoplastic	yes	yes
Azacitidine	Antineoplastic	yes	yes
Bendamustine	Antineoplastic	yes	yes
Bevacizumab	Antineoplastic	yes	yes
Bleomycin	Antineoplastic	yes	yes
Bortezomib	Antineoplastic	yes	yes
Bosutinib	Antineoplastic	yes	yes
Cabazitaxel	Antineoplastic	yes	yes
Cabozantinib	Antineoplastic	yes	yes
Capecitabine	Antineoplastic	yes	yes
Carmustine	Antineoplastic	yes	yes
Ceritinib	Antineoplastic	yes	yes
Cetuximab	Antineoplastic	yes	yes
Cladribine	Antineoplastic	yes	yes
Clofarabine	Antineoplastic	yes	yes
Cytarabine	Antineoplastic	yes	yes
Dabrafenib	Antineoplastic	yes	yes

© 2024 Winterstein AG et al. *JAMA Network Open*.

Definite Teratogenic Medications	Drug Class	1st Trimester Risk	2nd/3rd Trimester Risk
Dacarbazine	Antineoplastic	yes	yes
Dasatinib	Antineoplastic	yes	yes
Daunorubicin	Antineoplastic	yes	yes
Decitabine	Antineoplastic	yes	yes
Docetaxel	Antineoplastic	yes	yes
Doxorubicin	Antineoplastic	yes	yes
Eribulin	Antineoplastic	yes	yes
Erlotinib	Antineoplastic	yes	yes
Etoposide	Antineoplastic	yes	yes
Floxuridine	Antineoplastic	yes	yes
Fludarabine	Antineoplastic	yes	yes
Fluorouracil	Antineoplastic	yes	yes
Gefitinib	Antineoplastic	yes	yes
Hydroxyurea	Antineoplastic	yes	yes
Ibrutinib	Antineoplastic	yes	yes
Ifosfamide	Antineoplastic	yes	yes
Imatinib	Antineoplastic	yes	yes
Irinotecan	Antineoplastic	yes	yes
Ixabepilone	Antineoplastic	yes	yes
Lapatinib	Antineoplastic	yes	yes
Lenalidomide	Antineoplastic	yes	yes
Lenvatinib	Antineoplastic	yes	yes
Lestaurtinib	Antineoplastic	yes	yes
Lomustine	Antineoplastic	yes	yes
Mechlorethamine	Antineoplastic	yes	yes
Melphalan	Antineoplastic	yes	yes
Mitomycin	Antineoplastic	yes	yes
Mitotane	Antineoplastic	yes	yes
Nelarabine	Antineoplastic	yes	yes

© 2024 Winterstein AG et al. *JAMA Network Open*.

Definite Teratogenic Medications	Drug Class	1st Trimester Risk	2nd/3rd Trimester Risk
Nilotinib	Antineoplastic	yes	yes
Nintedanib	Antineoplastic	yes	yes
Oxaliplatin	Antineoplastic	yes	yes
Panitumumab	Antineoplastic	yes	yes
Panobinostat	Antineoplastic	yes	yes
Pazopanib	Antineoplastic	yes	yes
Pemetrexed	Antineoplastic	yes	yes
Pertuzumab	Antineoplastic	yes	yes
Pomalidomide	Antineoplastic	yes	yes
Ponatinib	Antineoplastic	yes	yes
Pralatrexate	Antineoplastic	yes	yes
Ramucirumab	Antineoplastic	yes	yes
Regorafenib	Antineoplastic	yes	yes
Romidepsin	Antineoplastic	yes	yes
Ruxolitinib	Antineoplastic	yes	yes
Sorafenib	Antineoplastic	yes	yes
Sunitinib	Antineoplastic	yes	yes
Talimogene Laherparepvec	Antineoplastic	yes	yes
Temozolomide	Antineoplastic	yes	yes
Thalidomide	Antineoplastic	yes	yes
Tofacitinib	Antineoplastic	yes	yes
Topotecan	Antineoplastic	yes	yes
Trabectedin	Antineoplastic	yes	yes
Trametinib	Antineoplastic	yes	yes
Trastuzumab	Antineoplastic	yes	yes
Vandetanib	Antineoplastic	yes	yes
Vemurafenib	Antineoplastic	yes	yes
Vincristine	Antineoplastic	yes	yes
Vismodegib	Antineoplastic	yes	yes

© 2024 Winterstein AG et al. *JAMA Network Open*.

Definite Teratogenic Medications	Drug Class	1st Trimester Risk	2nd/3rd Trimester Risk
Vorinostat	Antineoplastic	yes	yes
Acitretin	Dermatologic	yes	yes
Alitretinoin	Dermatologic	yes	yes
Bexarotene	Dermatologic	yes	yes
Isotretinoin	Dermatologic	yes	yes
Tretinoin	Dermatologic	yes	yes
Acetohydroxamic Acid	Genitourinary	yes	yes
Danazol	Genitourinary	yes	yes
Dutasteride	Genitourinary	yes	yes
Dutasteride & Tamsulosin	Genitourinary	yes	yes
Finasteride	Genitourinary	yes	yes
Everolimus	Immunomodulator	yes	yes
Leflunomide	Immunomodulator	yes	yes
Mycophenolate Mofetil	Immunomodulator	yes	yes
Sirolimus	Immunomodulator	yes	yes
Temsirolimus	Immunomodulator	yes	yes
Vedolizumab	Immunomodulator	yes	yes
Iodine-131	Iodine-131	no	yes
Penicillamine	Musculoskeletal	yes	yes
Ambrisentan	Pulmonary_Hypertension	yes	yes
Bosentan	Pulmonary_Hypertension	yes	yes
Macitentan	Pulmonary_Hypertension	yes	yes
Riociguat	Pulmonary_Hypertension	yes	yes

© 2024 Winterstein AG et al. *JAMA Network Open*.

eFigure 1. Assessments of Timing of Prenatal Care Initiation Relative to Prenatal Exposure to Teratogenic Medications

For a given gestational week (e.g., week 15), all pregnancies with prenatal exposure to a teratogenic medication between conception and the given gestational week (evaluated gestational period) are categorized based on timing of prenatal care initiation:

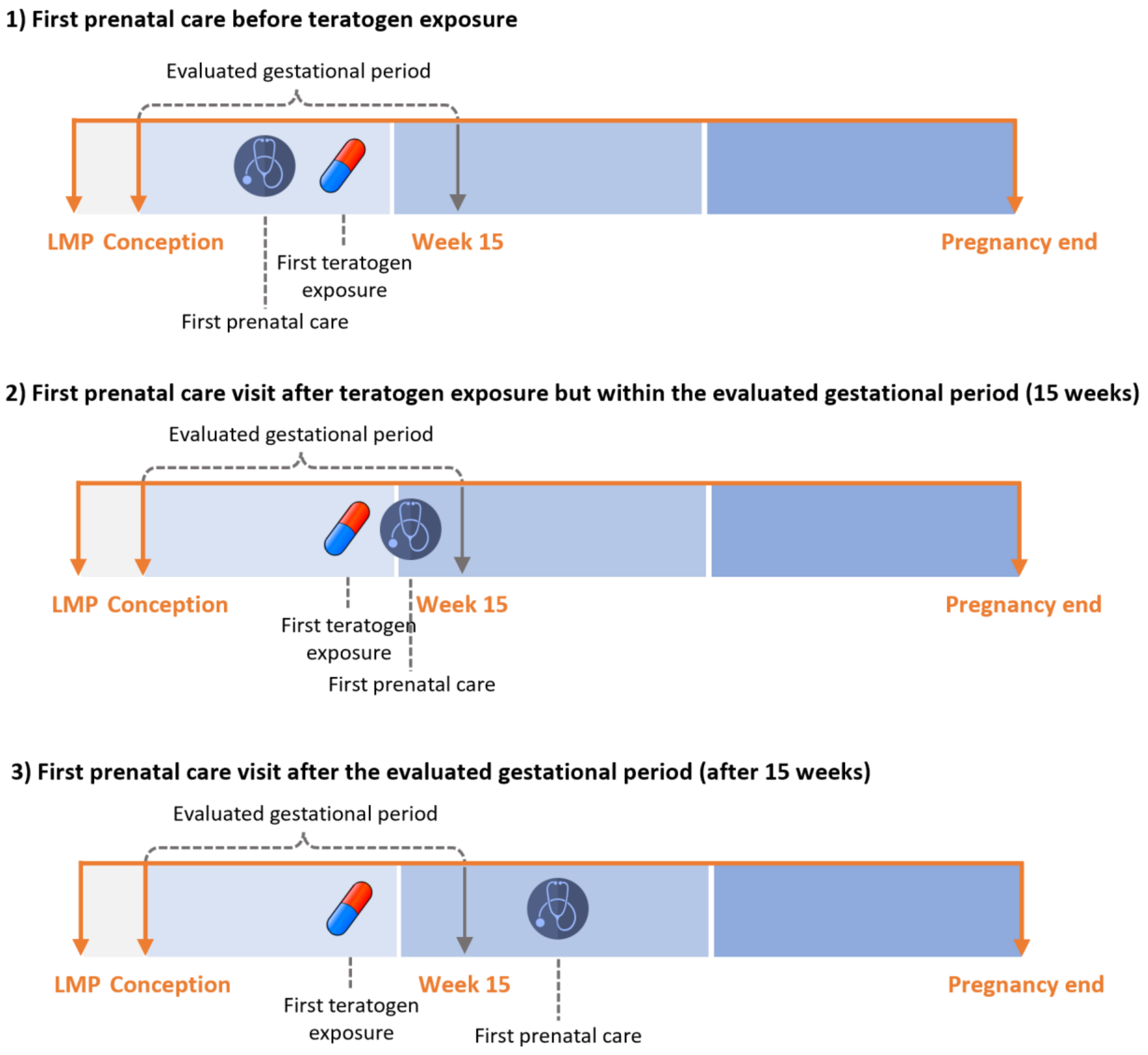

© 2024 Winterstein AG et al. *JAMA Network Open*.

eFigure 2. Timing of Prenatal Care Initiation and Prenatal Exposure to Teratogenic Medications

The figure below shows the cumulative proportion of pregnancies with prenatal care and exposure to teratogenic medications within given gestational weeks. Please access the interactive version here.

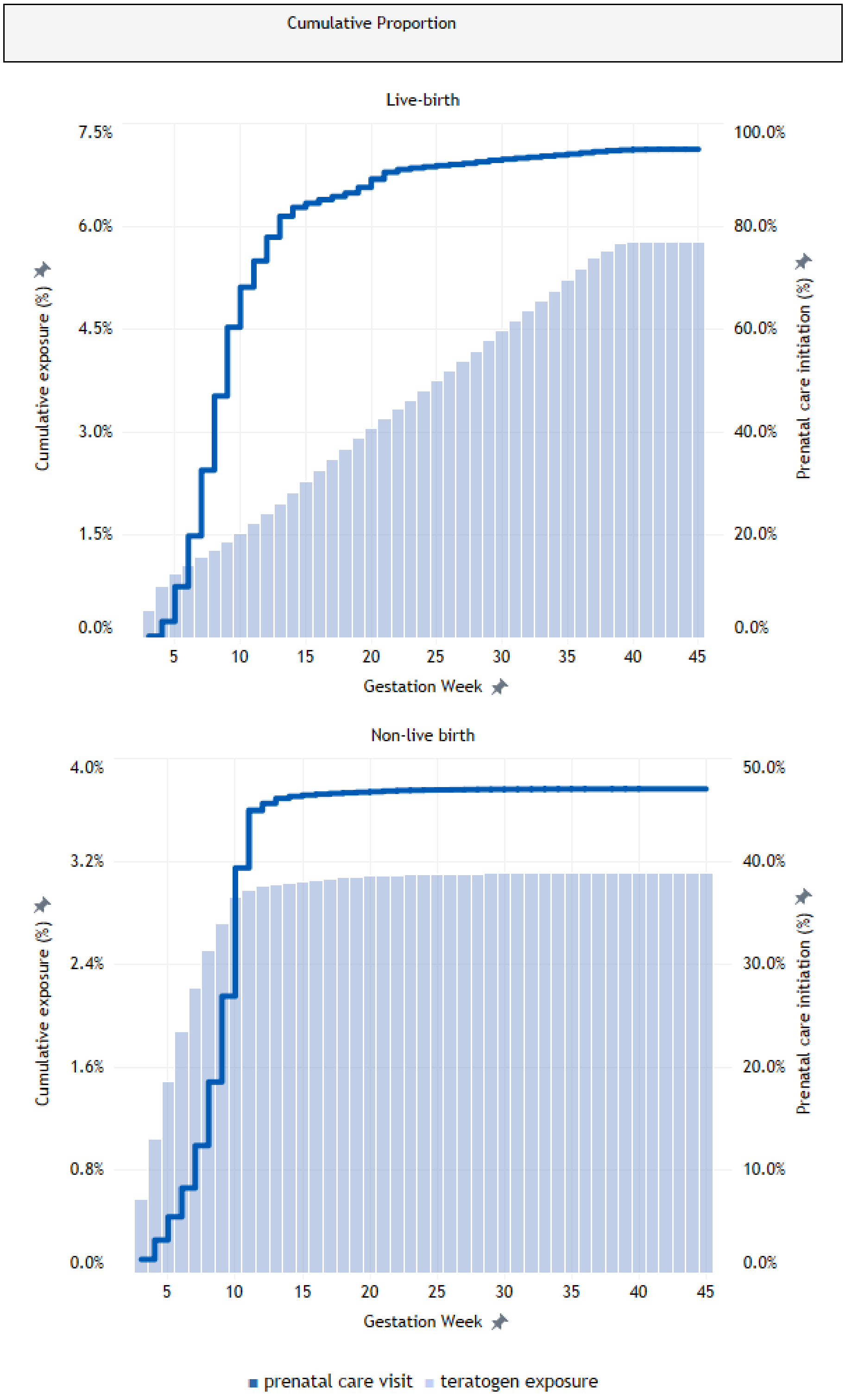

© 2024 Winterstein AG et al. *JAMA Network Open*.

eFigure 3. Timing of Prenatal Care Initiation for Pregnancies Exposed to Teratogenic Medications Considering Any Pregnancy

The figure below shows the timing of prenatal care initiation for pregnancies exposed to teratogenic medications within 15 weeks. Please access the interactive version here to view the results considering the residual days' supply and for other specific gestational weeks.

Definition	Exposure within X Gestational Weeks (LMP)
Rx Fill in the Risk Window	15

	Live birth	Non-live birth	Overall
1st prenatal care before 1st teratogen exposure	4,348 (40.3%)	465 (9.1%)	4,813 (30.3%)
1st prenatal care after 1st teratogen exposure but within given weeks	4,635 (42.9%)	1,665 (32.6%)	6,300 (39.6%)
1st prenatal care after given weeks or no prenatal care	1,810 (16.8%)	2,975 (58.3%)	4,785 (30.1%)
Pregnancies exposed to teratogens, No. (% of total pregnancies)	10,793 (2.3%)<br>472,472	5,105 (3.0%)<br>167,522	15,898 (2.5%)<br>639,994

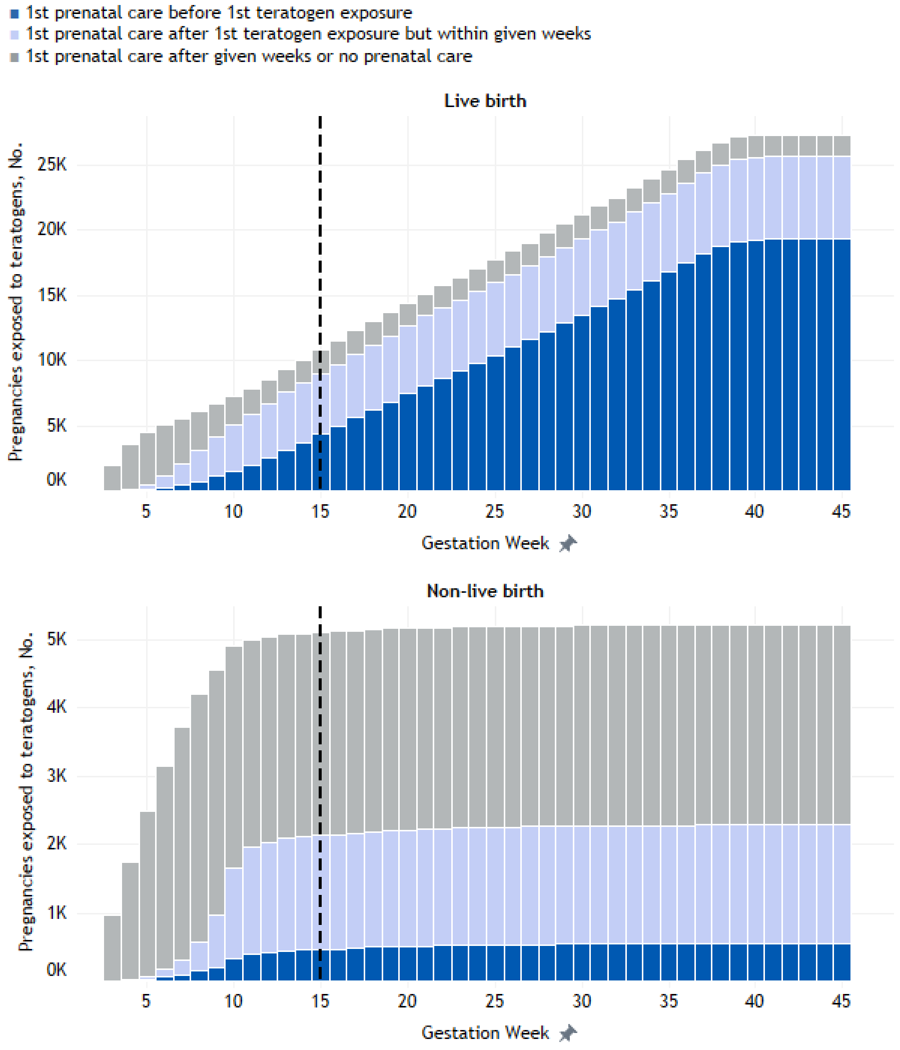

© 2024 Winterstein AG et al. *JAMA Network Open*.

eFigure 4. Timing of Prenatal Care Initiation for Pregnancies Exposed to Teratogenic Medications Considering Pregnancies Viable Up to the Assessed Gestational Week

The figure below shows the timing of prenatal care initiation for pregnancies exposed to teratogenic medications within 15 weeks, restricting to pregnancies viable at 15 weeks. Please access the interactive version here to view the results considering the residual days' supply and for other specific gestational weeks.

Definition	Exposure within X Gestational Weeks (LMP)
Rx Fill in the Risk Window	15

	Live birth	Non-live birth	Overall
1st prenatal care before 1st teratogen exposure	4,348 (40.3%)	79 (24.9%)	4,427 (39.8%)
1st prenatal care after 1st teratogen exposure but within given weeks	4,635 (42.9%)	139 (43.8%)	4,774 (43.0%)
1st prenatal care after given weeks or no prenatal care	1,810 (16.8%)	99 (31.2%)	1,909 (17.2%)
Pregnancies exposed to teratogens, No. (% of total pregnancies)	10,793 (2.3%) 472,472	317 (3.4%) 9,367	11,110 (2.3%) 481,839

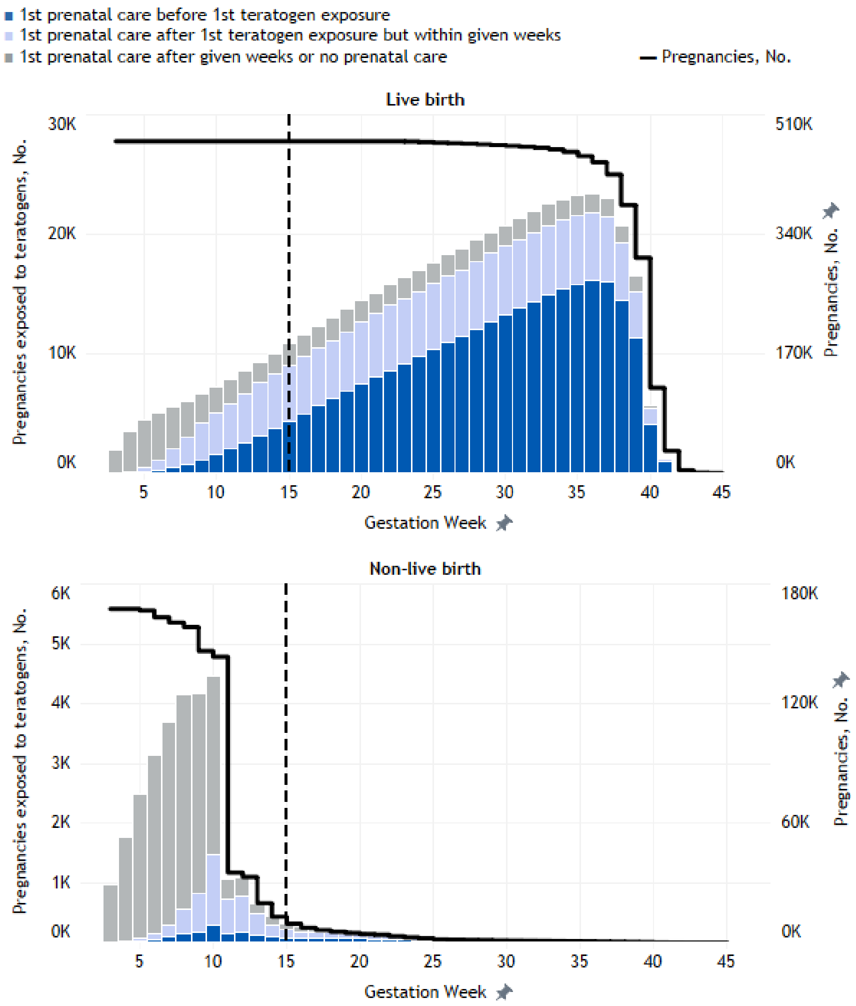

© 2024 Winterstein AG et al. *JAMA Network Open*.

eFigure 5. Timing of Prenatal Care Initiation for Pregnancies Exposed to Teratogenic Medications by Drug Class

The figure below shows the timing of prenatal care initiation for pregnancies exposed to teratogenic medications by drug class. Please access the interactive version here to view the results considering the residual days' supply and for other specific gestational weeks.

Definition	Exposure within X Gestational Weeks (LMP)
Rx Fill in the Risk Window	15

		1st prenatal care before 1st teratogen exposure	1st prenatal care after 1st teratogen exposure but within given weeks	1st prenatal care after given weeks or no prenatal care	Overall
Live birth	antiarrythmic	0 (0.0%)	0 (0.0%)	1 (100.0%)	1
	anticoagulant	3 (7.3%)	26 (63.4%)	12 (29.3%)	41
	anticonvulsant	103 (13.0%)	511 (64.4%)	179 (22.6%)	793
	antihypertensive	28 (21.7%)	2 (1.6%)	99 (76.7%)	129
	antiinfective	4,197 (43.0%)	4,046 (41.5%)	1,511 (15.5%)	9,754
	antineoplastic	8 (17.4%)	17 (37.0%)	21 (45.7%)	46
	dermatologic	0 (0.0%)	7 (70.0%)	3 (30.0%)	10
	genitourinary	2 (66.7%)	0 (0.0%)	1 (33.3%)	3
	immunomodulator	25 (24.5%)	56 (54.9%)	21 (20.6%)	102
	iodine-131	0	0	0	0
	pulmonary hypertension	0	0	0	0
Non-live birth	antiarrythmic	0	0	0	0
	anticoagulant	2 (5.3%)	18 (47.4%)	18 (47.4%)	38
	anticonvulsant	19 (3.6%)	193 (36.7%)	314 (59.7%)	526
	antihypertensive	0 (0.0%)	1 (14.3%)	6 (85.7%)	7
	antiinfective	439 (10.0%)	1,434 (32.6%)	2,522 (57.4%)	4,395
	antineoplastic	3 (6.3%)	12 (25.0%)	33 (68.8%)	48
	dermatologic	0 (0.0%)	6 (10.2%)	53 (89.8%)	59
	genitourinary	0 (0.0%)	0 (0.0%)	3 (100.0%)	3
	immunomodulator	6 (7.1%)	16 (18.8%)	63 (74.1%)	85
	iodine-131	0	0	0	0
	pulmonary hypertension	0 (0.0%)	1 (33.3%)	2 (66.7%)	3

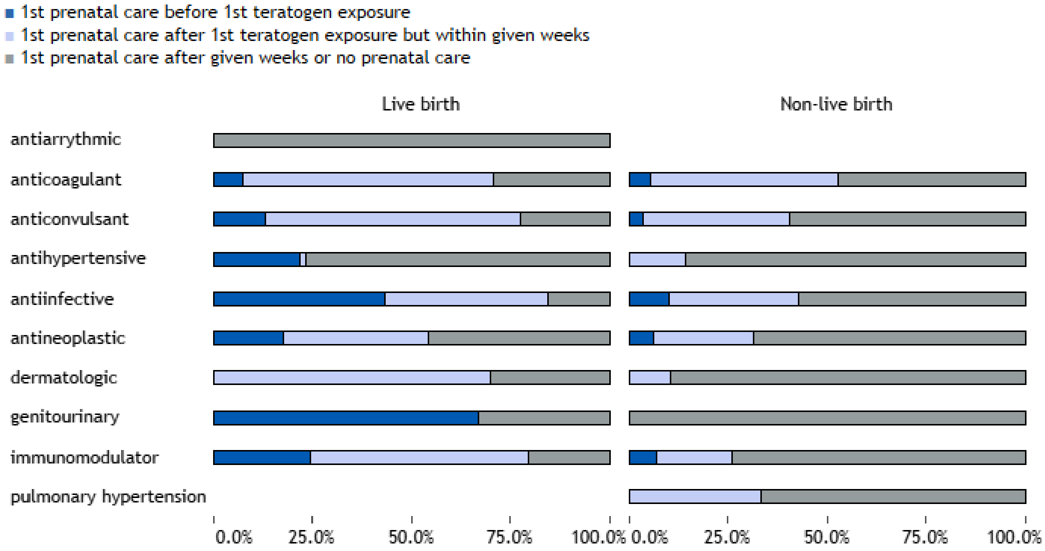

© 2024 Winterstein AG et al. *JAMA Network Open*.
