# Supplementary material for: Prenatal Care Initiation and Exposure to Teratogenic Medications
Source: JAMA Netw Open. 2024 Feb 1;7(2):e2354298. doi: 10.1001/jamanetworkopen.2023.54298 (PMC10835507; doi:10.1001/jamanetworkopen.2023.54298)
Supplement: Supplement 2. — Data Sharing Statement [file jamanetwopen-e2354298-s002.pdf]

Data Sharing Statement

Winterstein. Prenatal Care Initiation and Exposure to Teratogenic Medications. *JAMA Netw Open*. Published February 01, 2024. doi:10.1001/jamanetworkopen.2023.54298

Data
Data available: No

Additional Information
Explanation for why data not available: The authors are not permitted to share the claims data according to the data use licensure.
